# Supplementary material for: Phase II trial of neoadjuvant sitravatinib plus nivolumab in patients undergoing nephrectomy for locally advanced clear cell renal cell carcinoma
Source: Nat Commun. 2023 May 10;14:2684. doi: 10.1038/s41467-023-38342-7 (PMC10172300; doi:10.1038/s41467-023-38342-7)
Supplement: Supplementary file 1 — Supplementary Information [file 41467_2023_38342_MOESM1_ESM.pdf]

Supplementary Information

Phase II Trial of Neoadjuvant Sitravatinib Plus Nivolumab in Patients Undergoing Nephrectomy for Locally Advanced Clear Cell Renal Cell Carcinoma

Jose A. Karam MD[1,2,6,7], Pavlos Msaouel MD[2,3,4,6,7], Cara L. Haymaker PhD[2], Surena F. Matin MD[1], Matthew T. Campbell MD[3], Amado J. Zurita MD[3], Amishi Y. Shah MD[3], Ignacio I. Wistuba MD[2], Enrica Marmonti PhD[2], Dzifa Y. Duose PhD[2], Edwin R. Parra MD[2], Luisa Maren Solis Soto MD[2], Caddie Laberiano-Fernandez MD[2], Marisa Lozano OCN[1], Alice Abraham MS[1], Max Hallin MAS[5], Curtis D. Chin MD[5], Peter Olson PhD5, Hirak Der-Torossian MD[5], Xiaohong Yan PhD[5], Nizar M. Tannir MD[3], Christopher G. Wood, MD[1†]

[1]Department of Urology, The University of Texas MD Anderson Cancer Center, Houston, TX, 77030, USA.
[2]Department of Translational Molecular Pathology, The University of Texas MD Anderson Cancer Center, Houston, TX, 77030, USA.
[3]Department of Genitourinary Medical Oncology, The University of Texas MD Anderson Cancer Center, Houston, TX, USA.
[4]David H. Koch Center for Applied Research of Genitourinary Cancers, The University of Texas, MD Anderson Cancer Center, Houston, TX 77030, USA.
[5]Mirati Therapeutics, Inc., San Diego, CA, 92121, USA.
[6]These authors jointly supervised this work
[7]These authors contributed equally
†deceased

Corresponding Authors:
Jose A. Karam, MD
The University of Texas MD Anderson Cancer Center,
1515 Holcombe Blvd, Houston, TX 77030
Email: jakaram@mdanderson.org, Telephone: 713-792-3250

Pavlos Msaouel, MD, PhD
Department of Genitourinary Medical Oncology, Unit 1374
The University of Texas MD Anderson Cancer Center
1155 Pressler St
Houston, TX 77030-3721
Fax: (713) 745-7575
E-mail: PMsaouel@mdanderson.org

Supplementary Table S1. Tumoral PD-L1 expression from baseline to the time of surgery (N=21)

CORE ID	Tumour cells expressing PD-L1 (%)			PD-L1 change from baseline to time of surgery (%)	Change in sum of target lesions (%)
	Baseline	Post-sitravatinib	Post-combination (surgery)		
1	0	0	5	+5	-31
2*	0	0	0	0	N/A
3	N/A	N/A	N/A	N/A	-14
4	0	0	0	0	-12
5	0	0	NE	N/A	-17
6	0	NE	2	+2	-21
7**	0	N/A	N/A	N/A	N/A
8**	50	N/A	N/A	N/A	N/A
10	0	0	1	+1	-33
12	0	N/A	0	0	-1
13	0	0	0	0	-11
14	N/A	0	0	N/A	-28
15	N/A	0	0	N/A	-4
18*	0	0	0	0	N/A
19	0	0	0	0	0
22	0	0	N/A	N/A	0
24	0	0	20	+20	-28
26*	0	5	N/A	N/A	N/A
28	0	N/A	N/A	N/A	-12
29	40	NE	N/A	N/A	-10
30	0	0	N/A	N/A	-27
31	0	N/A	0	0	-24

N/A, not available; NE, not evaluable; PD-L1, programmed cell death ligand; *not in clinical activity evaluable population; **not in safety population nor clinical activity evaluable population; enrolled but excluded due to ineligible histology

Supplementary Table S2. Cyanine Dye-Labelled Antibodies Used for Multiplex IF.

Antibody	Clone	Vendor	Catalog#	Paired Fluorophore	Concentration
Arginase	EPR6672(B)	Abcam	ab211961	Cy5	5ug/ml
CD3	F7.2.38	Dako	M7254	Cy3	10ug/mL
CD4	EPR6855	Abcam	ab181724	Cy3	5ug/ml
CD8	C8/144B	Dako	M7103	Cy3	10ug/ml
CD11b	238439	R&D Systems	MAB16992	Cy3	10ug/ml
CD14	EPR3652	Abcam	ab209971	Cy3	5ug/ml
CD15	Carb-3	Dako	M3631	Cy3	1.5ug/ml
CD16	DJ130c	Thermo	# MA1-84008S4	Cy5	5ug/ml
CD33	44M12D3	Novus	NBP2-22377	Cy5	7.5ug/ml
CD56	MRQ-42	Cell Marque	156R-OEM0714	Cy5	2ug/ml
CD68	KP-1	BioLegend	MS-397-PABX	Cy5	0.5ug/ml
CD163	EDHu-1	BioRad	MCA1853	Cy5	1.25ug/ml
CTLA4	F-8	Santa Cruz	sc-376016	Cy3	2ug/ml
FOXP3	206D	BioLegend	320114	Cy5	5ug/ml
HLA-DR	WR18	Novus	MA1-80678	Cy5	5ug/ml
Ki67	SP6	Abcam	ab197547	Cy3	10ug/ml
PD-1	EPR4877(2)	Abcam	ab186928	Cy5	5ug/ml
PD-L1	SP142	Abcam	ab236238	Cy5	5ug/ml
PanCK	PCK26	Sigma	C5992	Cy3	1.5ug/ml
	AE1	eBio	cust02300	Cy3	2.5ug/ml

Supplementary Table S3. T Cell Functional Panel Design Used for Flow Cytometry.

Antibody	Company	Catalog	Clone	Used amount (Volume [uL]/sample)
TIGIT FITC	Life Technologies	11-9500-42	MBSA43	5
CD3 PerCP-Cy5.5	BD Biosciences	340949	SK7	10
Live/Dead Yellow	Life Technologies	L-34968		1
TIM-3 (CD366) BV605	Biolegend	345018	F38-2E2	4
PD-1 (CD279) BV650	BD Biosciences	564104	EH12	3
OX40 (CD134) BV711	BD Biosciences	563664	ACT35	5
CTLA-4 (CD152) BV786	BD Biosciences	563931	BNI3	3
CD45 BUV395	BD Biosciences	563792	HI30	5
CD4 BUV496	BD Biosciences	612936	SK3	5
CD8 Alexa Fluor 700	BD Biosciences	557945	RPA-T8	5
CD25 APC-eFluor 780	Life Technologies	47-0259-42	BC96	5
Lag3 (CD223) PE	Life Technologies	12-2239-42	3DS223H	5
CD56 PE-CF594	BD Biosciences	562289	B159	5
ICOS (CD278) PE-Cy7	Life Technologies	25-9948-42	ISA-3	3
FoxP3 eFluor 450	Life Technologies	48-4776-42	PCH101	5

Ki67 APC	Life Technologies	17-5699-42	20Raj1	5

Supplementary Figure S1. Intratumoural density of T cells at baseline, post-sitravatinib, and at surgery post-combination therapy. Multiplex IF results showing CD3+ TIL densities at baseline, post-sitravatinib monotherapy, and post-combination therapy. Statistical significance between time points assessed was determined using a two-tailed, Wilcoxon matched pairs signed rank test and is indicated with a line at the top of the graph. The median density is indicated with the black line. The number of samples analysed per time point is shown for each graph. Source data are provided in the Source Data file.

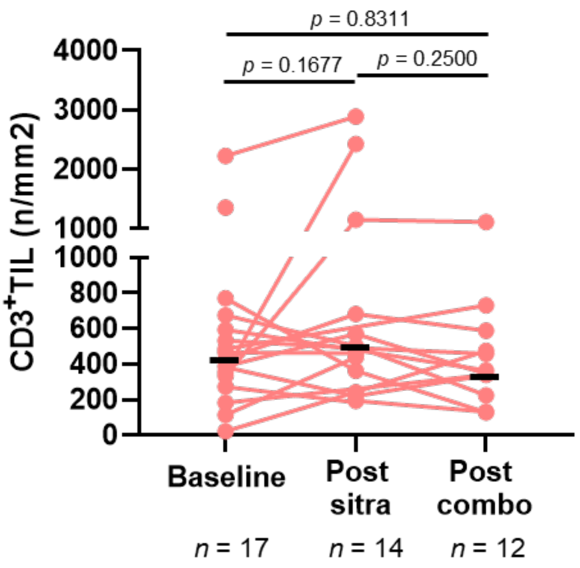

CD, cluster of differentiation; IF, immunofluorescence; TIL, tumour-infiltrating lymphocytes

Supplementary Figure S2. Immune suppressive pathways are induced by/associated with sitravatinib and nivolumab. a-d Flow cytometry analyses of tumour tissue collected at baseline (**a-b**) or longitudinally at baseline, post-sitravatinib monotherapy, and at surgery post-combination therapy (**c-d**). Expression of checkpoint receptors PD1, Tim3 and LAG3 on CD3+ CD8+ TIL (**a**) and on CD3+ CD4+ TIL (**b**). The percentage of Tim3 expression on CD8+ T cells (left) and CD4+ T cells (right) (**c**). The percentage of LAG3 expression on CD8+ T cells (left) and CD4+ T cells (right) (**d**). **e-f** Multiplex IF results showing CD3+LAG3+ TIL densities at baseline, post-sitravatinib monotherapy, and post-combination therapy (**e**) with a representative image (**f**). The median density is indicated with the black line. Statistical significance between time points assessed was determined using a two-tailed, Wilcoxon matched pairs signed rank test. The number of samples analysed per time point is shown for each graph. Source data are provided in the Source Data file.

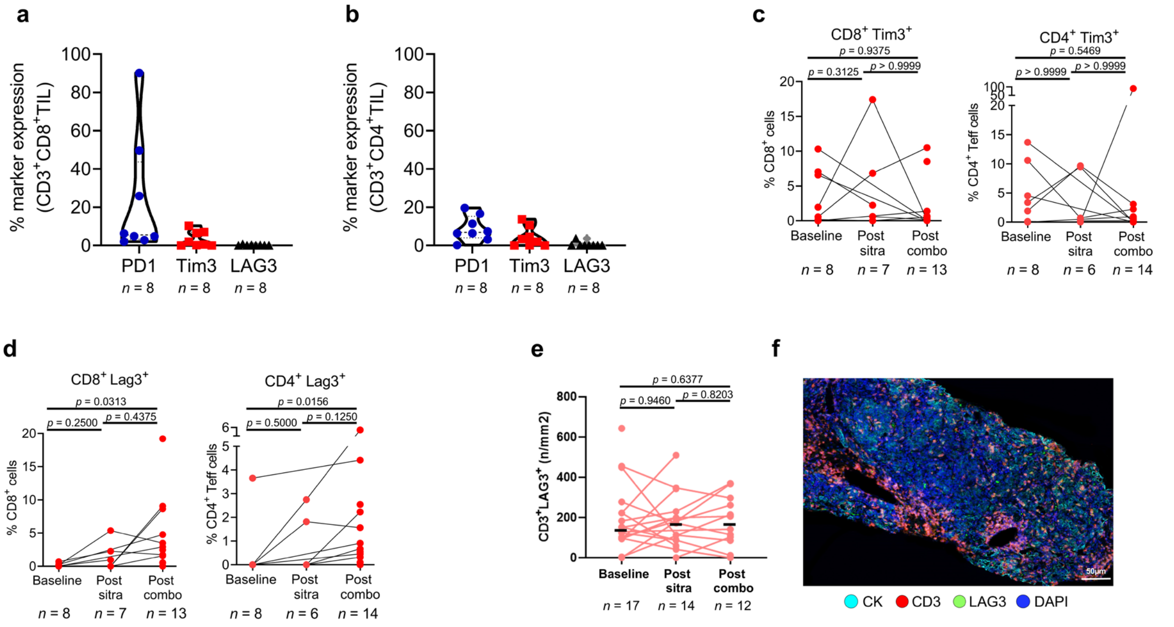

CD, cluster of differentiation; IF, immunofluorescence; PD-1, programmed cell death 1; TIL, tumour infiltrating lymphocytes.

Supplementary Figure S3. Immune activation induced by/associated with sitravatinib and nivolumab. a-b Flow cytometry analyses of tumour tissue collected longitudinally at baseline, post-sitravatinib monotherapy, and at surgery post-combination therapy. Expression of ICOS on CD3+ CD8+ TIL (**a**) and on CD3+ CD4+ TIL (**b**). **c-d** Percentage of OX40 expression on CD8+ T cells (**c**) and CD4+ T cells (**d**). Statistical significance between time points assessed was determined using a two-tailed, Wilcoxon matched pairs signed rank test. The number of samples analysed per time point is shown for each graph. Source data are provided in the Source Data file.

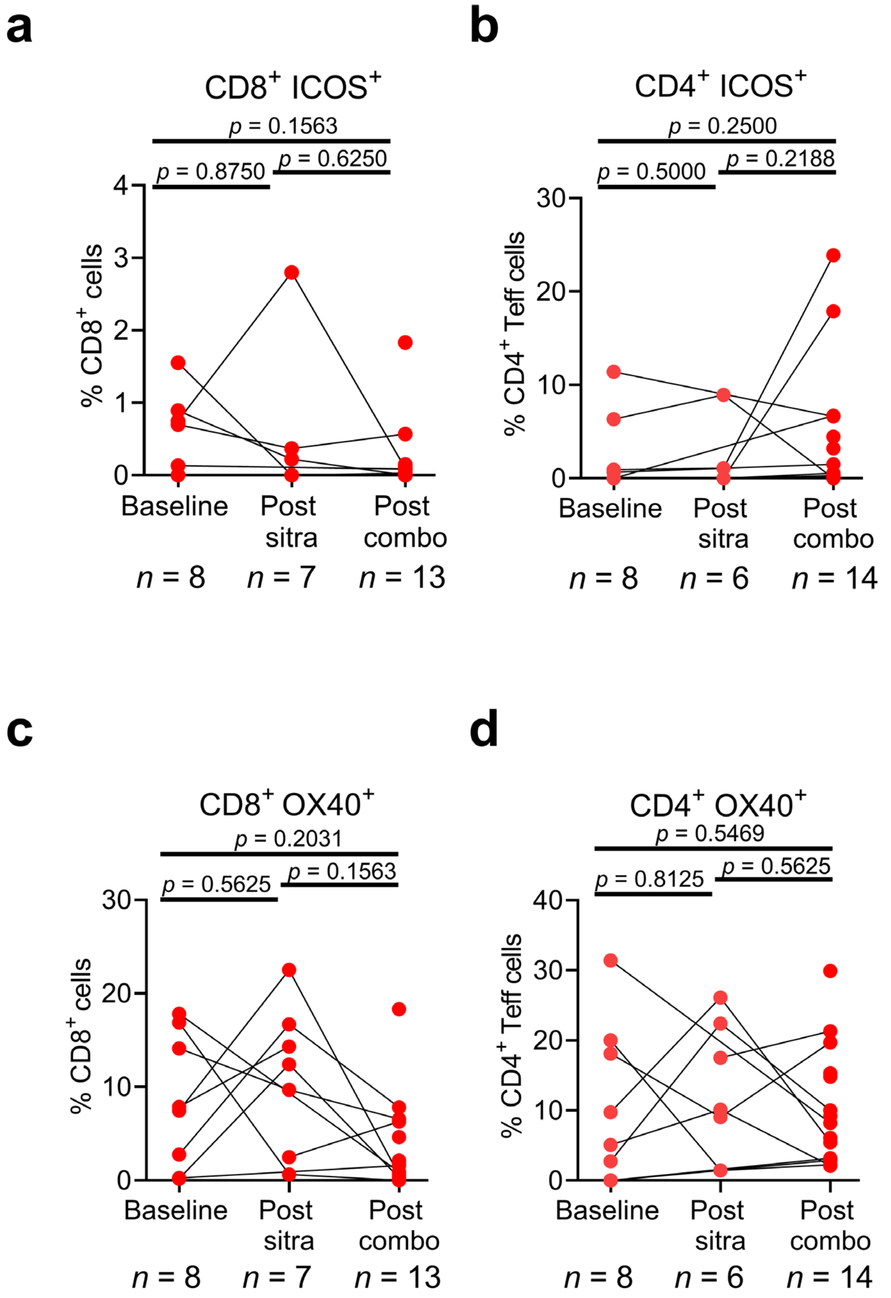

CD, cluster of differentiation; ICOS; inducible T-cell co-stimulator; TIL, tumour infiltrating lymphocytes.

Supplementary Figure S4. Modulation of immune activity in tumour tissue and circulation. a-d Flow cytometry analyses of tumour tissue (**a**) and PBMCs (**b-d**) collected longitudinally. The tumour tissues were collected at baseline, post-sitravatinib monotherapy, and at surgery post-combination therapy. The PBMCs were collected at baseline, after one day (Post1) and 15 days (Post2) of sitravatinib monotherapy, as well as after 14 days (Post3) and 29 days (Post4) of combination sitravatinib with nivolumab. The frequency of CD3+ T cells (left), CD8+PD-1+ T cells (middle) and CD4+PD-1+ T cells (right) is shown (**a**). The CD4/CD8 T cell ratio and frequency of each subset in the peripheral blood is shown over time (**b**). The percentage of PD-1 (left), Tim3 (middle) and Lag3 (right) expression on CD8+ T cells (**c**) and the percentage of the same receptors on the CD4+ T cells (**d**). Statistical significance between time points assessed was determined using a two-tailed, Wilcoxon matched pairs signed rank test. Exact P- values in **b-d** are shown if < 0.05. The number of samples analysed per time point is shown for each graph. Source data are provided in the Source Data file.

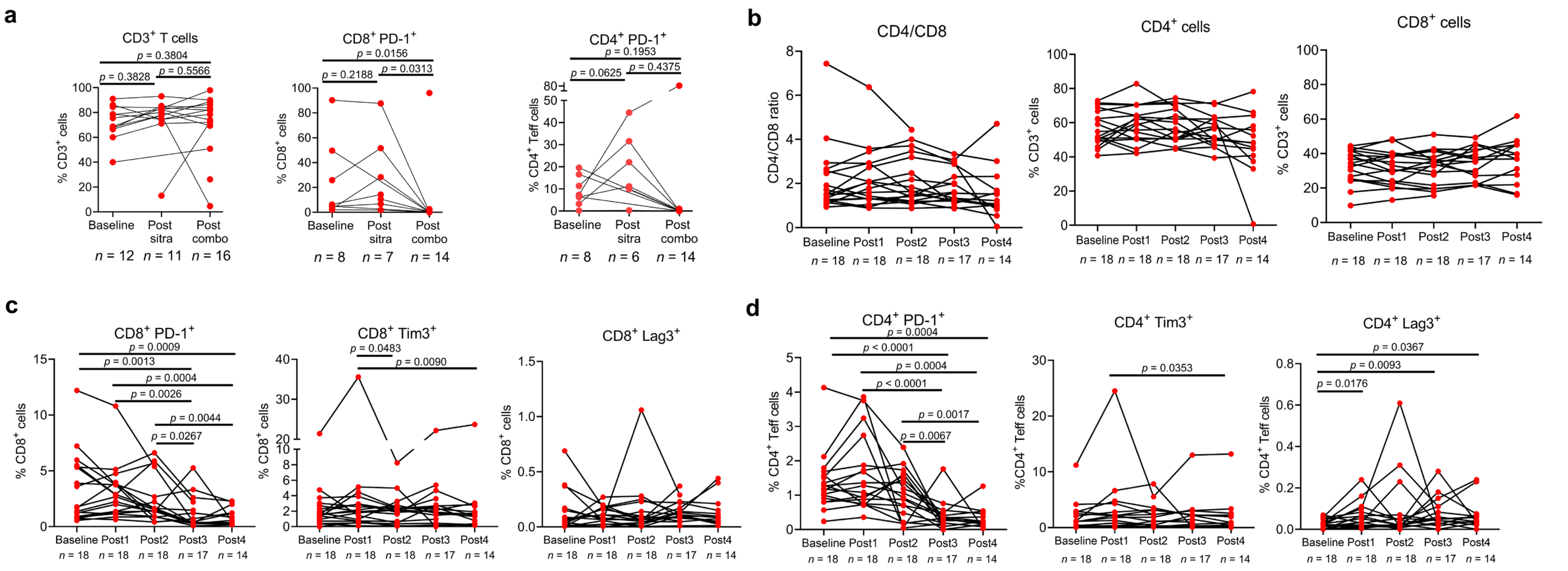

CD, cluster of differentiation; PD-1, programmed cell death-1; PMBC, peripheral blood mononuclear cell.

Supplementary Figure S5. a-c Changes in macrophages (**a**), M1:M2 ratio (**b**), and PD-L1+ macrophages (**c**) using multiplex immunofluorescence analysis on a subset of available tissue samples at baseline, post-sitravatinib, and post-combination therapy. **d** Representative image of M1 and M2 macrophage staining. Red, CD68; Green, CD163; Dark blue, HLA-DR; Light blue, CA9; Grey arrows, M1 macrophage (CD68+, CD163-, HLA-DR+); White arrows, M2 macrophage (CD68+, CD163+). The number of samples analysed per time point is shown for each graph along with the median cell counts, ratio and/or percentage per time point for each phenotype. Source data are provided in the Source Data file.

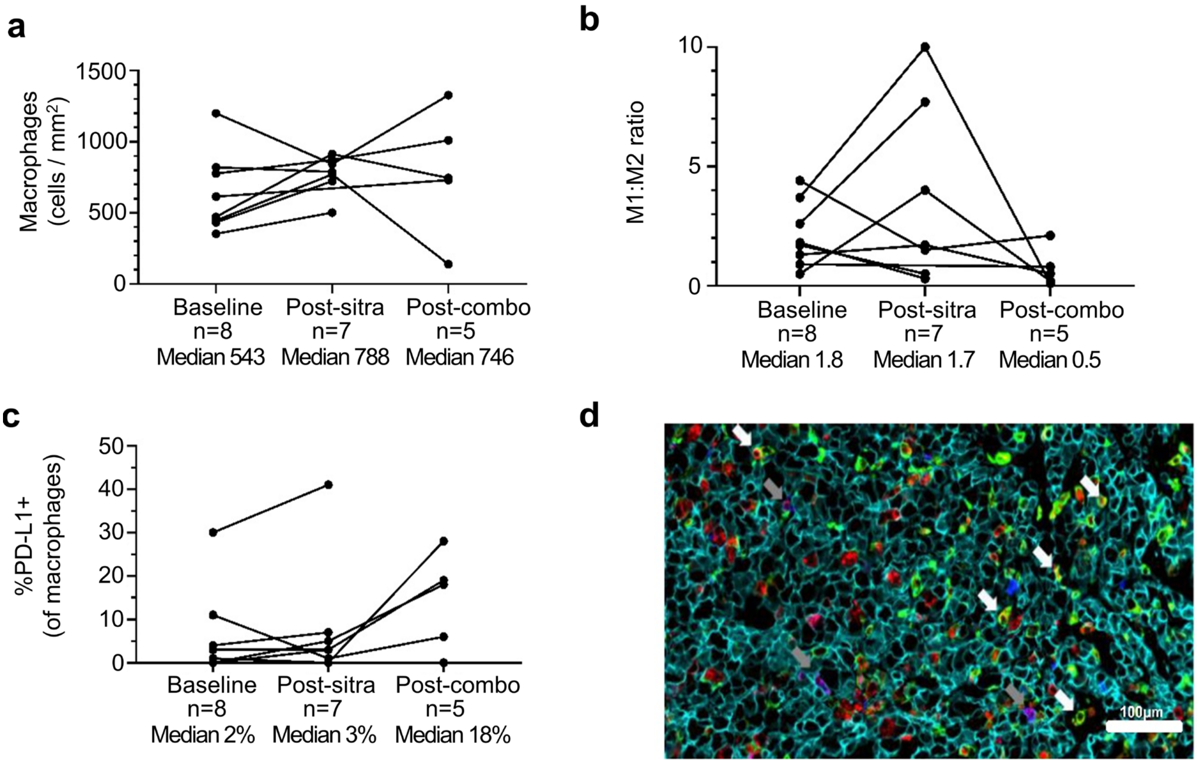

CD, cluster of differentiation; HLA-DR; human leukocyte antigen – DR isotype; PD-L1, programmed cell death ligand-1.

Supplementary Figure S6. a-c Changes in myeloid-derived suppressor cells using multiplex immunofluorescence analysis on a subset of available tissue samples at baseline, post-sitravatinib and post-combination therapy. Source data are provided in the Source Data file.

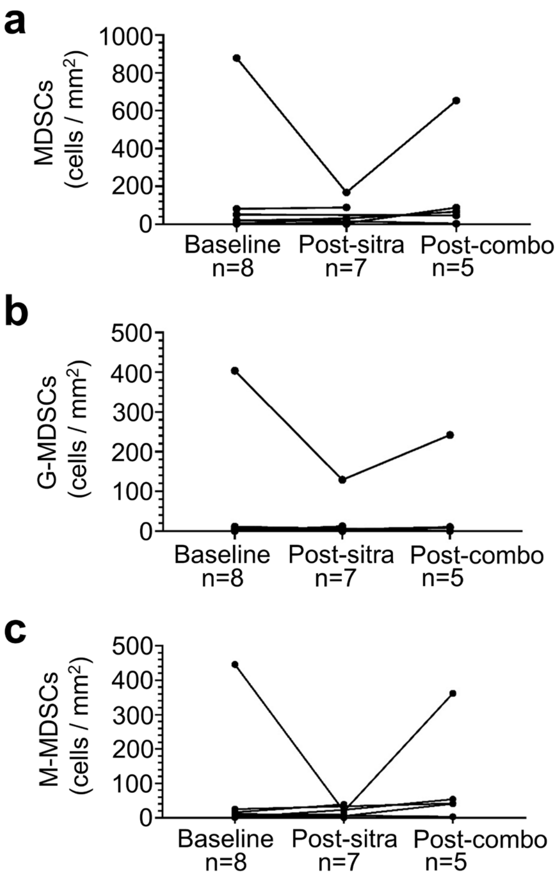

G-MDSC, granulocyte-MSDC; M-MDSC, monocytic-MSDC; MDSC, myeloid-derived suppressor cells.

Supplementary Figure S7. Flow cytometry gating strategy.

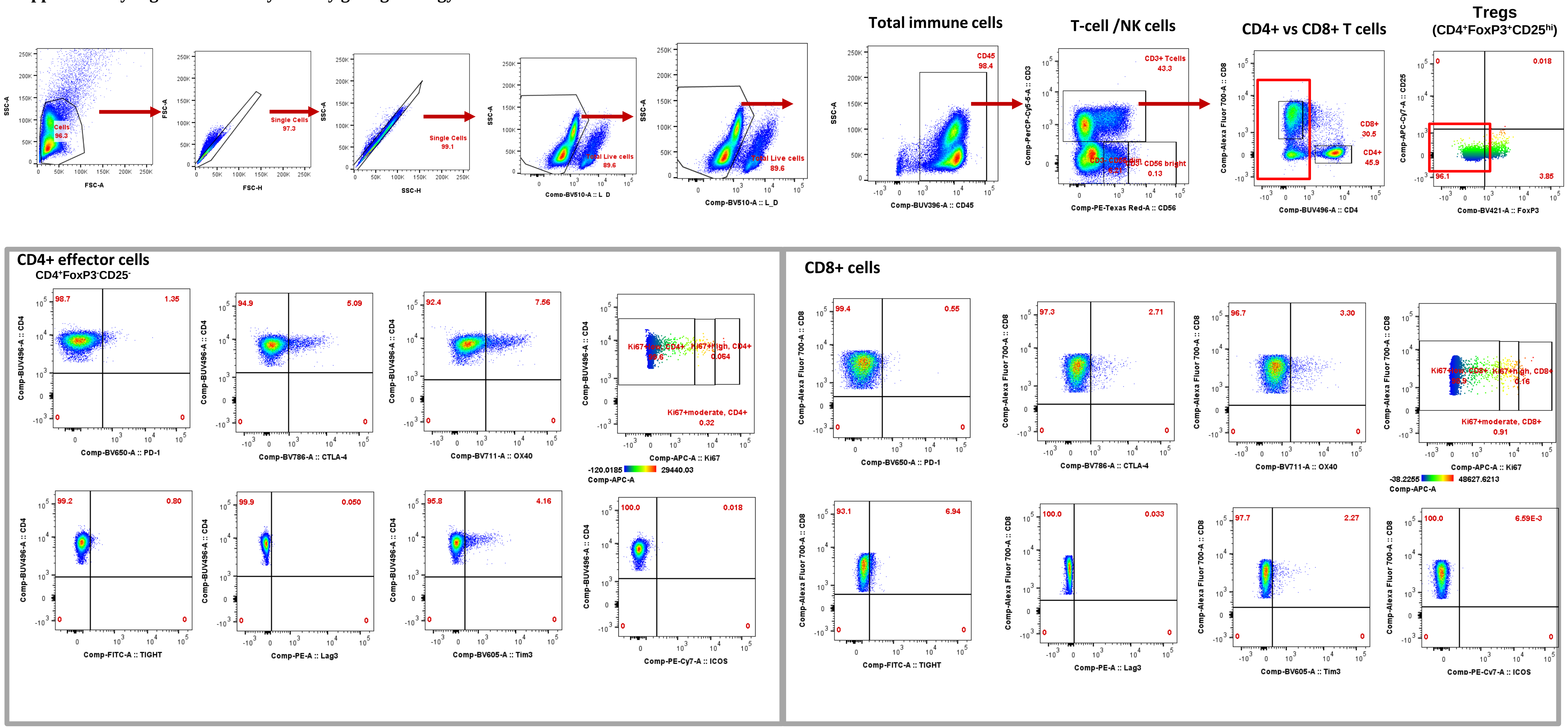

CD, cluster of differentiation

Supplementary Note

Clinical Trial Protocol

Protocol Number: 516-002

Official Title: A Phase 2 Study of Sitravatinib in Combination with Nivolumab in Patients Undergoing Nephrectomy for Locally-Advanced Clear Cell Renal Cell Carcinoma

NCT Number: NCT03680521

Document Date: 16-May-2018

CLINICAL RESEARCH PROTOCOL

**DRUG(S):**	Sitravatinib (MGCD516)<br>Nivolumab (OPDIVO®)
**STUDY NUMBER:**	516-002
**PROTOCOL TITLE:**	A Phase 2 Study of Sitravatinib in Combination with Nivolumab in Patients Undergoing Nephrectomy for Locally-Advanced Clear Cell Renal Cell Carcinoma
**IND NUMBER:**	138695
**SPONSOR:**	Mirati Therapeutics, Inc.<br>9393 Towne Centre Drive, Suite 200<br>San Diego, California, 92121, USA
**ORIGINAL PROTOCOL DATE:**	3 April 2018
**VERSION NUMBER:**	V3.0
**VERSION DATE:**	16 May 2018

CONFIDENTIALITY STATEMENT

This document contains confidential information and/or trade secrets of Mirati Therapeutics, Inc. As such, this document is protected from unauthorized disclosure or use by confidentiality agreement(s) and/or is exempt from public disclosure pursuant to applicable FDA and foreign counterpart regulations.

DOCUMENT HISTORY

Document	Version Date	Summary of Changes
Original Protocol, Version 1.0<br>(This version not submitted to FDA)	3 April 2018	NA
Amendment #1, Version 2.0<br>(This version submitted to FDA with the initial IND)	17 April 2018	• Added guidance regarding patient treatment and study discontinuation conditions for patients who develop adverse events that prevent subsequent nivolumab administration depending on treatment to date.<br>• Added details to sitravatinib dose modification to manage hematological and non-hematological adverse events, hypertension (added Table 5), decreased LVEF, and increase hepatic transaminases.<br>• Emphasized that OPDIVO Product Information must be consulted to ensure correct action is taken for nivolumab dose modification in the management of adverse events.<br>• Restricted secondary efficacy endpoint to Disease-Free Survival (DFS) and modified the frequency and method of collection of this endpoint.<br>• Addressed clerical errors and made minor clarifications.
Amendment #2, Version 3.0	16 May 2018	• In response to comments from the US Food and Drug Administration review, the following changes were made:<br>o Added a dose de-escalation plan for sitravatinib to monitor and limit toxicity of the combination regimen in the neoadjuvant setting using the Modified Toxicity Probability Interval (mTPI) method.<br>o Expanded guidance regarding sitravatinib discontinuation conditions.<br>o Added guidance regarding the need to undergo the scheduled surgery no later than 8 weeks following first dose of study drug, and not to extend the pre-operative period for missed doses of either study drug.<br>• Addressed clerical errors and made minor clarifications.

STUDY SUMMARY

Title: A Phase 2 Study of Sitravatinib in Combination with Nivolumab in Patients Undergoing Nephrectomy for Locally-Advanced Clear Cell Renal Cell Carcinoma

Rationale: Renal cell carcinomas (RCCs), which originate within the renal cortex, are responsible for 80% to 85% of all primary renal neoplasms. In the United States, RCC is the 6th most common cancer in men and the 9th most common cancer in women. An estimated 63,340 new cases and 14,970 deaths are expected in 2018. Incidence rates of RCC have been rising, with most of the increases occurring in early-stage tumors. Clear cell RCC (ccRCC) is the most common RCC histologic type, comprising 70-85% of all RCCs, and is overwhelmingly a nonhereditary (sporadic) cancer. At the time of diagnosis, approximately 25% of patients with ccRCC have locally-advanced disease. Locally-advanced tumors are managed with radical or partial nephrectomy with curative intent. Between 40% and 60% of such patients can be cured with such an aggressive surgical approach. However, given the relatively high rate of recurrence and the poor prognosis of metastatic ccRCC despite recent advances with immunotherapy and other targeted therapies, there remains an unmet medical need for patients with locally advanced RCC.

ccRCC is characterized by two important features. First, ccRCC is often associated with inactivating mutations of the von Hippel-Lindau (VHL) tumor suppressor gene, which leads to upregulation of hypoxia-inducible factors (HIF) and to downstream upregulation of vascular endothelial growth factor (VEGF), resulting in increased angiogenesis. VHL inactivation also leads to overexpression of receptor tyrosine kinases MET and AXL, known to be important in the pathogenesis and resistance to standard anti-angiogenic therapies for RCC. Second, it is often susceptible to immune regulation. These two features have led to the use of several active agents that either target the VEGF receptor (VEGFR) pathway (e.g., sunitinib, pazopanib) or modulate immune response (e.g., interferon-alpha [IFN-α], interleukin-2 [IL-2], nivolumab). Here, we postulate that the combination of sitravatinib, an inhibitor of VEGFR and other related kinases, and nivolumab, a Programmed Cell Death Protein 1 (PD-1) inhibitor, is not only active due to each component, but that the addition of sitravatinib can further enhance the immune effects from nivolumab.

Sitravatinib is a receptor tyrosine kinase inhibitor (TKI) that targets multiple closely related receptor tyrosine kinase pathways including

VEGFR, PDGF receptor (PDGFR), c-KIT, MET, and the TAM family of receptors (TYRO3, AXL, and MER). In an ongoing Phase 1/1b study of sitravatinib, clinical activity has been observed in patients with ccRCC, putatively due to sitravatinib's potent inhibition of VEGFR2 and inhibition of MET and AXL; which may mediate resistance to VEGFR inhibitors, and in patients with qualifying genetic alterations selected based on sitravatinib's target profile.

Nivolumab is a monoclonal antibody directed against PD-1 and blocks the interaction between PD-1 and its ligands, thereby releasing PD-1-mediated inhibition of T-cell proliferation (including cytotoxic CD8+ T-cells) and cytokine production. It has been evaluated in ccRCC after anti-angiogenic therapy in the Phase 3 CheckMate 025 trial, where patients treated with nivolumab at a dose level of 3 mg/kg every 2 weeks demonstrated a median OS of 25.0 months compared to 19.6 months for patients treated with everolimus, a mammalian target of rapamycin (mTOR) inhibitor, with a hazard ratio (HR) of 0.73 (98.5% CI: 0.57, 0.93; p=0.002). Nivolumab has been approved for use in this treatment setting.

Together, sitravatinib and nivolumab may cooperate to elicit greater anti-tumor activity than either agent alone, as sitravatinib is predicted to enhance several steps in the cancer immunity cycle that may augment the efficacy of nivolumab. First, the anti-tumor activity of sitravatinib may promote the release of tumor antigens. Second, inhibition of the split kinase receptors VEGFR and KIT may decrease the number of regulatory T-cells (Tregs) and myeloid-derived suppressor cells (MDSCs), thus promoting the expansion and migration of anti-tumor cytotoxic T-cells, and their infiltration into tumor tissue. Third, sitravatinib may reverse the immunosuppressive effects within the tumor microenvironment (TME) that are mediated by the TAM receptors through inhibition of MERTK (resulting in an increased number of the proinflammatory M1-polarized macrophages, proinflammatory cytokine release, and enhanced CD8+ T-cell activation), and through inhibition of AXL (predicted to increase the Toll-like receptor dependent inflammatory response in dendritic cells). Moreover, inhibition of MERTK and AXL may result in increased natural killer (NK) cell activity by releasing the negative feedback regulatory mechanism and may complement PD-1/PD-L1 checkpoint inhibition to augment the host anti-cancer immune response. Finally, inhibition of MET may enhance the anti-tumor response by restoring antigen-presenting cell (APC) function and reducing or eliminating MDSCs within the TME.

Thus, the combination of sitravatinib and nivolumab is a rational approach for patients with ccRCC with effects at several steps in the immunity cycle. This combination has been shown to be safe and tolerable in an ongoing Phase 2 Study (MRTX-500) in second-line patients with metastatic non-small cell lung cancer (NSCLC). The recommended Phase 2 dose for this combination is 120 mg orally daily of sitravatinib and 240 mg of nivolumab (flat dose) administered IV Q2W.

Neoadjuvant, or preoperative, therapy can lead to primary tumor downsizing or downstaging, which may decrease intra-operative and peri-operative morbidity and potentially allow for less extensive surgery or less invasive approaches. In addition, in locally-advanced RCC, neoadjuvant therapy can potentially control micrometastatic disease present at the time of surgery. Neoadjuvant targeted therapies have been investigated in several Phase 2 trials in both locally-advanced and metastatic ccRCC and have demonstrated reduction of primary tumor burden prior to surgery. Neoadjuvant axitinib, a VEGFR TKI, administered for 12 weeks is clinically active and reasonably well tolerated in the neoadjuvant setting in patients with locally-advanced ccRCC (28% median reduction of primary renal tumor diameter, with all patients experiencing tumor reduction and with a partial response [PR] rate of 46%). Similarly, neoadjuvant sunitinib, a VEGFR and PDGFR TKI, administered for 8 weeks is safe and feasible in the neoadjuvant setting in patients with either locally-advanced or metastatic ccRCC, and active, demonstrating a 21% median reduction of primary renal tumor diameter, with all patients experiencing tumor reduction and 29% achieving a PR.

In summary, in addition to sitravatinib's potent inhibition of VEGFR and inhibition of MET and AXL (which potentially mediate resistance to VEGFR inhibitors), sitravatinib may act synergistically with immune checkpoint inhibitors such as nivolumab in immune activation in ccRCC. Treatment of ccRCC with sitravatinib may produce rapid tumor cell death and release of tumor associated antigens as well as enhance tumor antigen presentation, inhibit immunosuppressive cell types and prime and activate anti-tumor T-cell responses. Here, we evaluate the clinical activity of this combination as neoadjuvant treatment of ccRCC and assess correlative immune effects.

Target Population: Patients with locally-advanced clear cell renal cell carcinoma (ccRCC) who are candidates for nephrectomy.

Number in Primary Trial: Approximately 25 patients with locally-advanced RCC who are candidates for nephrectomy in order to accrue 18 clinical activity evaluable patients with clear cell histology.

Primary Objective:

- To evaluate the preoperative clinical activity of the combination of sitravatinib and nivolumab in patients with locally-advanced ccRCC undergoing nephrectomy.

Secondary Objectives:

- To evaluate the safety and tolerability of the combination regimen in the selected population.
- To characterize the baseline tumor-related immune profile in patients with RCC.
- To determine the immune effects of sitravatinib and the combination regimen in patients with ccRCC.
- To evaluate the pharmacokinetics (PK) of sitravatinib administered daily alone and in combination with nivolumab in patients with ccRCC.
- To characterize the time to surgery.
- To evaluate secondary efficacy endpoints with the combination regimen in the selected population.

Exploratory Objective:

- To evaluate changes in gene expression and the T-cell repertoire in response to therapy in patients with ccRCC.

Primary Endpoint:

- Percentage of patients achieving a point in time objective response (either complete or partial response [CR or PR]) prior to surgery.

Secondary Endpoints:

- Safety characterized by type, incidence, severity, timing, seriousness and relationship to study treatment of adverse event (AEs) and laboratory abnormalities.
- Descriptive characterization of immune cell populations in the tumor and/or peripheral blood at baseline.
- Temporal changes in PD-L1 expression, selected cytokines and immune cell populations in the tumor and/or peripheral blood (including myeloid-derived suppressor cells [MDSCs], regulatory T-cells [Tregs], $CD4^+$ [helper] and $CD8^+$ [cytotoxic] T-cells, and the ratio of Type1:Type 2 tumor-associated macrophages).

- Blood plasma concentration of sitravatinib.
- Time-to-surgery.
- Disease Free-Survival (DFS).

Exploratory Endpoint:

- Gene expression and T-cell receptor sequencing.

Study Design:

This study (516-002) is an open-label, non-randomized, preoperative window of opportunity Phase 2 study of sitravatinib and nivolumab in the neoadjuvant setting for the treatment of patients with locally-advanced clear cell renal cell carcinoma (ccRCC) undergoing nephrectomy. Approximately 25 patients with a clinical diagnosis of locally-advanced renal cancer will be enrolled into the study in order to identify a total of 18 clinical activity evaluable patients with clear cell histology and detect the primary endpoint of percentage of patients with ccRCC achieving a point in time objective response (either CR or PR) prior to surgery with the combination treatment.

Prior to Start of Treatment: All enrolled patients will have met all entry criteria and have completed baseline procedures and will be scheduled to undergo an initial diagnostic tumor biopsy of their renal lesion. Patients with a histology other than ccRCC will proceed to their End of Study (EOS) visit following the histology results, with no treatment with study drugs and no follow-up.

Subjects with a clear cell histology will proceed to the study combination treatment which will be conducted in 2 sequential preoperative treatment segments.

Segment 1 (2 weeks) consists of a 2-week treatment with single-agent sitravatinib. Sitravatinib will be administered at a dose of 120 mg orally once daily (QD), on a continuous daily dosing regimen. After completion of the 2-week dosing period, patients will undergo a renal biopsy.

Segment 2 (4-6 weeks) consist of combination treatment with sitravatinib and nivolumab for at least 4 weeks (and up to a maximum of 6 weeks to allow for flexibility with the scheduling of the nephrectomy). Sitravatinib will be administered at a dose of 120 mg orally QD, on a continuous daily dosing regimen for this entire segment. Nivolumab will be administered, in accordance with approved labeling, by intravenous (IV) infusion, 240 mg Q2W (namely, on D15, D29, and potentially D43). Patients will receive

the nivolumab injection on D43 only if their surgery is expected to occur more than a week from that date. Patients will be subject to a 2-day preoperative hold of all study drugs prior to surgery (ensuring that the last dose of any study drug is administered a minimum of 72 hours prior to surgery).**Surgery:** After completion of neoadjuvant therapy and the ~2-day pre-operative period, patients will undergo nephrectomy. Patients will have their End of Study (EOS) visit following surgery, with no further study drug administration. Patients will be followed for disease-free survival for up to 3 years or more until either death, disease recurrence or loss to follow-up. Of note, patients should be scheduled to undergo surgery no later than 8 weeks following their first dose of study drug, and the preoperative period (including Segments 1 and 2) should not be extended in order to take missed doses of either study drug.

Figure 1: Study Schema

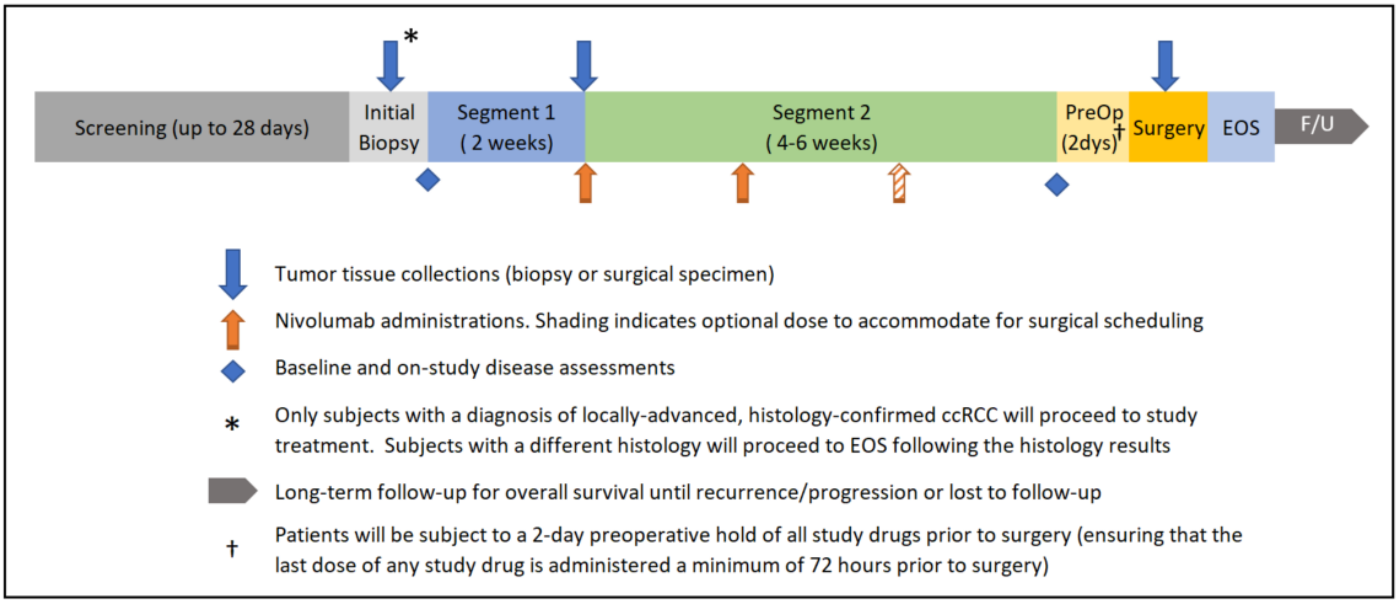

Statistical Considerations: The primary endpoint of this study is percentage of patients with ccRCC achieving a point in time objective response (either CR or PR) prior to surgery with the combination treatment of sitravatinib and nivolumab. Objective responses will be assessed in accordance with RECIST 1.1. With currently available treatments, the percentage of patients with a point in time objective response prior to surgery is assumed to be 5% (p0); thus, this rate is considered uninteresting. The target percentage of patients with a point in time objective response prior to surgery using sitravatinib and nivolumab in this study is assumed to be 30% (p1). Controlling for a Type 1 error (α) of 0.05, and using an exact test (two-sided), with 18 clinical activity evaluable patients, we have 80% power to rule out a

percentage of patients with a point in time objective response prior to surgery of 5% assuming percentage of patients with a point in time objective response prior to surgery of 30%. Additional subjects may be enrolled for a total of 12 subjects with complete tumor tissue collection (collected from initial and mid-study biopsies and surgical sample).

In addition, a plan for possible dose de-escalation will be implemented. Decisions regarding dose de-escalation will be made using the Modified Toxicity Probability Interval (mTPI) method to set rules on dose de-escalation in the first 6 patients and then on an ongoing basis. Assuming a maximum toxicity level of no more than 20% at the tolerated dose, the dose of sitravatinib will be decreased if more than 2 patients with toxicity are observed in the first 6 patients. Specific treatment-related adverse events will be used to determine the number of patients with toxicity.

Table 1 Schedule of Assessments

Study Assessments	Screening<br>D-28 to Initial Biopsy	Initial Biopsy[1]<br>D-4 (±3)	Neoadjuvant Tx (6-8[2] wks)<br>D1	D14[3] (±1)	D15 (±2)	D29 (±2)	D43[2] (±2)	Preop Rebaseline<br>≤3 dys of Surgery	Surgery	EOS/ Withdrawal[4]	Long-term Follow-Up
Informed Consent[5]	X										
Collection of Tumor Tissue[6]		X		X					X		
Medical History, Disease History, Prior Therapy	X										
Physical Exam[7] including Vital Signs & ECOG PS	X									X	
Abbreviated Physical Exam[8] including Vital Signs			X		X	X	(X)	X			
Hematology and Serum Chemistry[9,10]	X	X	X		X	X	(X)	X		X	
Coagulation and Urinalysis[9,10]	X		As clinically indicated					Coagulation only	As clinically indicated		
Thyroid Function Test[9,10]	X							X		X	
Pharmacokinetic, Pharmacodynamic, and Baseline ctDNA Blood Samples, and Triplicate 12-Lead ECG[11]	See Table 2		See Table 2								
Single 12-Lead ECG[11]	X	As clinically indicated								X	
Echocardiogram	X					X		X			
Disease Assessment[12]	X							X			
Pregnancy Test[13]	X	As clinically indicated									
Sitravatinib Dispensing and/or Reconciliation			X			X		Preop hold for 2 dys from Surgery[16]	Surgery Hold		
Nivolumab Administration					X	X	(X)				
AEs[14] and Concomitant Medications	SAEs only	Throughout									
Long-Term Follow-Up[15]											X

The Schedule of Assessments provides an overview of the protocol visits and procedures. Refer to Section 7 for detailed information on each assessment. Additional, unplanned assessments should be performed as clinically indicated, including for the purpose of fully evaluating AEs (adverse events).

1 On the Initial Biopsy Visit, laboratory blood assessments completed in the previous week do not need to be repeated. The Initial Biopsy will preferably be scheduled for D-4 (±3); however, to allow flexibility for logistical reasons, this period can include up to 2 weeks from D1 (including D-14).

2 Neoadjuvant therapy will be administered for at least 6 weeks (unless treatment discontinuation is warranted), and up to a maximum of 8 weeks, to allow for flexibility with the scheduling of the nephrectomy (surgery). Consequently, nivolumab will be administered as neoadjuvant therapy for at least 2 injections, and up to a maximum of 3 injections. The third injection will only be administered if the subject's planned day of Surgery is at least one week after D43 and further treatment is not contraindicated [e.g., interruptions for adverse events (AEs)]. Assessments that occur in the 2 weeks that are meant to allow flexibility for the scheduling of the surgery are indicated in ( ).

3 D14 visit (±1), which has for purpose the mid-study biopsy, must occur prior to first nivolumab injection on D15 (±2).

4 End of Study (EOS)/Withdrawal: Laboratory blood assessments completed in the previous 4 weeks do not need to be repeated. EOS visit must occur at least 28 days after last dose of study drug or surgery, whichever occurs last.

5 Informed Consent: May be performed within 28 days prior to the first dose of sitravatinib therapy on D1 and must be completed prior to initiation of any study specific assessments.

6 Collection of Tumor Tissue: Tumor tissue will be collected from initial and mid-study biopsies and surgical sample. Fresh tumor biopsies are to be collected on study; archival tissue will not be accepted as an alternative. The tumor biopsy collected on the Initial Biopsy Visit will confirm the histology of the renal cancer. Tumor tissue will be collected and used to assess immune effects through tissue biomarkers prior to and during and following treatment. Parameters may include, but are not limited to, quantification of tissue immune cell populations by flow cytometry including T-cell subpopulations (CD8+, CD8+/Ki67+ and regulatory T-cells [Tregs]), natural killer (NK) cells, myeloid-derived suppressor cells (MDSCs) and macrophages; immunohistochemistry and/or immunofluorescence for assessment of PD L1 status and characterization of immune cell populations; tumor gene expression analysis; and DNA sequencing to assess T-cell clonality. In addition, next generation sequencing (NGS) analyses for tumor mutations and tumor mutation burden may be assessed using tissue from the surgical sample.

7 Physical Examinations: A complete physical examination required at Screening and EOS/Withdrawal only. Height will be recorded at screening only. All other evaluations will be symptom-directed, abbreviated evaluations. Vital signs include weight, temperature, blood pressure, and pulse rate to be assessed prior to dosing as indicated.

8 Abbreviated Physical Exam includes vital signs (weight, temperature, blood pressure, and pulse rate to be assessed prior to dosing as indicated) and symptom-directed evaluation.

9 Selected D1 Assessments: Repeat assessment not required if screening assessment performed within 7 days before the first dose.

10 Safety Laboratory Assessments: Hematology, coagulation, chemistry, thyroid function and urinalysis evaluations (see Table 7) will be performed by local laboratories.

11 Pharmacokinetic (PK) Blood Samples: Blood samples for PK measurements will be collected as outlined in Table 2.
Pharmacodynamic Blood Samples: Blood samples will be collected for the assessment of immune effects through circulating biomarkers prior to and during and following treatment as outlined in Table 2. Parameters may include, but are not limited to, quantification of circulating immune cell populations by flow cytometry including T-cell subpopulations (CD4+, CD4+/Ki67+, CD8+, CD8+/Ki67+ and Tregs), NK cells, and MDSCs; blood levels of selected cytokines and chemokines including IFN-γ, IL-1ß, IL-6, IL-8, IL-12, IL-18 and CXCL10 (IP-10); and DNA sequencing to assess T-cell clonality.
Baseline ctDNA Blood Samples: NGS analyses for tumor mutations and tumor mutation burden may be assessed at baseline using ctDNA as outlined in Table 2.
12-Lead ECGs: Triplicate ECGs will accompany PK sampling as described in Table 2. In addition, single ECGs are to be performed as clinically indicated. Assessments will include an evaluation of RR, QT, and QTc intervals. In case RR interval cannot be machine-read, ventricular heart rate should be reported instead.

12 Disease Assessments will occur at the following times:

a) during screening (within 28 days of D1),
b) preoperatively (within 1 week prior to the surgery).

At screening/baseline, assessments are to include evaluation of any superficial lesions, Computed Tomography Scan (CT) with contrast or X-ray (radiography) of the chest, Magnetic Resonance Imaging (MRI) with and without gadolinium or CT abdomen, as well as, if clinically indicated, MRI of the brain and a chest x-ray. The subsequent disease assessment should include all sites of disease identified at baseline or suspected to have developed. More detailed guidance on exceptional circumstances is provided in the protocol.

13 Pregnancy Test: If the patient is a woman of childbearing potential, negative serum or urine pregnancy test performed by the local laboratory at screening will be required. The informed consent process must include discussion of the risks associated with pregnancy and adequate contraception methods. Additional pregnancy testing may be necessary if required by local practices or regulations, or if potential pregnancy is suspected.

14 Adverse Events: SAEs (serious adverse events) will be reported from the time of informed consent until at least 28 days after the last administration of sitravatinib or nivolumab. Ongoing SAEs should be followed until resolution or stabilization to a chronic condition. AEs will be reported from the day of the initial biopsy until at least 28 days after last dose of study drug.

15 Long Term Follow-up: Survival status will be collected by review of medical records or telephone contact with the patient or treating healthcare professional approximately every 6 months from EOS visit for up to 3 years or more until death, disease recurrence or loss to follow-up. Disease recurrence will be based on imaging assessments performed off study per standard of care after nephrectomy.

16 2-day preoperative hold such that the last dose of any drug is administered a minimum of 72 hours prior to surgery.

Table 2: Schedule of Pharmacokinetic, Pharmacodynamic, Baseline ctDNA, and Triplicate ECG Assessments

	**Screening**	**Neoadjuvant Tx**						**Preop Rebaseline**
	D-28 to Initial Biopsy	**D1**			**D15 (± 2)**	**D29 (± 2)**	**D43 (± 2)**	≤ 3 dys of Surgery
**Collection Time and Allowable Window**		**Pre-dose (-0.5-0 hour)**	**30 min (± 10 min)**	**4 hour (2-6 hour)**	**Pre-dose (-2-0 hour)**	**Pre-dose (-2-0 hour)**	**Pre-dose (-2-0 hour)**	
PK Blood Samples[1,2,3]		X	X	X	X		X (whichever visit occurs first)	
Flow Cytometry Blood Samples[3,4]	X >2 dys from D1	X			X	X	X (whichever visit occurs first)	
Protein and Cytokine Biomarkers Blood Samples[3,4]		X			X	X	X (whichever visit occurs first)	
TCR Sequencing Blood Samples[3,4]		X						X
Baseline ctDNA Blood Samples[4,5]	X							
Triplicate ECG[6]		X	X		X			

1 Unscheduled PK blood samples should be drawn in association with two kinds of safety events: 1) as soon as possible after a Serious Adverse Event (SAE), and 2) at a clinic visit at least one week following a dose modification of sitravatinib.
2 Scheduled vital signs and triplicate ECGs precede PK sample collection in all cases. Sitravatinib dosing and sampling should precede nivolumab infusion.
3 Pharmacokinetic, pharmacodynamic, and baseline ctDNA blood samples should be collected as detailed in the study Lab Manual and in the study kit.
4 Blood for Pharmacodynamics: Immune effects will be evaluated through circulating prior to and during and following treatment. Blood samples will be collected for the assessment of parameters that may include, but are not limited to, quantification of circulating immune cell populations by flow cytometry including T-cell subpopulations (CD4+, CD4+/Ki67+, CD8+, CD8+/Ki67+ and Tregs), NK cells, and MDSCs; blood levels of selected cytokines and chemokines including IFN-γ, IL-1ß, IL-6, IL-8, IL-12, IL-18 and CXCL10 (IP-10); and DNA sequencing to assess T-cell clonality.
5 Baseline ctDNA Blood Samples: NGS analyses for tumor mutations and tumor mutation burden may be assessed at baseline using ctDNA.
6 ECGs should be taken in triplicate, each reading approximately 2 minutes apart. On D1 only, two sets of triplicate ECGs should be done within 1 hour prior to dosing (e.g., at 30-minute intervals) to firmly establish the baseline for the patient. In general, ECGs should be performed prior (within -30 to -5 minutes) to the respective PK blood collection.
   - Example for D1 pre-dose ECG/PK assessments: ~ -1.0 hour (Triplicate ECGs); ~ -30 minutes (Triplicate ECGs); ~ -15 minutes (Vitals/PK).
   - Example for all other pre-dose ECG/PK assessments: ~ -30 minutes (Triplicate ECGs); ~ -15 minutes (Vitals/PK).

   Assessments will include evaluation of RR, QT and QTc intervals. In case RR interval cannot be machine-read, ventricular heart rate should be reported instead.

TABLE OF CONTENTS

CLINICAL RESEARCH PROTOCOL 1
DOCUMENT HISTORY 2
STUDY SUMMARY....................3
TABLE OF CONTENTS 13
TABLE OF TABLES....................16
TABLE OF FIGURES....................16
LIST OF ABBREVIATIONS 18
LIST OF ABBREVIATIONS (CONTINUED) 19
LIST OF ABBREVIATIONS (CONTINUED) 20
LIST OF ABBREVIATIONS (CONTINUED) 21
LIST OF ABBREVIATIONS (CONTINUED) 22
1 INTRODUCTION AND RATIONALE 23
1.1 Background 23
1.1.1 Renal Cell Carcinoma....................23
1.1.2 Sitravatinib 23
1.1.3 Nivolumab....................23
1.1.4 Potentially Synergistic Effects 24
1.1.5 Neoadjuvant Setting in Locally-Advanced ccRCC....................25
1.2 Sitravatinib Background....................27
1.2.1 Sitravatinib Drug Substance....................27
1.2.2 Sitravatinib Non-Clinical Data 28
1.2.3 Sitravatinib Clinical Data....................29
1.2.3.1 Sitravatinib Pharmacokinetics 29
1.2.3.2 Sitravatinib Clinical Safety 29
1.2.3.3 Sitravatinib Clinical Efficacy....................31
1.3 Nivolumab Background 31
1.3.1 Nivolumab Drug Substance 31
1.3.2 Nivolumab Non-Clinical Data....................31
1.3.3 Nivolumab Clinical Data 31
1.3.3.1 Nivolumab Pharmacokinetics....................32
1.3.3.2 Nivolumab Anti-Drug Antibodies 32
1.3.3.3 Nivolumab Adverse Reactions Common in Clinical Trials 32
1.3.3.4 Nivolumab Immune-Related Adverse Events 33
1.4 Combination Safety of Sitravatinib and Nivolumab 33
1.4.1 Potential for Drug-Drug Interactions 33
1.4.2 Evaluation of Potential for Increased Toxicities with Combination Use of Sitravatinib and Nivolumab 34
2 STUDY OBJECTIVES 35
2.1 Objectives....................35
2.1.1 Primary Objective....................35
2.1.2 Secondary Objectives....................35
2.1.3 Exploratory Objective 35
2.2 Endpoints....................36
2.2.1 Primary Endpoint....................36

2.2.2 Secondary Endpoints 36
2.2.3 Exploratory Endpoints 36
3 STUDY DESIGN 36
4 SUBJECT SELECTION AND ENROLLMENT 38
4.1 Inclusion Criteria 38
4.2 Exclusion Criteria 39
4.3 Life Style Guidelines 41
4.4 Enrollment into Study 41
5 STUDY TREATMENT 42
5.1 Sitravatinib Study Drug 42
5.1.1 Sitravatinib Formulation, Packaging and Storage 42
5.1.2 Sitravatinib Preparation, Dispensing, Administration and Accountability 42
5.1.3 Sitravatinib Dose Modification and Discontinuation 43
5.2 Nivolumab Study Drug 44
5.2.1 Formulation and Packaging 44
5.2.2 Preparation and Dispensing 44
5.2.3 Administration 45
5.2.4 Dose Modification 45
5.3 Management of Adverse Events 45
5.3.1 Sitravatinib-Related Adverse Events 46
5.3.1.1 General Management of Non-Hematological Toxicities 46
5.3.1.2 General Management of Hematological Toxicities 47
5.3.1.3 Management of Selected Adverse Events 47
5.3.2 Nivolumab-Related Adverse Events 49
5.3.3 Management in Event of Immune-Related Adverse Events 50
5.3.3.1 Diarrhea/Colitis 50
5.3.3.2 Increased Transaminases 50
5.3.3.3 Management of Hy's Law Cases 51
5.4 Medication Error 51
5.5 Concomitant Therapies 51
5.5.1 Concomitant Medication(s) 51
5.5.2 Concomitant Surgery or Radiation Therapy 53
5.5.3 Other Anticancer or Experimental Therapy 53
6 STUDY ASSESSMENTS 53
6.1 Screening 53
6.2 Study Period 53
6.3 End of Study (EOS) Assessment 53
6.4 Long-Term Follow-up 54
6.5 Patient Discontinuation/Withdrawal 54
7 PROCEDURES 54
7.1 Efficacy 55
7.2 Safety Assessments 56
7.2.1 Medical History 56
7.2.2 Physical Examination and Vital Signs 56
7.2.3 Laboratory Safety Assessments 56

7.2.4 Electrocardiogram (ECG)....57
7.2.5 Echocardiogram (ECHO)....57
7.3 Laboratory Studies....57
7.3.1 Pharmacokinetic Evaluation....58
7.3.2 Pharmacodynamic Evaluation in Blood....58
7.3.3 Pharmacodynamic Evaluation in Tumor Tissue....58
7.3.4 Circulating and Tissue Tumor DNA....59
8 ADVERSE EVENT REPORTING....59
8.1 Sponsor Medical Monitor Personnel....59
8.2 Adverse Events....59
8.2.1 Laboratory Abnormalities....60
8.2.1.1 Hy's Law....60
8.2.2 Severity Assessment....61
8.2.3 Causality....61
8.3 Serious Adverse Events....61
8.3.1 Definition of a Serious Adverse Event....61
8.3.2 Exposure During Pregnancy....63
8.4 Reporting of Serious Adverse Events and Adverse Events....63
8.4.1 Reporting Period....63
8.4.2 Reporting Requirements....64
9 STATISTICS....64
9.1 Statistical Design....65
9.1.1 Hypothesis and Sample Size....65
9.1.2 Dose De-escalation Plan....65
9.2 Data Handling....66
9.3 Analysis Populations....66
9.3.1 Clinical Activity Evaluable Population....66
9.3.2 Safety Population....66
9.3.3 Pharmacokinetic Evaluable Population....66
9.3.4 Pharmacodynamic Evaluable Population....66
9.4 Efficacy Endpoint Definitions and Analyses....67
9.4.1 Percentage of Patients Achieving a Point in Time Objective Response (either CR or PR) Prior to Surgery....67
9.4.2 Disease-Free Survival (DFS)....67
9.5 Safety Data Presentations and Summaries....68
9.5.1 Prior and Concomitant Medications....68
9.5.2 Adverse Events....68
9.5.3 Clinical and Laboratory Assessments....68
9.5.4 Patient Demographics, Baseline Characteristics and Disposition....68
9.5.5 Analysis of Study Treatment Dosing....69
9.5.6 Time-to-Surgery....69
9.6 Other Study Endpoints....69
9.6.1 Pharmacokinetic Analysis....69
9.6.2 Pharmacodynamic and Exploratory Analysis....69
9.7 Interim Analysis....69
9.8 Data Monitoring Committee....69

10 ETHICS AND RESPONSIBILITIES 70
10.1 Ethical Conduct of the Study 70
10.2 Obligations of Investigators 70
10.3 Institutional Review Board/Ethics Committee/Research Ethics Board 70
10.4 Informed Consent Form 71
10.5 Confidentiality 71
10.6 Reporting of Serious Breaches of the Protocol or ICH GCP 71
11 RECORDS MANAGEMENT 72
11.1 Source Documentation 72
11.2 Study Files and Records Retention 72
12 QUALITY CONTROL AND QUALITY ASSURANCE 72
12.1 Monitoring Procedures 72
12.2 Auditing and Inspection Procedures 72
13 CHANGES IN STUDY CONDUCT 73
13.1 Protocol Amendments 73
13.2 Protocol Deviations 73
14 END OF TRIAL 73
14.1 End of Trial in a European Union Member State 73
14.2 End of Trial in all other Participating Countries 74
14.3 Premature Termination 74
15 STUDY REPORT AND PUBLICATION POLICY 74
16 REFERENCES 75
Appendix 1. ECOG PERFORMANCE STATUS 80
Appendix 2. MEDICATIONS TO BE AVOIDED OR USED WITH CAUTION DURING STUDY TREATMENT 81
Appendix 3. ABBREVIATED PRESENTATION OF RECIST VERSION 1.1 GUIDELINES 83
Appendix 4. DOSE DE-ESCALATION DECISION TABLE USING the mtpi method 88

TABLE OF TABLES

Table 1 Schedule of Assessments 10
Table 2: Schedule of Pharmacokinetic, Pharmacodynamic, Baseline ctDNA, and Triplicate ECG Assessments 12
Table 3: Sitravatinib Sequential Dose Reductions for Individual Patients 44
Table 4: Sitravatinib Dose Modifications – Non-Hematological Drug-Related Toxicities[1] 46
Table 5: Sitravatinib Dose Modification for Increased Blood Pressure 48
Table 6: Sitravatinib Dose Modification for Increased Hepatic Transaminase 51
Table 7: Laboratory Safety Parameters 56

TABLE OF FIGURES

Figure 1: Study Schema 8

LIST OF ABBREVIATIONS

AE	Adverse Event
AESI	Adverse Event of Special Interest
ALT	Alanine Aminotransferase
APC	Antigen-Presenting Cell
ASCO	American Society of Clinical Oncology
AST	Aspartate Aminotransferase
AUC	Area Under the Plasma Concentration
AUC_{0-24}	Area Under the Plasma Concentration Versus Time Curve from Time 0 to 24 Hours
$AUC_{0-24,ss}$	Area Under the Plasma Concentration Versus Time Curve from Time 0 to 24 Hours, Steady State
BCRP	Breast Cancer Resistance Protein
BP	Blood Pressure
BUN	Blood Urea Nitrogen
$C_{av,ss}$	Average Steady-State Plasma Drug Concentration During Multiple-Dose Administration
ccRCC	Clear Cell Renal Cell Carcinoma
CD4+/8+	Cluster of Differentiation 4-Positive/8-Positive
CFR	Code of Federal Regulations
CHF	Congestive Heart Failure
CIT	Checkpoint Inhibitor Therapy
C_{max}	Maximum Plasma Concentration
CO_2	Carbon Dioxide
CR	Complete Response
CrCl	Creatinine Clearance
CRF	Case Report Form
CRO	Contract Research Organization
CT	Computed Tomography
CTA	Clinical Trial Application
CTCAE	Common Terminology Criteria for Adverse Events
ctDNA	Circulating Tumor Cell DNA
CV%	% Coefficient of Variation
CXCL	Chemokine (C-X-C) Ligand
CYP	Cytochrome P450
DDI	Drug-Drug Interaction

LIST OF ABBREVIATIONS (CONTINUED)

DDR2	Discoidin Domain Receptor 2
DFS	Disease-Free Survival
DLT	Dose-Limiting Toxicity
DNA	Deoxyribonucleic Acid
dys	Days
EC	Ethics Committee
ECG	Electrocardiogram
ECHO	Echocardiogram
ECL	Electrochemiluminescent
ECOG	Eastern Cooperative Oncology Group
eGFR	Epidermal Growth Factor Receptor
EIU	Exposure In-Utero
EOS	End of Study
FDA	Food and Drug Administration
FLT3	Fms-Related Tyrosine Kinase 3
FNA	Fine Needle Aspirates
FOB	Functional Observation Battery
g	Gram
GCP	Good Clinical Practice
HDPE	High-Density Polyethlyene
hERG	human Ether-a-go-go Related Gene
Hg	Mercury
HGF	Hepatocyte Growth Factor
HIF	Hypoxia-Inducible Factor
HIV	Human Immunodeficiency Virus
HR	Hazard Ratio
hr	Hour
IC_{50}	Half Maximal Inhibitory Concentration
ICF	Informed Consent Form
ICH	International Conference on Harmonization
IFN-α/γ	Interferon-Alpha/Gamma
IgG4	Immunoglobulin G4
IL-1ß/2/6/8/12/18	Interleukin-1 Beta/2/6/8/12/18
ILD	Interstitial Lung Disease

LIST OF ABBREVIATIONS (CONTINUED)

IND	Investigational New Drug
INR	International Normalized Ration
IP-10	Interferon Gamma-Induced Protein 10
irAEs	Immune-Related Adverse Events
IRB	Institutional Review Board
IUD	Intrauterine Device
IV	Intravenous
kg	Kilogram
L	Liter
LDH	Lactate Dehydrogenase
LVEF	Left Ventricular Ejection Fraction
mAb	Monoclonal Antibody
MDSC	Myeloid-Derived Suppressor Cells
MedDRA	Medical Dictionary for Regulatory Activities
mg	Milligram
mL	Milliliter
mm	Millimeter
MRI	Magnetic Resonance Imaging
mRNA	Messenger Ribonucleic Acid
MSC	Mesenchymal Stem Cell
mTOR	Mammalian Target of Rapamycin
mTPI	Modified Toxicity Probability Interval
MUGA	Multigated Acquisition
NCI	National Cancer Institute
NE	Not Evaluable
NK	Natural Killer
NSCLC	Non-Small Cell Lung Cancer
NYHA	New York Heart Association
OS	Overall Survival
pCR	Pathological Complete Response
PD	Progressive Disease
PD-1	Programmed Cell Death Protein 1
PDGFR	Platelet-Derived Growth Factor Receptor
PD-L	Programmed-Death Ligand

LIST OF ABBREVIATIONS (CONTINUED)

PFS	Progression-Free Survival
P-gp	P-glycoprotein
PK	Pharmacokinetic
PK/PD	Pharmacokinetic/Pharmacodynamic
PKAP	Pharmacokinetic Analysis Plan
PPE	Palmar Plantar Erythrodysesthesia
PR	Partial Response
Preop	Preoperative
PS	Performance Status
PTT	Partial Thromboplastin Time
Q2W	Every 2 Weeks
QD	Once Daily
QTc	Corrected QT
RCC	Renal Cell Carcinoma
REB	Research Ethics Board
RECIST	Response Evaluation Criteria in Solid Tumors
RNA	Ribonucleic Acid
RR Interval	Inter-beat (R-R) Interval
SAE	Serious Adverse Event
SAP	Statistical Analysis Plan
SD	Stable Disease
SOC	System Organ Class
SUSAR	Serious Unexpected Serious Adverse Reaction
T-cell	Thymus cell
$t_{1/2}$	Plasma Half-Life
TAM	TYRO3, AXL and MER
TCR	T-cell Receptor
TEAE	Treatment-Emergent Adverse Event
TESAE	Treatment-Emergent Serious Adverse Event
TGF-β	Transforming Growth Factor-Beta
Th1	Type 1 T Helper Cells
TILs	Tumor Infiltrating Lymphocytes
TKI	Tyrosine Kinase Inhibitor
T_{max}	Time to Maximum Observed Concentration

LIST OF ABBREVIATIONS (CONTINUED)

TME	Tumor Microenvironment
TNF-α	Tumor Necrosis Factor-Alpha
Treg	Regulatory T-cell
TRK	Tropomyosin Receptor Kinase
TSH	Thyroid-Stimulating Hormone
ULN	Upper Limit of Normal
USP	United States Pharmacopeia
USPI	United States Package Insert
VEGF	Vascular Endothelial Growth Factor
VEGFR/-2	Vascular Endothelial Growth Factor Receptor/-2
VHL	von Hippel-Lindau
V_{ss}	Apparent Volume of Distribution at Steady State
WBC	White Blood Cell
WHO	World Health Organization
WOCBP	Women of Child Bearing Potential
µM	Micromolar

1 INTRODUCTION AND RATIONALE

1.1 Background

1.1.1 Renal Cell Carcinoma

Renal cell carcinomas (RCCs), which originate within the renal cortex, are responsible for 80% to 85% of all primary renal neoplasms (Garfield-2018). In the United States, RCC is the 6th most common cancer in men and the 9th most common cancer in women (Siegel-2018). An estimated 63,340 new cases and 14,970 deaths are expected in 2018 (American Cancer Society-2018). Incidence rates of RCC have been rising, with most of the increases occurring in early-stage tumors (Garfield-2018). Clear cell RCC (ccRCC) is the most common RCC histologic type, representing between 70% and 85% of all RCCs, and is overwhelmingly a nonhereditary (sporadic) cancer (Muglia-2015; Escudier-2014). ccRCC is characterized by two important features. First, approximately 50% of sporadic ccRCC is associated with inactivating mutations of the von Hippel-Lindau (VHL) tumor suppressor gene, which leads to upregulation of hypoxia-inducible factors (HIF) and to downstream upregulation of vascular endothelial growth factor (VEGF), resulting in increased angiogenesis (Rini-2005; Cohen-2012; Fischer-2015). VHL inactivation also leads to overexpression of receptor tyrosine kinases MET and AXL, which have been implicated in RCC pathogenesis, prognosis, and resistance to standard anti-angiogenic therapies (Tannir-2017). Second, ccRCC is often susceptible to immune regulation (Cho-2017). These two features have led to the use of several active agents that either target the VEGF receptor (VEGFR) pathway (e.g., sunitinib, pazopanib) or modulate immune response (e.g., interferon-alpha [IFN-α], interleukin-2 [IL-2], nivolumab) (Rini-2016). Here, we postulate that the combination of sitravatinib, an inhibitor of VEGFR and other related kinases, and nivolumab, a Programmed Cell Death Protein 1 (PD-1) inhibitor, is not only active due to each component, but that the addition of sitravatinib can further enhance the immune effects from nivolumab.

1.1.2 Sitravatinib

Sitravatinib is a receptor tyrosine kinase inhibitor (TKI) that targets multiple closely related receptor tyrosine kinase pathways including VEGFR, platelet-derived growth factor receptor (PDGFR), c-KIT, c-MET, and the TAM family of receptors (TYRO3, AXL and MER).

In an ongoing Phase 1/1b study of sitravatinib, clinical activity has been observed in patients with ccRCC, putatively due to sitravatinib's potent inhibition of VEGFR2 and inhibition of MET and AXL, which may mediate resistance to VEGFR inhibitors, and in patients with qualifying genetic alterations selected based on sitravatinib's target profile.

1.1.3 Nivolumab

Nivolumab is a monoclonal antibody directed against the Programmed Cell Death Protein 1 (PD-1), an inhibitory receptor expressed on activated T-cells, and blocks the interaction

between PD-1 and its ligands, thereby releasing PD-1-mediated inhibition of T-cell proliferation (including cytotoxic CD8+ T-cells) and cytokine production.

Binding of the PD-1 ligands, PD-L1 and PD-L2, to the PD-1 receptor expressed on T cells, inhibits T-cell proliferation and cytokine production and helps to regulate normal immune tolerance. The PD-1 receptor along with the ligands PD-L1 and PD-L2 constitute an immune checkpoint pathway that inhibits T-cell activation when engaged (Mellman-2011; Topalian-2015). PD-L1 functions to limit collateral damage to normal tissues after an immune response has been triggered. Upregulation of PD-1 ligands is utilized by tumors to help evade detection and elimination by the host immune system tumor response.

Immune checkpoint inhibitors targeting the PD-1/PD-L1 pathway have demonstrated clinical activity across a range of cancer types, including RCC (Motzer-2015; Sharma-2016; Sharma-2017; Rosenberg-2016; Patel-2018; Massard-2016; Bellmunt-2017; Apolo-2017; Balar-2017a; Balar-2017b). In ccRCC, nivolumab was investigated in patients previously treated with anti-angiogenic therapy in the Phase 3 CheckMate 025 trial at a dose level of 3 mg/kg every 2 weeks. Nivolumab treatment was associated with a median OS of 25.0 months compared to 19.6 months for patients treated with everolimus, a mammalian target of rapamycin (mTOR) inhibitor, with a hazard ratio (HR) of 0.73 (98.5% CI: 0.57, 0.93; p=0.002). Nivolumab has been approved for use in this treatment setting (Motzer-2015).

1.1.4 Potentially Synergistic Effects

Together, sitravatinib and nivolumab may cooperate to elicit greater anti-tumor activity than either agent alone, as sitravatinib is predicted to inhibit multiple targets that enhance several steps in the cancer immunity cycle which may in turn augment the efficacy of nivolumab.

First, the anti-tumor activity of sitravatinib may promote the release of tumor antigens.

Second, inhibition of the split kinase receptors may further enhance anti-tumor immunoreactivity by depletion of the populations of two key cellular mediators of suppression of anti-tumor immunity (Farsaci-2012; Kujawski-2010; Ozao-Choy-2009). Specifically, inhibition of VEGFR2 may decrease the number of regulatory T-cells (Tregs) within the tumor microenvironment (TME) (Terme-2013; Finke-2008), and inhibition of the KIT and VEGFR family members may decrease the number of myeloid-derived suppressor cells (MDSCs) (Garton-2017; Kao-2011; Ko-2009; Pan-2008; Ozao-Choy-2009). Depletion of these two populations may promote the expansion and migration of anti-tumor cytotoxic T-cells, and their infiltration into tumor tissue.

Several studies support the potential role of these two cell types in creating an immunosuppressive TME that allows tumors to evade an immune response. Resistance to antiangiogenic therapy has been attributed to the action of Tregs in the TME, which prevent tumors from mounting an active immune response despite tumor infiltration by

CD4+ and CD8+ cells (Liu-2015). Additionally, increases in circulating MDSCs have been found to be the most predominant change in the peripheral blood of untreated ccRCC patients prior to nephrectomy, with elevations nearly 2 to 5-fold above the average levels measured in healthy volunteers (Gustafson-2015; Najjar-2017), and intratumoral and peritumoral presence of MDSCs is an independent prognostic factor for decreased survival in a cohort of 375 ccRCC patients (Gustafson-2015). TKI treatment before nephrectomy in patients with ccRCC has been found to increase tumor-infiltrating lymphocyte (TIL) expansion associated with depletion of intratumoral MDSCs, providing evidence to support the functional link between the two (Guislain-2015). In addition to the potential effects of VEGFR2 inhibition to modulate subsets of immune cells, VEGFR2 inhibition may have other key effects, including increased tumor perfusion that may facilitate trafficking of TILs into the tumor and upregulation of PD-L1 expression (Liu-2015) which may facilitate tumor targeting by nivolumab.

Third, sitravatinib may reverse the immunosuppressive effects within the TME that are mediated by the TAM receptors through inhibition of MERTK, resulting in an increased number of M1- versus M2-polarized macrophages and release of IL-12, IL-6 and TNF-α which enhance CD8+ T-cell activation, and through the inhibition of AXL, which is predicted to increase the Toll-like receptor dependent inflammatory response in dendritic cells (Lemke-2008; Camenisch-1999). Also, inhibition of MERTK and AXL may result in increased natural killer (NK) cell activity by releasing the negative feedback regulatory mechanism (Paolino-2014) and may complement PD-1/PD-L1 checkpoint inhibition to augment the host anti-cancer immune response.

Finally, inhibition of MET may enhance the anti-tumor response by restoring antigen-presenting cell (APC) function and reducing or eliminating MDSCs within the TME (Chen-2014; Yen-2013; Okunishi-2005; Singhal-2011; Benkhoucha-2010).

Thus, the combination of sitravatinib and nivolumab is a rational approach for patients with ccRCC with effects at several steps in the immunity cycle, including the potential for sitravatinib to inhibit several immune cell types that normally function to suppress immune responses and underlie resistance to checkpoint blockade (Vanneman-2012), including Tregs, myeloid-derived suppressor cells (MDSCs), and M2-polarized macrophages.

This combination has been shown to be safe and tolerable in an ongoing Phase 2 Study (MRTX-500) in second-line metastatic non-small cell lung cancer (NSCLC) patients, where the recommended Phase 2 dose for this combination has been established as 120 mg orally daily of sitravatinib and 240 mg of nivolumab (flat dose) administered IV every 2 weeks (Q2W).

1.1.5 Neoadjuvant Setting in Locally-Advanced ccRCC

At the time of diagnosis, approximately 25% of patients with ccRCC have locally-advanced disease (Errarte-2016). Radical or partial nephrectomy with curative intent remains a keystone in the treatment of localized RCC (Rini-2009; Choueiri-2011). Patients with localized ccRCC are typically treated with surgical resection, with a 5-year

survival rate dependent on the disease stage, ranging from 81% for Stage I to 53% for Stage III (American Cancer Society-2017). However, there is a relatively high rate of recurrence (Escudier-2014). Although adjuvant therapy with sunitinib may improve disease-free survival in patients with locally-advanced ccRCC (Ravaud-2016; Motzer-2017), the benefit is modest, and given the poor prognosis of metastatic ccRCC despite recent advances with immunotherapy and other targeted therapies (Incorvaia-2016), there remains an unmet medical need for patients with locally-advanced ccRCC.

Neoadjuvant, or preoperative, therapy can lead to primary tumor downsizing or downstaging, which may decrease intra-operative and peri-operative morbidity and potentially allow for less extensive surgery or less invasive approaches (Sciarra-2012). In addition, in locally-advanced RCC, neoadjuvant therapy can potentially control micrometastatic disease present at the time of surgery. Notably, aggressive solid tumor mouse models have revealed a survival benefit with short courses of neoadjuvant over adjuvant PD-1 blockade (Liu-2016).

Multiple clinical trials have or are evaluating the role of targeted agents in the neoadjuvant setting (Choueiri-2011). In Phase 2 trials in both locally-advanced and metastatic ccRCC, neoadjuvant therapy has been associated with a reduction of primary tumor burden prior to surgery. Neoadjuvant axitinib, a VEGFR TKI, administered for 12 weeks is clinically active and reasonably well-tolerated in the neoadjuvant setting in patients with locally-advanced ccRCC (28% median reduction of primary renal tumor diameter, with all patients experiencing tumor reduction and with a partial response [PR] rate of 46%) (Karam-2014). Similarly, neoadjuvant sunitinib, a VEGFR and PDGFR TKI, administered for 8 weeks, is safe and feasible in the neoadjuvant setting in patients with locally-advanced or metastatic ccRCC, and active, demonstrating a 21% median reduction of primary renal tumor diameter, with all patients experiencing tumor reduction and 29% with a PR (Silberstein-2010).

Similarly, neoadjuvant immune checkpoint inhibitors are being investigated in two ongoing Phase 2 studies of perioperative nivolumab in non-metastatic ccRCC patients which have shown preliminary feasibility and safety with no surgical delays or complications (Harshman-2017). And a Phase 3 randomized study comparing perioperative nivolumab to observation in patients with localized RCC undergoing nephrectomy is currently under way (Harshman-2017).

In summary, in addition to sitravatinib's potent inhibition of VEGFR and inhibition of MET and AXL (which potentially mediate resistance to VEGFR inhibitors), sitravatinib may act synergistically with immune checkpoint inhibitors such as nivolumab to augment immune activity in ccRCC. Treatment of ccRCC with sitravatinib may produce rapid tumor cell death and release of tumor associated antigens as well as enhance tumor antigen presentation, inhibit immunosuppressive cell types and prime and activate anti-tumor T-cell responses.

This study (516-002) will evaluate the clinical activity of this combination as neoadjuvant treatment of ccRCC and assess correlative immune effects. Nivolumab will be

administered in accordance with approved labeling, by IV infusion, 240 mg Q2W. Sitravatinib will be administered in accordance with findings of the prior nivolumab combination study (MRTX-500), orally, at a dose of 120 mg QD. Non-clinical studies indicate that the 120 mg dose of sitravatinib should, based on degree of inhibition using cellular assays, achieve the plasma exposure required for inhibition of VEGF, TAM, KIT and MET receptors, necessary to achieve anti-tumor efficacy in the combination setting.

1.2 Sitravatinib Background

Sitravatinib (MGCD516) is an orally available, potent small molecule inhibitor of a closely related spectrum of receptor tyrosine kinases including MET, AXL, MERTK, VEGFR family, PDGFR family, KIT, fms-related tyrosine kinase 3 (FLT3), TRK family, RET, DDR2, and selected EPH family members. Receptor tyrosine kinases are key regulators of signaling pathways leading to cell growth, survival, and migration (Blume-Jensen-2001). These kinases are dysregulated in many cancers through overexpression, genetic alteration or co expression with high affinity ligands (Blume-Jensen-2001). Multiple sitravatinib receptor tyrosine kinase targets are genetically altered in a variety of cancers and act as oncogenic drivers, promoting cancer development and progression. In addition to the immunostimulatory effects of AXL and MET inhibition, sitravatinib may further condition the TME in favor of anti-tumor activity by its immunomodulatory effects mediated through VEGFR and KIT inhibition. Preclinical data with sitravatinib indicate that it can increase expression of PD-L1 on tumor cells *in vitro* and *in vivo*. Pilot studies in syngeneic mouse tumor models also suggest that sitravatinib increases the proliferation and fraction of systemic/spleen CD4+ and CD8+ T lymphocytes and reduces the number of systemic MDSCs. Additional studies to investigate the effects of sitravatinib in the TME are ongoing or planned.

Background information in addition to that presented below is available in the Sitravatinib (MGCD516) Investigator's Brochure.

1.2.1 Sitravatinib Drug Substance

The chemical structure and chemical formulation of sitravatinib (MGCD516) is as follows:

MGCD516 Free Base

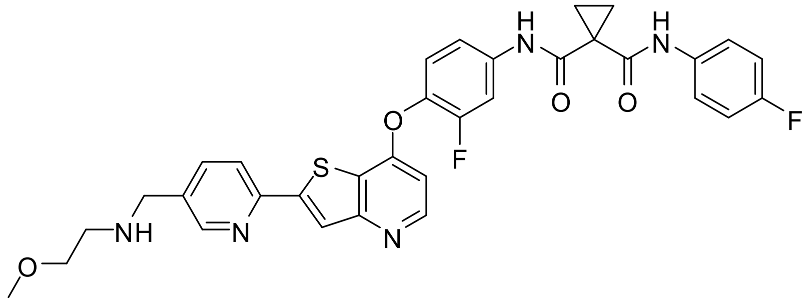

Chemical Formula: $C_{33}H_{29}F_2N_5O_4S$
Molecular Weight: 629.68 g/mol

1.2.2 Sitravatinib Non-Clinical Data

Sitravatinib demonstrated potent, concentration-dependent inhibition of the kinase activity of MET, AXL, MERTK, VEGFR family, PDGFR family, KIT, FLT3, TRK family, RET, DDR2, and selected EPH family members in biochemical assays and inhibited phosphorylation and kinase dependent function in cell-based assays. Sitravatinib also inhibited oncogenic functions associated with target receptor tyrosine kinases including MET-dependent cell viability and migration and endothelial tube formation and angiogenesis. Consistent with this anti-tumor and anti-angiogenic mechanism of action, sitravatinib demonstrated anti-tumor efficacy over a broad spectrum of human tumor xenograft models including robust cytoreductive anti-tumor activity in a subset of models exhibiting genetic alterations in receptor tyrosine kinase targets including MET, RET, FLT3 and others.

In vitro results from the hERG (human ether-a-go-go related gene) assay demonstrate an IC_{50} of 0.6 μM on the potassium current, which far exceeds exposures observed clinically. There were no adverse effects on the cardiovascular system, including no effect on the QTc interval, when sitravatinib was administered to dogs at doses up to 4 mg/kg (mean 6 hr concentration of 0.072 μg/mL). Minor increases in vascular pressures were observed during the dog cardiovascular study; however, these were mild and considered of limited biological consequence. Assessment of the neurological functional observation battery (FOB) and respiratory evaluations (tidal volume, respiration rate, and minute volume) in rats did not reveal any sitravatinib-related effects at doses up to 25 mg/kg.

In a bidirectional permeability study with Caco-2 cell lines, MGCD516 is classified as a highly permeable compound, and not a substrate of P-glycoprotein (P-gp) and Breast Cancer Resistance Protein (BCRP). A P-gp and BCRP inhibition study using Caco-2 cells suggested that MGCD516 is a significant inhibitor of P-gp and BCRP with IC_{50} value of 0.838 and 1.51 μM, respectively, these values are much higher than the systemic steady state exposure levels observed clinically.

Using an ultra-centrifugation technique sitravatinib was 98.6% bound to human plasma proteins.

MGCD516 was evaluated for cytochrome P450 (CYP)-mediated metabolism using human liver microsomes and recombinant human enzymes. Results suggest that multiple enzymes, including CYP1A2, CYP2A6, CYP2B6, CYP2C8, CYP2C9, CYP2D6, CYP2E1, and CYP3A4 are involved in the metabolism of MGCD516.

The effect of treating primary cultures of cryopreserved human hepatocytes with MGCD516 on the expression of CYP enzymes was investigated. Overall, treatment of cultured human hepatocytes with up to 30 μM MGCD516 caused little or no increase (< 2.0-fold change or < 20% of the positive control) in CYP1A2 activity, CYP1A2 mRNA (messenger ribonucleic acid) levels, or CYP3A4 activity. However, MGCD516 (up to 3 and 10 μM) caused concentration dependent increases (> 2-fold change and

> 20% of the positive control) in CYP2B6 activity, CYP2B6 mRNA levels, and CYP3A4 mRNA levels in one or more human hepatocyte cultures.

There was little or no evidence of direct inhibition of CYP1A2, CYP2A6 or CYP2E1 by MGCD516 or of time- or metabolism-dependent inhibition of any of the CYP enzymes evaluated. Under the experimental conditions examined, MGCD516 demonstrated direct inhibition of CYP2C8, CYP2C9, CYP2C19, CYP2D6 and CYP3A4/5 (as measured by testosterone 6β-hydoxylation and midazolam 1′-hydroxylation) with IC_{50} (half maximal inhibitory concentration) values of 2.9 µM, 11 µM, 10 µM, 1.9 µM, 11 µM and 0.81 µM, respectively. In addition, approximate 50% direct inhibition was observed for CYP2B6 at the highest concentration of MGCD516 evaluated (20 µM); thus, the IC_{50} value was reported as greater than 20 µM.

Because the potency for MGCD516 against its intended clinical targets is generally less than 0.1 µM, it may be unlikely that concentrations required for robust direct systemic inhibition/induction of the tested CYPs will be achieved at projected clinical dose and exposure levels.

1.2.3 Sitravatinib Clinical Data

1.2.3.1 Sitravatinib Pharmacokinetics

After single dose administration, sitravatinib reaches peak concentration in a median time of 3 to 9 hours. Exposure parameters (maximum concentration [C_{max}] and area under the curve [AUC]) are dose proportional with doses up to 200 mg. Mean elimination half-life varies between 40 and 52 hours after oral administration. Drug accumulation is observed after multiple dose administration; mean accumulation ratios across the seven dose levels (10 mg to 200 mg QD) vary between 1.9 and 4.1 for C_{max} and 2.4 and 5.6 for AUC_{0-24}. Based on current data following administration of 150 mg once daily to patients, the steady state mean C_{max}, $C_{ave,ss}$ and $AUC_{0-24,ss}$ values are 114 ng/mL, 94 ng/mL and 2.26 µg.h/mL, respectively.

1.2.3.2 Sitravatinib Clinical Safety

Sitravatinib monotherapy and sitravatinib in combination with nivolumab are being evaluated as part of the clinical development program.

Study 516-001 is a Phase 1/1b clinical trial of sitravatinib as monotherapy in patients with advanced solid tumor diseases. The Phase 1 dose-escalation study evaluated dose levels between 10 mg and 200 mg daily. The Phase 1b expansion included patients having tumors with selected histological diagnoses and/or molecular markers solid tumors. As of 26 June 2017, among the 86 patients treated at a higher dose of 150 mg QD and for whom safety data were available, treatment-related adverse events reported in ≥ 10% of patients in this Phase1/1b study include fatigue (44%), diarrhea (35%), hypertension (34%), nausea (24%), decreased appetite (22%), vomiting (22%), decreased weight (17%), palmar-plantar erythrodysesthesia (13%), and hypothyroidism (12%). Dose-limiting toxicity (DLT) was reported in four patients who developed palmar-plantar

erythrodysesthesia, neuropathy, mucositis, or fatigue. Grade 3 adverse events (AEs) reported as related to treatment in more than one patient included hypertension (19%); diarrhea (11%); pulmonary embolism, increased lipase, and fatigue (5% each); palmer-plantar erythrodysesthesia, decreased ejection fraction, left ventricular dysfunction, mucosal inflammation, and hyponatremia (2% each). Grade 4 AEs reported as related to treatment in 1 patient each (1%) included febrile neutropenia, hypotension and tachycardia. Treatment-related serious adverse events (SAEs) were reported in 12 patients (14%) and included diarrhea (4%); pulmonary embolism, hypertension and fatigue (2% each); back pain, cardiac failure, ejection fraction decreased, febrile neutropenia, hiccups, hypertension, hypoalbuminemia, hypokalemia, hypotension, left ventricular dysfunction, odynophagia, oropharyngeal pain, pancreatitis, rash follicular and tachycardia (1% each). Twenty-six deaths were reported in this study, with the primary cause of death being the disease under study (n=17), unknown (n=5), hypoxic respiratory failure, respiratory failure, GI bleed or sepsis (n=1 each).

Study MRTX-500 evaluates sitravatinib in combination with the PD-1 inhibitor nivolumab in patients with advanced or metastatic non-squamous NSCLC who have experienced disease progression either on or after prior treatment with a checkpoint inhibitor therapy (CIT-experienced) or after treatment with a platinum-based doublet chemotherapy (CIT-naïve). The primary objective is to evaluate the clinical activity of nivolumab in combination with sitravatinib. The study began with a lead-in evaluation of sitravatinib in combination with nivolumab administered by intravenous (IV) infusion, 240 mg every 2 weeks. Sitravatinib is administered orally, once daily in cycles of 28 days. No protocol defined dose-limiting toxicities (DLTs) were reported in the first 6 evaluable patients treated at 120 mg QD, which was selected as the recommended Phase 2 dose. The Phase 2 portion of the study uses a Predictive Probability Design (Lee-2008) for each treatment arm and stratum. For patients who are CIT-experienced, enrollment is stratified by prior outcome of treatment with a checkpoint inhibitor: those with clinical benefit or no clinical benefit to prior checkpoint inhibitor therapy. Patients who are CIT-naïve are stratified according to their PD-L1 status: no/low PD-L1 expression or high PD-L1 expression. Sitravatinib is administered orally, 120 mg once daily in cycles of 28 days and nivolumab is administered by IV infusion, 240 mg every 2 weeks. As of 26 June 2017, among the 10 patients for whom safety data are available, sitravatinib-related AEs reported in ≥20% include fatigue (50%), diarrhea (40%), aspartate aminotransferase (AST) increase (30%), palmar-plantar erythrodysesthesia (30%), dysphonia (30%), lipase increase (20%), weight decrease (20%), nausea (20%), vomiting (20%), decreased appetite (20%), hyponatremia (20%), hypertension (20%) and hypothyroidism (20%). One patient experienced SAEs of pulmonary embolism and deep-vein thrombosis attributed to sitravatinib treatment. No immune-related AEs (irAEs) or patient deaths have been reported. The study has met the criterion for expansion to Stage 2 of patient enrollment and is ongoing.

Non-clinical toxicology studies as well as clinical safety data from the Phase 1/1b and Phase 2 studies suggest that AEs associated with sitravatinib are similar to those observed with other small molecule inhibitors of the VEGFR pathway.

Based on reported clinical experience with sitravatinib and similar agents, and non-clinical data with sitravatinib, guidance to the Investigator is provided for selected AEs in Section 5.3.

1.2.3.3 Sitravatinib Clinical Efficacy

Efficacy results are awaited from both the Phase 1b segment of Study 516-001, and Study MRTX-500.

1.3 Nivolumab Background

Nivolumab (OPDIVO®) is a human monoclonal antibody that blocks the interaction between PD-1 and its ligands, PD-L1 and PD-L2. Nivolumab is an immunoglobulin G4 (IgG4) kappa immunoglobulin.

Binding of the PD-1 ligands, PD-L1 and PD-L2, to the PD-1 receptor found on T-cells, inhibits T-cell proliferation and cytokine production. Upregulation of PD-1 ligands occurs in some tumors and signaling through this pathway can contribute to inhibition of active T-cell immune surveillance of tumors. Nivolumab is a human IgG4 monoclonal antibody that binds to the PD-1 receptor and blocks its interaction with PD-L1 and PD-L2, releasing PD-1 pathway-mediated inhibition of the immune response, including the anti-tumor immune response. In syngeneic mouse tumor models, blocking PD-1 activity resulted in decreased tumor growth.

Background information in addition to that presented below is available in the OPDIVO® US Prescribing Information (USPI OPDIVO [nivolumab]).

1.3.1 Nivolumab Drug Substance

Generic Name: Nivolumab

Other Name: OPDIVO®

Molecular Weight: 146 kDa

1.3.2 Nivolumab Non-Clinical Data

The non-clinical experience is described in the OPDIVO® US Prescribing Information (USPI OPDIVO [nivolumab]).

1.3.3 Nivolumab Clinical Data

The following reports information included in the OPDIVO® US Prescribing Information (USPI OPDIVO [nivolumab]) dated March 2018. Refer to the current Prescribing Information for updates during the conduct of this clinical trial.

1.3.3.1 Nivolumab Pharmacokinetics

The PK of single-agent nivolumab was studied in patients over a dose range of 0.1 to 20 mg/kg administered as a single dose or as multiple doses of OPDIVO® every 2 or 3 weeks. The geometric mean (% coefficient of variation [CV%]) clearance (CL) is 8.2 mL/h (53.9%), geometric mean volume of distribution at steady state (V_{ss}) is 6.8 L (27.3%), and geometric mean elimination half-life ($t_{1/2}$) is 25 days (77.5%). Steady-state concentrations of nivolumab were reached by 12 weeks when administered at 3 mg/kg every 2 weeks, and systemic accumulation was approximately 3.7-fold. The exposure to nivolumab increased dose proportionally over the dose range of 0.1 to 10 mg/kg administered every 2 weeks.

The population PK analysis suggested that the following factors had no clinically important effect on the clearance of nivolumab: age (29 to 87 years), weight (35 to 160 kg), gender, race, baseline lactate dehydrogenase (LDH), PD-L1 expression, tumor type, tumor size, renal impairment, and mild hepatic impairment.

The effect of renal impairment on the clearance of nivolumab was evaluated by a population PK analysis in patients with mild (estimated Glomerular Filtration Rate (eGFR) 60 to 89 mL/min/1.73 m^2; n=313), moderate (eGFR 30 to 59 mL/min/1.73 m^2; n=140), or severe (eGFR 15 to 29 mL/min/1.73 m^2; n=3) renal impairment. No clinically important differences in the clearance of nivolumab were found between patients with renal impairment and patients with normal renal function.

The effect of hepatic impairment on the clearance of nivolumab was evaluated by population PK analyses in patients with mild hepatic impairment (total bilirubin n less than or equal to the upper limit of normal (ULN) and AST greater than ULN or total bilirubin less than 1 to 1.5 times ULN and any AST; n=92). No clinically important differences in the clearance of nivolumab were found between patients with mild hepatic impairment and patients with normal hepatic function. Nivolumab has not been studied in patients with moderate (total bilirubin greater than 1.5 to 3 times ULN and any AST) or severe hepatic impairment (total bilirubin greater than 3 times ULN and any AST).

1.3.3.2 Nivolumab Anti-Drug Antibodies

Of 2085 patients who were treated with OPDIVO®, as a single agent, 3 mg/kg every 2 weeks and evaluable for the presence of anti-nivolumab antibodies, 233 patients (11.2%) tested positive for treatment-emergent anti-nivolumab antibodies by an electrochemiluminescent (ECL) assay and 15 patients (0.7%) had neutralizing antibodies against nivolumab. There was no evidence of altered pharmacokinetic profile or increased incidence of infusion reactions with anti-nivolumab antibody development.

1.3.3.3 Nivolumab Adverse Reactions Common in Clinical Trials

Refer to the current OPDIVO® US Prescribing Information (USPI OPDIVO [nivolumab]), Highlights of Prescribing Information and Section 6.1 for information concerning adverse reactions occurring in clinical trials. As of March 2018, the most

common adverse reactions (≥20%) in patients administered OPDIVO® as a single agent were fatigue, rash, musculoskeletal pain, pruritus, diarrhea, nausea, asthenia, cough, dyspnea, constipation, decreased appetite, back pain, arthralgia, upper respiratory tract infection, pyrexia, headache, and abdominal pain.

1.3.3.4 Nivolumab Immune-Related Adverse Events

Refer to the current OPDIVO® US Prescribing Information (USPI OPDIVO [nivolumab]), Section 5, for information concerning irAEs occurring during treatment with nivolumab. As of March 2018, the OPDIVO® US Prescribing Information (USPI OPDIVO [nivolumab]) includes warnings and precautions for a range of irAEs documented in patients across cancer indications, including, pneumonitis, colitis, hepatitis, endocrinopathies (such as hypophysitis, adrenal insufficiency, hypothyroidism and hyperthyroidism, Type 1 diabetes mellitus), nephritis and renal dysfunction, rash, encephalitis, other rarer irAEs, and infusion reactions.

1.4 Combination Safety of Sitravatinib and Nivolumab

1.4.1 Potential for Drug-Drug Interactions

Sitravatinib administered in combination with nivolumab is unlikely to result in clinically relevant drug-drug interactions (DDI) based on absorption, metabolism, elimination or protein binding. Nivolumab is a mAb and is intravenously administered, whereas sitravatinib is a small molecule therapeutic administered orally; no absorption interactions are expected.

No studies on the metabolism of nivolumab have been reported *in vitro* or in humans. Like most therapeutic proteins, nivolumab is not expected to be metabolized by liver CYP or other drug metabolizing enzymes and is unlikely to have an effect on CYPs or other metabolizing enzymes in terms of inhibition or induction.

MGCD516 inhibits P-gp and BCRP transporters at high concentrations and, as a result, sensitive substrates and substrates with narrow therapeutic index for P-gp and BCRP transporters may be used, but with caution during study drug treatment. Similarly, preclinical results suggest that multiple enzymes, including CYP 1A2, 2A6, 2B6, 2C8, 2C9, 2D6, 2E1, and 3A4 are involved in the metabolism of sitravatinib with a low risk of one enzyme contributing to metabolism in a disproportionate manner. Therefore, sensitive substrates and substrates with narrow therapeutic index for indicated CYP enzymes may be used, but with caution during study drug treatment. Finally, QTc prolongation with sitravatinib is under evaluation, though no signal has been detected from the ongoing sitravatinib clinical trials. Prior to results of such evaluation, drugs with a known risk of torsades de pointes should be avoided during study drug treatment, whereas drugs with conditional risk of torsades de pointes may be used, but with caution during study drug treatment. Full details of the medications or substances to be avoided or used with caution during study drug treatment are outlined in Appendix 2.

1.4.2 Evaluation of Potential for Increased Toxicities with Combination Use of Sitravatinib and Nivolumab

Frequent AEs, such as fatigue, musculoskeletal pain, decreased appetite, cough, and constipation, which are non-specific and typical of cancer treatment regimens have been observed with nivolumab and sitravatinib monotherapy. Potential exists for these AEs to be observed with increased severity or frequency during use of the combined agents. Management of these effects in patients receiving cancer therapy is well precedented.

More importantly, irAEs of Special Interest based on observed safety events using nivolumab monotherapy include pneumonitis, colitis, hepatitis, endocrinopathy, nephritis/renal dysfunction, rash, and encephalitis. While sitravatinib may have immunostimulatory effects, autoimmune adverse effects have not been reported in clinical trials of this investigational study agent, including to date in combination with nivolumab, nor are they recognized as class effects for this agent. However, the potential for sitravatinib to exacerbate or promote these AEs when administered in combination with nivolumab should be borne in mind. AE incidence data presented below are as reported in the OPDIVO® US Prescribing Information (USPI OPDIVO [nivolumab]) dated March 2018 and the Sitravatinib Investigator's Brochure dated September 2017. Updates to these data during the conduct of this clinical trial will be found in the current OPDIVO® US Prescribing Information (USPI OPDIVO [nivolumab]) and current Sitravatinib Investigator's Brochure.

A clinically relevant overlap in toxicity may arise between the immune-related colitis attributed to nivolumab and the non-specific, most often mild to moderate diarrhea observed with sitravatinib. Immune-related colitis has been reported in 2.9% (58/1994) of patients treated with nivolumab, with a median time to onset of 5.3 months (range: 2 days to 20.9 months). Diarrhea has been reported in approximately 35% of patients treated with sitravatinib, most often beginning within the first month of the start of treatment. Diarrhea (including colitis, enterocolitis, and gastroenteritis) of any grade has been reported with single-agent nivolumab to occur at approximately 25% of advanced RCC patients treated at 3 mg/kg Q2W in the CheckMate 025 clinical trial (USPI OPDIVO [nivolumab]; Motzer-2015). The time to onset may be helpful in distinguishing diarrhea that may be attributed to autoimmune effects versus non-specific toxicity.

Tyrosine kinase inhibitors in general, and MET inhibitors in particular, have been associated with non-specific, most often mild to moderate elevation in aspartate and alanine transaminases (AST and ALT). Mild to moderate elevations in liver transaminases have also been observed in less than 10% of patient treated with sitravatinib. The elevations observed with sitravatinib generally occur within the first 28 days of treatment and resolve with interruption of treatment. In patients receiving nivolumab as a single agent, immune-mediated hepatitis occurred in 1.8% (35/1994) of patients; the median time to onset was 3.3 months (range: 6 days to 9 months).

Nivolumab can cause autoimmune thyroid disorders including hypothyroidism, thyroiditis resulting in hypothyroidism, and hyperthyroidism. In patients receiving

nivolumab as a single agent, hypothyroidism or thyroiditis resulting in hypothyroidism occurred in 9% (171/1994) of patients; the median time to onset was 2.9 months (range: 1 day to 16.6 months). On the other hand, hyperthyroidism occurred in 2.7% (54/1994) of patients receiving nivolumab as a single agent; the median time to onset was 1.5 months (range: 1 day to 14.2 months). Hypothyroidism has been reported in approximately 12% (10/86) of subjects treated with sitravatinib.

A clinically relevant overlap in toxicity may arise between the immune-related rash attributed to nivolumab and the non-specific, most often mild (Grade 1) rash observed with sitravatinib. Rash, described as dermatitis, acneiform dermatitis, erythematous rash, generalized rash, macular rash, maculopapular rash, papular rash, pruritic rash, erythema multiforme, and erythema, of any grade has been reported in 28% of advanced RCC patients treated with nivolumab. Rash has been reported in 9% (8/86) of patients treated with sitravatinib.

2 STUDY OBJECTIVES

2.1 Objectives

2.1.1 Primary Objective

To evaluate the preoperative clinical activity of the combination of sitravatinib and nivolumab in patients with locally-advanced ccRCC undergoing nephrectomy.

2.1.2 Secondary Objectives

- To evaluate the safety and tolerability of the combination regimen in the selected population.
- To characterize the baseline tumor-related immune profile in patients with RCC.
- To determine the immune effects of sitravatinib and the combination regimen in patients with ccRCC.
- To evaluate the pharmacokinetics (PK) of sitravatinib administered alone and in combination with nivolumab in patients with ccRCC.
- To characterize the time to surgery.
- To evaluate secondary efficacy endpoints with the combination regimen in the selected population.

2.1.3 Exploratory Objective

- To evaluate changes in gene expression and the T-cell repertoire in response to therapy in patients with ccRCC.

2.2 Endpoints

2.2.1 Primary Endpoint

Percentage of patients achieving a point in time objective response (either CR or PR) prior to surgery.

2.2.2 Secondary Endpoints

- Safety characterized by type, incidence, severity, timing, seriousness and relationship to study treatment of adverse events (AEs) and laboratory abnormalities.
- Descriptive characterization of immune cell populations in the tumor and/or peripheral blood at baseline.
- Temporal changes in PD-L1 expression, selected cytokines and immune cell populations in the tumor and/or peripheral blood (including myeloid-derived suppressor cells [MDSCs], regulatory T-cells [Tregs], CD4+ [helper] and CD8+ [cytotoxic] T-cells, and the ratio of Type1:Type 2 tumor-associated macrophages).
- Blood plasma concentration of sitravatinib.
- Time-to-surgery.
- Disease free-survival (DFS).

2.2.3 Exploratory Endpoints

- Gene expression and T-cell receptor sequencing.

3 STUDY DESIGN

Study 516-002 is an open-label, non-randomized, preoperative window of opportunity Phase 2 study of sitravatinib and nivolumab in the neoadjuvant setting for the treatment of patients with locally-advanced ccRCC undergoing nephrectomy. The study will enroll approximately 25 subjects with imaging results consistent with a clinical diagnosis of locally-advanced renal cancer in order to identify and treat with the study drug treatment 18 clinical activity evaluable patients with ccRCC. Study drug treatment which will be conducted in 2 sequential preoperative treatment segments. During the first segment, patients will receive single-agent sitravatinib for 2 weeks, followed by a renal biopsy. Patients will then begin the second segment, consisting of combination treatment with sitravatinib and nivolumab for at least 4 weeks (and up to a maximum of 6 weeks to allow for flexibility with the scheduling of the planned nephrectomy). This segment will be followed by planned surgical resection by way of standard-of-care partial or radical nephrectomy. Patients will be followed for survival. The primary objective is to evaluate the clinical activity of the combination regimen using percentage of patients with ccRCC achieving a point in time objective response (either CR or PR) prior to surgery with the

combination treatment of sitravatinib and nivolumab. Objective responses will be assessed in accordance with RECIST 1.1. Secondary objectives include evaluation of safety and determination of immune effects of the combination treatment, along with PK of sitravatinib. The Schedule of Assessments to be performed in the study is presented in Table 1. The schedule for collection of PK/PD and ECG assessment time points are presented in Table 2.

Prior to Start of Treatment: All enrolled patients will have met all entry criteria and have completed baseline procedures including disease assessments and blood collection for baseline immune characteristics, and will be scheduled to undergo an initial diagnostic tumor biopsy of their renal lesion. Patients with a histology other than ccRCC will proceed to their End of Study (EOS) visit following the histology results, with no treatment with study drugs. Subjects with a clear cell histology will proceed to the study drug treatment which will be conducted in 2 sequential preoperative treatment segments as described below.

Segment 1 (2 weeks) consists of a 2-week treatment with single-agent sitravatinib. Sitravatinib will be administered at a dose of 120 mg orally QD, on a continuous daily dosing regimen. After completion of the 2-week dosing period, patients will undergo a renal biopsy.

Segment 2 (4-6 weeks) consist of combination treatment with sitravatinib and nivolumab for at least 4 weeks (and up to a maximum of 6 weeks to allow for flexibility with the scheduling of the nephrectomy). Sitravatinib will be administered at a dose of 120 mg orally daily, on a continuous daily dosing regimen for this entire segment. Nivolumab will be administered, in accordance with approved labeling, by IV infusion, 240 mg every Q2W (namely, on D15, D29, and potentially D43). Patients will receive the nivolumab injection on D43 only if their surgery is expected to occur more than a week from that date. Patients will be subject to a 2-day preoperative hold of all study drugs prior to surgery (such that the last dose of any drug is administered a minimum of 72 hours prior to surgery).

Of note, patients should be scheduled to undergo surgery no later than 8 weeks following their first dose of study drug, and the preoperative period (including Segments 1 and 2) should not be extended in order to take missed doses of either study drug.

Guidance for AE management and associated nivolumab treatment modifications are provided in product labeling. Guidelines for sitravatinib administration and dose reduction in the event of toxicity are provided in Section 5.1.

After completion of neoadjuvant therapy, patients will undergo planned nephrectomy. Patients will have their EOS visit following surgery, with no further study drug administration. Patients will be followed for disease-free survival for up to 3 years or more until either death, disease recurrence or loss to follow-up.

Further detail on sample collection and analyses are presented in Section 7.3 and the Study Laboratory Manual. The study will collect blood and tumor tissue collections as

described in the Schedule of Assessments (Table 1) depicted and the study schema in Figure 1. Protocol guidance of special interest is listed below.

- Freshly biopsied tumor tissue collection at pre-treatment is required for study drug treatment. Similarly, the on-treatment mid-study tumor biopsy on D14 is highly desirable for all treated patients. Archival samples cannot be submitted in lieu of pre-treatment samples. All attempts should be made to biopsy the same lesion on the mid-study biopsy as the initial biopsy.

- Tumor gene expression in freshly biopsied tumor samples will be determined using next generation sequencing performed by a central laboratory. For patients in whom tumor tissue has previously been tested using next generation sequencing, presence of specific tumor gene mutations and estimation of total mutation burden will be collected in the case report form (CRF).

4 SUBJECT SELECTION AND ENROLLMENT

Patient eligibility must be reviewed and documented by an appropriately qualified member of the Investigator's study team before patients are included in the study. No exceptions to the patient eligibility requirements will be granted by the Sponsor.

4.1 Inclusion Criteria

Patients must meet all of the following inclusion criteria to be eligible for enrollment into the study:

1. Imaging results consistent with locally-advanced renal cell carcinoma (RCC) without evidence of metastatic disease with absence of adjacent organ invasion or retroperitoneal adenopathy (cT2-T3b, N0, M0). Retroperitoneal lymph nodes ≤ 1cm in size each are considered N0.

2. Candidate for partial or complete nephrectomy that extirpates all tumor tissue as part of treatment plan.

3. Measurable disease as per RECIST version 1.1 (refer to Appendix 3).

4. Eastern Cooperative Oncology Group (ECOG) performance status (PS) 0 or 1 (refer to Appendix 1).

5. Adequate bone marrow and organ function demonstrated by:

   a. Absolute neutrophil count ≥ 1,000/mm^3 (≥ 1.0×10^9/L);

   b. Platelet count ≥ 100×10^9/L (≥ 100,000 per mm^3);

   c. ALT and AST ≤ 2.5 × ULN;

d. Serum bilirubin ≤ 1.5 × ULN, or ≤ 3.0 × ULN for patients with Gilbert Syndrome;

e. Serum creatinine ≤ 1.5 × ULN, or creatinine clearance (CrCl) ≥ 50 mL/min (using the Cockcroft-Gault formula or following local institutional standards).

6. ≥ 18 years of age.

7. Women of child-bearing potential (WOCBP) or men whose partner is a WOCBP agrees to use contraception while participating in this study, and for a period of 6 months following termination of study treatment.

8. Completed informed consent process, including signing Institutional Review Board/Ethics Committee/Research Ethics Board (IRB/EC/REB) approved informed consent form.

9. Willing to comply with clinical trial instructions and requirements.

4.2 Exclusion Criteria

Patients presenting with any of the following will not be included in the study:

1. Prior systemic anti-tumor treatment for RCC.

2. Patients who are receiving any other investigational agents.

3. Clinical status indicating that immediate surgery (within 6 weeks) is warranted regardless of whether neoadjuvant therapy is to be administered, as assessed by the treating surgeon.

4. Inability to undergo baseline tumor biopsy.

5. Active or prior documented autoimmune disease:

   a. Inflammatory bowel disease (e.g., Crohn's disease, ulcerative colitis);

   b. Interstitial lung disease (ILD), drug-induced ILD, radiation pneumonitis which required steroid treatment, or any evidence of clinically active interstitial lung disease;

   c. Other active or prior, documented autoimmune disease within the past 2 years. NOTE: Patients with Type I diabetes, vitiligo, Grave's disease, residual hypothyroidism due to autoimmune condition only requiring hormone replacement, or psoriasis not requiring systemic treatment (within the past 2 years) are not excluded.

6. Active or prior immunocompromising conditions:

   a. Current or prior use of immunosuppressive medication within 28 days before the first dose of study treatment, with the exceptions of intranasal and inhaled corticosteroids or systemic corticosteroids at physiological doses, which are not to exceed 10 mg/day of prednisone, or an equivalent dose of another corticosteroid;

   b. Known acute or chronic human immunodeficiency virus (HIV);

   c. History of primary immunodeficiency;

   d. History of allogeneic transplant.

7. Known acute or chronic hepatitis B or hepatitis C.

8. History of hypersensitivity to study treatment excipient.

9. History of severe hypersensitivity reaction to any monoclonal antibody.

10. Use of live vaccines against infectious disease (e.g., varicella) within 28 days of initiation of study therapy; killed vaccinations (e.g., influenza) are allowed at any appropriate time before and during the study.

11. Uncontrolled arterial hypertension (> 150 mm Hg systolic or > 100 mm Hg diastolic) on multiple observations despite standard of care treatment.

12. History of stroke or transient ischemic attack within the previous 6 months.

13. Any of the following cardiac abnormalities:

   a. Unstable angina pectoris;

   b. Congestive heart failure ≥ NYHA (New York Heart Association Functional Classification) Class 3;

   c. QTc > 480 milliseconds;

   d. Left ventricular ejection fraction (LVEF) ≤ 40%.

14. Concomitant medication known to cause prolonged QT which cannot be discontinued or changed to a different medication prior to enrollment (refer to Appendix 2).

15. Known or suspected presence of another malignancy that could be mistaken for the malignancy under study during disease assessments.

16. Pregnancy. WOCBP must have a negative serum or urine pregnancy test documented within the screening period prior start of study drug.

17. Breast-feeding or planning to breast feed during the study or within 6 months after study treatment.

18. Any serious illness, uncontrolled inter-current illness, psychiatric illness, active or uncontrolled infection, or other medical history, including laboratory results, which, in the Investigator's opinion, would be likely to interfere with the patient's participation in the study, or with the interpretation of the results.

4.3 Life Style Guidelines

Patients who are biologically capable of having children and sexually active must agree to use an acceptable method of contraception for the duration of the treatment period and for at least 6 months after the last dose of study treatment. The Investigator will counsel the patient on selection of contraception method and instruct the patient in its consistent and correct use. Examples of acceptable forms of contraception include:

1. Oral, inserted, injected or implanted hormonal methods of contraception, provided it has been used for an adequate period of time to ensure effectiveness;

2. Correctly placed copper containing intrauterine device (IUD);

3. Male condom or female condom used WITH a spermicide;

4. Male sterilization with confirmed absence of sperm in the post-vasectomy ejaculate;

5. Bilateral tubal ligation or bilateral salpingectomy.

The Investigator will instruct the patient to call immediately if the selected birth control method is discontinued or if pregnancy is known or suspected.

Note: Women are considered post-menopausal and/or not of child bearing potential if they have had 12 months of natural (spontaneous) amenorrhea with an appropriate clinical profile (e.g., age appropriate, history of vasomotor symptoms) or have had surgical bilateral oophorectomy (with or without hysterectomy) or tubal ligation at least 6 months ago. In case of any ambiguity, the reproductive status of the woman should be confirmed by hormone level assessment.

4.4 Enrollment into Study

Following completion of the informed consent process and review of all screening procedures, patient eligibility will be confirmed by appropriately qualified staff at the investigational site. Patients will be enrolled by entry into a patient registration log provided by the Sponsor and maintained by the study site and completion of the patient registration procedure detailed in the Site Operations Manual. Each patient will be assigned a sequential number by site. The patient number must be used on all documentation and correspondence with the Sponsor and Contract Research Organization (CRO).

5 STUDY TREATMENT

5.1 Sitravatinib Study Drug

5.1.1 Sitravatinib Formulation, Packaging and Storage

Sitravatinib will be provided by the Sponsor as 10 mg and 40 mg unit dose strength capsules. The composition of the drug product consists of a blend of MGCD516 free base drug substance, microcrystalline cellulose (Avicel® PH302), colloidal silicon dioxide (Aerosil® 200) and polysorbate 80 (Tween® 80). The blend is filled into Size 1 Light Blue Opaque (10 mg strength) or Swedish Orange Opaque (40 mg strength) hard gelatin capsules.

Sitravatinib drug product is packaged in 30-count, high-density polyethylene (HDPE), white opaque, round 60 cc bottles. A tamper-proof heat induction seal and a child-resistant closure are used. The provided bottles may be labeled for specific patient use and given to the patient if the capsule count is the needed number.

Sitravatinib medication labels comply with the legal requirements of the United States and will be printed in the languages required in the countries in which the study is conducted

Investigational clinical trial material should be stored in an area that is secure, with limited access and monitored for temperature using a calibrated thermostat or thermometer. Sitravatinib capsules should be stored under the conditions stated on the container labels and the Pharmacy Study Manual. Refer to the Pharmacy Study Manual for details.

5.1.2 Sitravatinib Preparation, Dispensing, Administration and Accountability

Only qualified personnel who are familiar with procedures that minimize undue exposure to them and to the environment should undertake the preparation, handling, and safe disposal of chemotherapeutic agents.

Study site personnel will dispense sitravatinib capsules on D1 and D29 of the study. Sufficient supply will be provided at each visit and extra capsules may be provided to cover an additional 2 days in case of delayed clinic visits or lost capsules. Study capsules will be dispensed in HDPE bottles provided by the Sponsor.

Sitravatinib capsules will be administered at a dose of 120 mg orally QD, on a continuous daily dosing regimen. Depending on safety observations, the sitravatinib dose may be reduced following Table 3.

The following guidelines should be followed for sitravatinib administration:

- Dosing in the morning is preferred;

- Capsules should be taken on an empty stomach (at least 2-hour fast before each dose and no food for a minimum of 1 hour after each dose);
- Capsules should be taken with at least 200 mL (1 cup) of water;
- Patients should swallow the capsules whole and not chew them;
- If vomiting occurs after dosing, sitravatinib doses should not be replaced.

On days when sitravatinib and nivolumab dosing are both scheduled, the daily dose of sitravatinib should precede nivolumab infusion for logistical reasons. This order of dosing is of most interest on days when blood sampling is scheduled for sitravatinib PK.

All sitravatinib study treatment supplies will be accounted for in the drug accountability inventory forms supplied by the Sponsor or using locally approved forms that include all required information. The drug accountability inventory forms must identify the study drug, including batch or lot numbers and account for its disposition on a patient-by-patient basis, including specific dates and quantities. The forms must be signed by the individual who dispensed the drug.

Patients will be asked to record their daily dosing on Sponsor provided diary cards and report any missed doses or lost doses at the next clinic visit. On the back of each Sponsor provided diary card, written dosing instructions for sitravatinib capsules are provided (e.g., fasting instructions, take with water, etc.). Patients should be told to bring study treatment bottle(s) (empty or not) and completed dosing diaries with them to the clinic visit for a compliance check and capsule count. Study site personnel will retain the bottle(s) until a monitor has completed reconciliation and retain dosing diaries with site study files, unless institutional policy prohibits such practice, in which case reconciliation should be carried by study staff and properly documented.

At the end of the study, all unused sitravatinib drug supplies must be destroyed in accordance with local Standard Operating Procedure provided to the Sponsor for the Trial Master File, or returned to the Sponsor or its appointed agent, as directed by the Sponsor.

5.1.3 Sitravatinib Dose Modification and Discontinuation

This protocol provides guidance for dose modification of sitravatinib (i.e., interruption, dose reduction or discontinuation) for AEs attributed to sitravatinib. For AEs due to nivolumab, see the current OPDIVO® US Prescribing Information (USPI OPDIVO [nivolumab]) and Section 5.2.4 for dose modifications.

Based on available safety and pharmacokinetic data from an ongoing Phase 1b trial in patients with various solid tumors and an ongoing Phase 2 trial in combination with nivolumab in patients with NSCLC, the dose and regimen recommended is 120 mg sitravatinib administered daily. If unacceptable toxicity is observed, the starting dose may be decreased during the study using the dose de-escalation rules described in Section 9.1.2 and the dose levels indicated in Table 3.

Guidelines for sitravatinib dose modifications to be implemented to manage adverse events are described in Section 5.3. Available dose levels for sitravatinib are outlined in Table 3. Once the dose has been reduced, re-escalation is generally not recommended but may be considered on a case-by-case basis. If the administration of sitravatinib is interrupted for reasons other than toxicity, then treatment with the study drug may be resumed at the same dose.

Table 3: Sitravatinib Sequential Dose Reductions for Individual Patients

120 mg once daily
80 mg once daily
60 mg once daily

Dose reduction below 60 mg QD may be undertaken after discussion with the Sponsor. If treatment with sitravatinib is withheld for ≥ 14 consecutive days, then permanent discontinuation from the study should be considered. If after receiving sitravatinib but prior to any nivolumab dosing, patients develop toxicities that prevent the first nivolumab administration, then permanent discontinuation from study should be considered. In contrast, patients who have already received nivolumab and who develop AEs (regardless of causality) preventing subsequent nivolumab administration may remain on study receiving sitravatinib alone.

5.2 Nivolumab Study Drug

Nivolumab will be obtained from commercial sources and managed in accordance with the OPDIVO® US Prescribing Information (USPI OPDIVO [nivolumab]). The following reports information and guidance included in the OPDIVO® US Prescribing Information dated March 2018. Refer to the current Prescribing Information for updates during the conduct of this clinical trial.

5.2.1 Formulation and Packaging

OPDIVO® is a sterile, preservative-free, non-pyrogenic liquid. OPDIVO® injection for IV infusion is supplied in single-dose vials. Each mL of OPDIVO® solution contains nivolumab 10 mg. Refer to the OPDIVO® US Prescribing Information (USPI OPDIVO [nivolumab]) for further detail.

5.2.2 Preparation and Dispensing

Visually inspect drug product solution for particulate matter and discoloration prior to administration. OPDIVO® is a clear to opalescent, colorless to pale-yellow solution. Discard the vial if the solution is cloudy, discolored or contains extraneous particulate matter other than a few translucent-to-white, proteinaceous particles. Do not shake the vial.

- Withdraw the required volume of OPDIVO® and transfer into an IV container.

- Dilute OPDIVO® with either 0.9% Sodium Chloride Injection, USP or 5% Dextrose Injection, USP to prepare an infusion with a final concentration ranging from 1 mg/mL to 10 mg/mL. The total volume of infusion must not exceed 160 mL.

- Mix diluted solution by gentle inversion. Do not shake.

- Discard partially used vials or empty vials of OPDIVO®.

The product does not contain a preservative. After preparation, store the OPDIVO® infusion either:

- at room temperature for no more than 8 hours from the time of preparation. This includes room temperature storage of the infusion in the IV container and time for administration of the infusion; or

- under refrigeration at 2°C to 8°C (36°F to 46°F) for no more than 24 hours from the time of infusion preparation.

Do not freeze.

5.2.3 Administration

The recommended dose of OPDIVO® is 240 mg administered as an IV infusion over 30 minutes Q2W (namely, on D15, D29, and potentially D43). Administer the OPDIVO® infusion through an IV line containing a sterile, non-pyrogenic, low protein binding in-line filter (pore size of 0.2 micrometer to 1.2 micrometer). Flush the IV line at end of infusion.

5.2.4 Dose Modification

Required dose modifications (i.e., interruption, dose reduction or discontinuation) for nivolumab should be performed per the current OPDIVO® US Prescribing Information (USPI OPDIVO [nivolumab]) Section 2.10 and Section 5, in addition to potential dose modifications for sitravatinib in accordance to Section 5.1.3 and Section 5.3.

If after receiving sitravatinib but prior to any nivolumab dosing, patients develop toxicities that prevent the first nivolumab administration, then permanent discontinuation from study should be considered. In contrast, patients who have already received nivolumab and who develop AEs (regardless of causality) preventing subsequent nivolumab administration may remain on study receiving sitravatinib alone.

5.3 Management of Adverse Events

Management of AEs due to nivolumab should be performed in accordance with the current OPDIVO® US Prescribing Information (USPI OPDIVO [nivolumab]) and Section 5.3.2. This protocol provides guidance for dose modification of sitravatinib

(i.e., interruption, dose reduction or discontinuation) for AEs attributed to sitravatinib (Section 5.1.3).

5.3.1 Sitravatinib-Related Adverse Events

5.3.1.1 General Management of Non-Hematological Toxicities

In general, prolonged (> 1 week) non-hematological Grade 2 adverse events that are assessed to be causally related to sitravatinib should be managed by dose reduction of sitravatinib. Exceptions, whereby patients can continue at the same dose, include lab abnormalities; nausea, vomiting or diarrhea that is manageable with supportive care; and any Grade 2 toxicity whose maximal severity is Grade 2 in NCI CTCAE, Version 5.0.

Non-hematological toxicities ≥ Grade 3 and considered to be sitravatinib-related should be managed with permanent discontinuation of sitravatinib as outlined in Table 4. For Grade 3 toxicities of nausea, vomiting, diarrhea, and lab abnormalities that are adequately managed by routine supportive care (such as anti-emetics, anti-diarrheals or electrolyte supplementation) and persist ≤72 hours, Grade 3 fatigue lasting ≤ 8 days, or Grade 3 amylase or lipase elevation, sitravatinib treatment may be interrupted until resolution of toxicity to ≤ Grade 1 or to baseline value and subsequently resumed at the same dose.

Table 4: Sitravatinib Dose Modifications – Non-Hematological Drug-Related Toxicities[1]

Toxicity	Treatment Delay	Dose Modification
Grade 1, or Grade 2 (≤ 1 week)	Investigator discretion[2]	
Prolonged Grade 2 (> 1 week)	Investigator discretion[2]	Decrease by 1 dose level[3]
Grade 3 or 4	Discontinue sitravatinib[2,4]	Discontinue sitravatinib[2,4]

1 Special cases for adverse events of special interest for sitravatinib are presented in Section 5.3.1.3.
2 The current OPDIVO® US Prescribing Information (USPI OPDIVO [nivolumab]) must be consulted to determine appropriate dose modifications for nivolumab.
3 Patients may instead continue at the same dose for the following prolonged Grade 2 adverse events:
   a. Grade 2 nausea, vomiting or diarrhea that is manageable with supportive care;
   b. Grade 2 electrolyte abnormality that is not clinically complicated and resolves spontaneously or with conventional medical treatment;
   c. Grade 2 fatigue;
   d. Grade 2 amylase or lipase elevation (that is not associated with symptoms or clinical manifestations of pancreatitis).
   e. Any Grade 2 adverse event whose maximal severity is Grade 2 in NCI CTCAE, Version 5.0.
4 Patients may instead hold sitravatinib until toxicity ≤ Grade 1 or returns to baseline and subsequently resume at the same dose in the following cases:
   a. Grade 3 nausea, vomiting or diarrhea that is manageable with supportive care and persists for ≤ 72 hours;
   b. Grade 3 electrolyte abnormality that is not clinically complicated and resolves spontaneously or with conventional medical treatment within 72 hours;
   c. Grade 3 fatigue that persists for ≤ 8 days;
   d. Grade 3 amylase or lipase elevation that is not associated with symptoms or clinical manifestations of pancreatitis.

5.3.1.2 General Management of Hematological Toxicities

Hematological toxicities are not a frequent cause of treatment interruption or discontinuation of sitravatinib treatment.

In general, prolonged (> 1 week) hematological Grade 2 toxicities that are considered to be causally related to sitravatinib should be managed by dose reduction of sitravatinib.

Observed ≥ Grade 3 hematological events that are considered to be causally related to sitravatinib should initially be managed using treatment interruption. In addition, dose reduction of sitravatinib should be implemented in the following cases:

- Grade 4 neutropenia persisting for ≥ 8 days; or
- Grade 3 thrombocytopenia with bleeding.

Conversely, permanent discontinuation of sitravatinib should be implemented in the following cases:

- Grade 3 or 4 febrile neutropenia; or
- Grade 4 thrombocytopenia of any duration.

5.3.1.3 Management of Selected Adverse Events

The following are guidelines for management of potential AEs more specific to treatment with sitravatinib or agents in the same class of cancer treatment.

5.3.1.3.1 Hypertension

Hypertension, including Grade 3 events, has been reported with sitravatinib. Dihydropyridine calcium channel blockers such as nifedipine, amlodipine, and nicardipine may be considered if anti-hypertensive therapy is required, and should be considered for patients with Grade 3 hypertension without clinically significant increases in blood pressure (BP) (see Table 5). On the other hand, in cases of Grade 3 hypertension with clinically significant increases in blood pressure (see Table 5), temporary suspension of sitravatinib dosing is recommended until blood pressure is controlled. Treatment with sitravatinib may resume at the same or a lower dose at the discretion of the Investigator. If significant hypertension recurs, options include change in medical management of the patient, reduction of sitravatinib dose, or discontinuation of study treatment, at the discretion of the Investigator. In the event of Grade 4 hypertension, sitravatinib should be permanently discontinued (see Table 5).

Table 5: Sitravatinib Dose Modification for Increased Blood Pressure

Toxicity	Treatment Delay	Dose Modification
Grade 1 or 2 hypertension	As per Table 4	
Grade 3 hypertension without clinically significant increases in BP as defined below	Investigator discretion. Consider anti-hypertensives per Section 5.3.1.3.1	
Grade 3 hypertension with clinically significant increases in BP *defined as* either an increase of ≥ 30 mmHg in systolic BP to ≥ 180 mmHg *or* increase of ≥ 20 mmHg in diastolic BP to ≥ 110 mmHg, confirmed with repeated testing after at least 5 minutes	Hold until ≤ Grade 2 or return to baseline	Investigator discretion
Grade 4 hypertension	Discontinue sitravatinib	Discontinue sitravatinib

5.3.1.3.2 Palmar-Plantar Erythrodysesthesia

Palmar plantar erythrodysesthesia (PPE) has been reported as a dose-limiting toxicity in the Phase 1 study of sitravatinib. Measures that can be taken to manage PPE include avoidance of exposure of hands and feet to hot water when washing dishes or bathing, or to other sources of heat, avoidance of activities that cause unnecessary force or friction (rubbing) on the hands or feet, avoiding contact with harsh chemicals such as cleaning products, use of tools or household items that result in pressure on the hands, such as garden tools, knives, and screwdrivers, and wearing of loose fitting, well-ventilated shoes and clothes. Treatment may include use of topical moisturizing agents, topical anesthetics or topical anti-inflammatory medications such as corticosteroid creams. In more severe cases, dose interruption and reduction may be warranted. In the event of Grade ≥ 3 palmar-plantar erythrodysesthesia, sitravatinib should be permanently discontinued.

5.3.1.3.3 Diarrhea

Immune-mediated etiology (e.g., immune-mediated colitis) should be considered (see Section 5.3.3.1 for management of immune-mediated diarrhea/colitis).

Diarrhea has been reported with sitravatinib treatment, though the mechanism remains unclear, as with other small molecule receptor TKIs. Patients should be counseled that diarrhea is a possible side effect and advised to take loperamide or a similar medication as needed if diarrhea develops. Any patients developing dehydration or clinically significant electrolyte abnormalities should interrupt treatment, but treatment may be restarted once diarrhea is controlled.

5.3.1.3.4 Hemorrhagic Events

The risk of hemorrhagic events with sitravatinib is unknown; however, such events have been reported with inhibitors of VEGFR. Patients with active hemoptysis or gastrointestinal bleeding should not take sitravatinib, and suspension of treatment is recommended for patients developing clinically significant bleeding. In the event of Grade ≥ 3 hemorrhage, sitravatinib should be permanently discontinued.

5.3.1.3.5 Thrombotic Events

Though thrombotic events (e.g., pulmonary embolism) have been reported with sitravatinib and with inhibitors of VEGFR, the risk of such events with sitravatinib is unknown. Precautions should be taken in patients with recent, clinically significant thrombotic events, and treatment should be discontinued in the event of Grade ≥ 2 thrombotic events including thrombosis, pulmonary embolism, myocardial infarction, cerebrovascular accident and thromboembolic event.

5.3.1.3.6 Thyroid Dysfunction Other than Immune-Mediated

Hypothyroidism and increases in thyroid-stimulating hormone (TSH) have been reported in patients taking sitravatinib. Patients diagnosed with hypothyroidism should be treated with thyroid replacement and may continue treatment with sitravatinib at the Investigator’s discretion.

5.3.1.3.7 Decreased Left Ventricular Ejection Fraction

Decreased LVEF has been reported with sitravatinib. In addition, decreases of LVEF to < 50% on-study were observed in patients undergoing scheduled multigated acquisition (MUGA) scans or echocardiograms. Discontinuation should be considered in the event of treatment-related, Grade ≥ 2 decreased ejection fraction and for patients requiring acute hospitalization for treatment of CHF.

5.3.1.3.8 Proteinuria

Although the risk with sitravatinib is unknown, proteinuria has been described with other inhibitors of the VEGFR pathway. Patients who develop ≥ 2+ proteinuria should undergo 24-hour urine collection for assessment of urine protein; treatment with sitravatinib should be discontinued in the presence of ≥ 2 g of proteinuria/24 hours and may restart when protein levels decrease to less than 2 g/24 hours. Patients who develop nephrotic syndrome should be withdrawn from treatment with sitravatinib.

5.3.2 Nivolumab-Related Adverse Events

Refer to the OPDIVO® US Prescribing Information (USPI OPDIVO [nivolumab]) Section 2.10 and Section 5 for guidance concerning management of AEs, including immune-related AEs, during treatment with nivolumab.

5.3.3 Management in Event of Immune-Related Adverse Events

Sitravatinib has not been associated with irAEs. However, the potential exists for sitravatinib to contribute to irAEs associated with nivolumab treatment. In the event of a Grade 2 irAE during study treatment, administration of sitravatinib and nivolumab should be interrupted until the event stabilizes to Grade ≤ 1 (consistent with guidance provided in the OPDIVO® US Prescribing Information (USPI OPDIVO [nivolumab])). At the time of resumption of sitravatinib dosing, a dose reduction may be implemented at the discretion of the Investigator.

In general, patients should permanently discontinue nivolumab in the presence of any Grade 3 or 4 irAEs. Sitravatinib may be resumed at the same or lower dose at the discretion of the Investigator until the event stabilizes to Grade ≤ 1.

5.3.3.1 Diarrhea/Colitis

The management of diarrhea should be guided by clinical judgment and an assessment of the most likely causative etiology, with special consideration given to the potential for immune-mediated colitis. The presence of abdominal pain, mucus or blood in the stool or peritoneal signs should raise the index of suspicion for immune-mediated colitis, as these features are generally not observed with sitravatinib treatment-associated diarrhea. The diarrhea observed with sitravatinib generally improves within several days of interrupting study medication, with close observation may help establish the most likely causality. However, if any features of the clinical presentation, including timing of presentation, failure to improve with dose interruption, laboratory or radiologic tests suggests the presence of immune-mediated colitis, all study medications should be withheld and treatment with immuno-suppressive therapy initiated as detailed in the OPDIVO® US Prescribing Information (USPI OPDIVO [nivolumab]).

5.3.3.2 Increased Transaminases

The management of increases in AST and ALT should be guided by the clinical judgment of the Investigator, including an assessment of the most likely causative etiology, with special consideration given to the potential for immune-mediated hepatitis. Increased transaminases should be evaluated to determine whether confounding factors exist, such as viral infection, metastatic lesions or biliary obstruction.

For cases where transaminase increases are <u>not</u> likely to be immune-mediated, treatment management decisions should be made using Investigator discretion in consideration of clinical factors. Recommended treatment modifications for sitravatinib are provided in Table 6. However, if any features of the clinical presentation, including timing of presentation, failure to improve with dose interruption, laboratory or radiologic tests suggests the presence of immune-mediated hepatitis, all study medications should be withheld and treatment with immuno-suppressive therapy initiated as detailed in the OPDIVO® US Prescribing Information (USPI OPDIVO [nivolumab]).

Table 6: Sitravatinib Dose Modification for Increased Hepatic Transaminase

Toxicity	Treatment Delay	Dose Modification
Grade 1 (> ULN – 3.0 × ULN)	May be implemented at Investigator and patient discretion	
Grade 2 (> 3.0 – 5.0 × ULN)	Not required[1]	Decrease by 1 dose level[1]
Grade 3/4 (> 5.0 × ULN)	Discontinue sitravatinib[1]	Discontinue sitravatinib[1]

1 The current OPDIVO® US Prescribing Information (USPI OPDIVO [nivolumab]) (and Section 5.3.3, in case the event is deemed to be an irAE) must be consulted to determine appropriate dose modifications for nivolumab.

5.3.3.3 Management of Hy's Law Cases

In the event a patient develops concurrent increase in AST and/or ALT ≥ 3 × ULN, bilirubin ≥ 2 × ULN but without concurrent increases in alkaline phosphatase (i.e., alkaline phosphatase < 2 × ULN), that is not attributable to liver metastases or biliary obstruction, sitravatinib and nivolumab should be permanently discontinued and steroids administered.

5.4 Medication Error

Medication errors may involve patient exposure to a wrong study drug, at a wrong dosing frequency, or at a wrong dose level (e.g., a dose that is not planned in the study). Medication errors occurring during the conduct of this study will be documented as AEs (regardless of whether clinical signs or symptoms are observed) and if serious consequences are observed, will be reported on SAE forms. In all cases of medication error, the sponsor should be notified immediately.

There is currently no specific treatment in the event of an overdose of sitravatinib or nivolumab. The Investigator will use clinical judgment to treat any overdose.

5.5 Concomitant Therapies

5.5.1 Concomitant Medication(s)

Concomitant medications must be locally approved and used at doses and regimens that are considered standard-of-care for the treated indication. Treatment for co-morbidities, disease signs and symptoms and treatment-emergent adverse events (TEAEs) should be provided as necessary in the judgment of the Investigator. Patients may continue to use any ongoing medications not prohibited by the inclusion/exclusion criteria or treatment plan.

Antibiotics and analgesics: These drugs should be used as needed. Patients with neutropenic fever or infection should be treated promptly (therapeutic colony-stimulating factors should be used in accordance with ASCO guidelines).

Anti-diarrheals: In general, patients should be counseled that diarrhea is a possible side effect of the study treatments and advised to take loperamide or a similar medication as needed if diarrhea develops.

Anti-emetics: Patients may be premedicated for nausea and vomiting. Recommended anti-emetic agents include granisetron 1 mg as premedication, and then granisetron and/or prochlorperazine as needed.

Gastric Acid Medications: Solubility of sitravatinib is maximal at low pH. Proton pump inhibitors and H_2-antagonists should be avoided during treatment on study but are not exclusionary. Switch from use of proton pump inhibitors or H_2 antagonists to use of antacids is preferred. Use of antacids should be avoided 4 hours before and 2 hours after administration of sitravatinib.

Immunosuppressive Medications: Use of immunosuppressive mediations should be limited to the extent possible to allow testing of the immune-stimulatory mechanisms proposed in this clinical trial. Immunosuppressive medications should be used as needed to manage irAEs and the extent required to manage comorbidities and symptoms of disease.

Vaccines: Live attenuated vaccines within 28 days of nivolumab dosing are to be avoided. Inactivated vaccines, such as the injectable influenza vaccine, are permitted.

Medications with potential for drug-drug interactions: Full details of the medications to be avoided or used with caution during study drug treatment are outlined in Appendix 2.

Sitravatinib inhibits P-gp and BCRP transporters at high concentrations and, as a result, sensitive substrates and substrates with narrow therapeutic index for P-gp and BCRP transporters should be used with caution during study drug treatment. Similarly, preclinical results suggest that multiple enzymes, including CYP 1A2, 2A6, 2B6, 2C8, 2C9, 2D6, 2E1, and 3A4 are involved in the metabolism of sitravatinib with a low risk of one enzyme contributing to metabolism in a disproportionate manner. Therefore, sensitive substrates and substrates with narrow therapeutic index for indicated CYP enzymes should be used with caution during study drug treatment. Finally, QTc prolongation with sitravatinib is under evaluation, though no signal has been detected from the ongoing sitravatinib clinical trials. Prior to results of such evaluation, drugs with a known risk of torsades de pointes should be avoided during study drug treatment, whereas drugs with conditional risk of torsades de pointes may be used, but with caution during study drug treatment.

5.5.2 Concomitant Surgery or Radiation Therapy

The study involves a planned surgical intervention in the form of partial or radical nephrectomy following the combination treatment.

The use of any radiation or earlier-than-planned surgery to manage cancer lesions during study drug treatment can jeopardize assessment of the primary objective of the study, evaluation of disease status. Therefore, the use of these treatments during study drug treatment is discouraged. However, they may be used in cases where these treatments are deemed medically necessary. In these cases, patients should discontinue study drug treatment, if possible at least 2 days prior to the treatment. These patients will not resume study drug treatment. If patients receive a medically necessary, earlier-than-planned surgery involving tumor tissue collection from the patient’s renal tumor (e.g., earlier than planned partial or radical nephrectomy), efforts should be made to collect tumor tissue samples from the surgical sample. Patients with earlier-than-planned surgery or radiation while on study should proceed to their EOS visit (at least 28 days after last dose) without resuming dosing.

5.5.3 Other Anticancer or Experimental Therapy

Use of approved or investigational anticancer treatment will not be permitted during the study treatment period, including chemotherapy, biological response modifiers, hormone therapies or immunotherapy. No other investigational drug may be used during treatment on this protocol. Concurrent participation in another therapeutic clinical trial is not allowed.

6 STUDY ASSESSMENTS

6.1 Screening

Voluntary, written, dated, and signed informed consent must be obtained before any study specific procedures are performed. Patients who completed the informed consent process but did not enroll on the study will be considered as screen failures. Limited information will be recorded in the CRF for these patients.

6.2 Study Period

For details on procedures during the study, see Schedule of Assessments (Table 1).

6.3 End of Study (EOS) Assessment

All patients will be followed for AEs and SAEs for at least 28 days after the last dose of study drug treatment or surgery, whichever occurs last. See the Schedule of Assessments (Table 1) for evaluations to be performed at the EOS visit.

6.4 Long-Term Follow-up

Patient disease-free survival may be followed by review of medical records or telephone contact with the patient or treating healthcare professional approximately every 6 months from EOS visit for up to 3 years or more until death, disease recurrence or loss to follow-up.

6.5 Patient Discontinuation/Withdrawal

Patients may discontinue from study treatment or from the study at any time at their own request, or they may be discontinued at any time at the discretion of the Investigator or Sponsor for safety, behavioral reasons, or the inability of the patient to comply with the protocol required schedule of study visits or procedures at a given study site.

Criteria that may be used to discontinue patients from receipt of study medication will include, but will not be limited to:

- Objective disease progression as determined by the Investigator;
- Global deterioration of health status requiring discontinuation;
- Adverse event (AE) (see Section 5.3);
- Significant protocol violation;
- Loss to follow-up;
- Refusal for further treatment;
- Study termination by Sponsor;
- Death.

If a patient does not return for a scheduled visit, every effort should be made to contact the patient. At least 2 attempts should be made to contact the patient, and each attempt should be recorded in the source documents. In any circumstance, every effort should be made to document patient outcome, if possible. The Investigator should inquire about the reason for withdrawal, request that the patient returns for a final visit, and if applicable, follow-up with the patient regarding any unresolved AEs.

If the patient withdraws consent, no further evaluations should be performed, and no additional data should be collected. The Sponsor may retain and continue to use any data collected before the withdrawal.

7 PROCEDURES

Every effort should be made to ensure that the protocol required tests and procedures are completed as described. However, it is anticipated that there may be circumstances

outside of the control of the Investigator that may make it unfeasible to perform a protocol-specified assessment. In these cases, the Investigator will take all steps necessary to ensure the safety and well-being of the patient. When a protocol required test cannot be performed, the Investigator will document in the source document and CRF the reason and any corrective and preventive actions which he/she has taken to ensure that normal processes are adhered to as soon as possible. The study team will be informed of these incidents in a timely fashion.

7.1 Efficacy

All patients enrolled in the study are to be evaluated for disease activity as outlined in the Schedule of Assessments (Table 1). All known and suspected sites of disease should be evaluated at each assessment. Screening/baseline tumor assessments should include CT or X-ray (radiography) of the chest, and CT or MRI of abdomen, and, if clinically indicated, whole body bone scan and CT with contrast or MRI of the brain and evaluation of any superficial lesions. The subsequent, on-study disease assessment will include imaging of all known and suspected sites of disease and obtained in the preoperative setting, namely at the Preoperative Rebaseline visit (which should generally occur within 3 days of the planned partial or radical nephrectomy). The allowable windows for assessments are 4 weeks prior to first study treatment for screening/baseline assessments and within 1 week of the planned partial or radical nephrectomy for the on-study disease assessment.

CT scans should be performed with contrast agents unless contraindicated for medical reasons. If IV contrast is medically contraindicated, the imaging modality to be used (either CT without contrast or MRI) should be the modality which best evaluates the disease, and the choice should be determined by the Investigator in conjunction with the local radiologist. Depending on the adequacy for evaluation of disease, a combination of CT without contrast and MRI should most often be used. CT without contrast is preferred for evaluation of lesions in lung parenchyma. Chest X-ray is also acceptable. MRI is not adequate for evaluation of lung parenchyma but should also be performed to evaluate all other aspects of the chest. MRI of the abdomen should substitute for CT with contrast unless the method does not adequately depict the individual's disease, in which case CT without contrast is preferred.

Disease response will be assessed in accordance with RECIST 1.1 (Eisenhauer-2009). Appendix 3 provides guidance in using the response criteria. The appendix includes a clarification that based on the single on-study disease assessment, RECIST 1.1 will be used in the determination of the single point in time objective response to treatment. Because only one post-treatment imaging assessment is expected, objective responses will not be confirmed by repeated imaging.

The Investigator's assessment of disease response and progression will be the basis for patient management.

Secondary efficacy endpoint will include long-term follow-up for Disease Free-Survival (DFS) and will be collected by review of medical records or telephone contact with the

patient or treating healthcare professional approximately every 6 months from EOS visit for up to 3 years or more until death, disease recurrence or loss to follow-up. Disease recurrence will be based on imaging assessments performed off study per standard of care after nephrectomy. After sufficient data for the primary endpoint is collected in this study, the electronic study database may be locked, and the collection of the long-term survival status may be reported through a different mechanism (rather than using the electronic database).

7.2 Safety Assessments

7.2.1 Medical History

Signs and symptoms of the patient's cancer diagnosis and/or comorbidities present at baseline will be recorded in the CRF as AEs beginning on D1 of study treatment and onward throughout the study. The actual date of onset should be recorded in all cases.

7.2.2 Physical Examination and Vital Signs

A physical examination including all major body systems is mandated at Screening and EOS Visits only. During study treatment, symptom directed physical examinations will be performed.

Vital signs to be assessed include weight, body temperature, blood pressure, and pulse rate. Height will be recorded at screening only. On days when both vital signs and PK sampling are scheduled, the vital signs should be assessed prior to blood sampling.

Clinically significant findings noted during screening will be reflected on the medical history CRF, while those noted during study treatment will be collected on the AE CRFs.

7.2.3 Laboratory Safety Assessments

Laboratory safety assessments for which data will be collected in this study will include hematology, coagulation, and chemistry parameters presented in Table 7.

Laboratory tests will be drawn at the time points described in the Schedule of Assessments (Table 1) and analyzed at local laboratories. Additional laboratory tests may be performed per standard of care, at the Investigator's discretion for the purpose of planning treatment administration, dose modification, following AEs, or as clinically indicated.

Table 7: Laboratory Safety Parameters

Hematology Panel	Blood Chemistry Panel
Hemoglobin	Aspartate aminotransferase (AST)
Platelet count	Alanine aminotransferase (ALT)
White blood cell count (WBC)	Alkaline phosphatase

Hematology Panel	Blood Chemistry Panel
Neutrophil count	Total bilirubin
Lymphocyte count	Lipase
	Amylase
**Coagulation**	Sodium
International normalized ratio (INR)	Potassium
Partial thromboplastin time (PTT)	Chloride
	Bicarbonate
**Urinalysis (dip stick)**	Blood urea nitrogen (BUN)
Blood	Creatinine
Protein	Albumin
	Total calcium
**Thyroid Function Test**	Magnesium
Thyroid-stimulating hormone (TSH)	Phosphate
	Uric acid

Pregnancy Testing: For patients of childbearing potential, a serum or urine pregnancy test will be performed at screening. Pregnancy tests will also be done whenever pregnancy is suspected during the study. Additional pregnancy testing may be necessary if required by local practices or regulations.

7.2.4 Electrocardiogram (ECG)

Single and triplicate ECGs are to be performed as outlined in the Schedule of Assessments (Table 1 and Table 2). It is preferable that the machine used has a capacity to calculate standard intervals automatically. In addition, QTc will be calculated manually using Fridericia's formula. Assessments reported by automated read as QTc prolongation are advised to be over-read by a cardiologist to ensure accuracy of interpretation.

7.2.5 Echocardiogram (ECHO)

Echocardiograms will be performed at screening, and thereafter as in the Schedule of Assessments (Table 1).

7.3 Laboratory Studies

Full details on sample collection, processing, storage and shipment are presented in the Study Laboratory Manual.

7.3.1 Pharmacokinetic Evaluation

The PK of sitravatinib will be determined using blood samples collected at specified time points prior to and following study treatment dosing. Every effort will be made to collect these PK samples at the exact nominal times relative to dosing. A variation window is allowed for each time point as outlined in Table 2. The actual time of each sample collection will be recorded on the source document and CRF.

All plasma samples will be stored frozen and shipped on dry ice according to instructions provided. Analysis of samples will be performed using specific validated bioanalytical methods. Full details on sample collection, processing, storage and shipment will be provided in the Study Laboratory Manual.

7.3.2 Pharmacodynamic Evaluation in Blood

Immune effects will be evaluated through circulating prior to and during and following treatment as outlined in Table 2. Blood samples will be collected for the assessment of parameters that may include, but are not limited to, quantification of circulating immune cell populations by flow cytometry including T-cell subpopulations (CD4+, CD4+/Ki67+, CD8+, CD8+/Ki67+ and Tregs), NK cells, and MDSCs; blood levels of selected cytokines and chemokines including IFN-γ, IL-1ß, IL-6, IL-8, IL-12, IL-18 and CXCL10 (IP-10); and DNA sequencing to assess T-cell clonality. Full details on sample collection, processing, storage and shipment will be provided in the Study Laboratory Manual.

7.3.3 Pharmacodynamic Evaluation in Tumor Tissue

Tumor tissue will be collected from initial and mid-study biopsies and surgical sample. Fresh tumor biopsies are to be collected on study; archival tissue will not be accepted as an alternative. Tumor tissue will be collected and used to assess immune effects through tissue biomarkers prior to and during and following treatment. Parameters may include, but are not limited to, quantification of tissue immune cell populations by flow cytometry including T-cell subpopulations (CD8+, CD8+/Ki67+ and Tregs), NK cells, MDSCs, and macrophages; immunohistochemistry and/or immunofluorescence for assessment of PD-L1 status and characterization of immune cell populations; tumor gene expression analysis; and DNA sequencing to assess T-cell clonality. In addition, next generation sequencing (NGS) analyses for tumor mutations and tumor mutation burden may be assessed using tumor tissue from the surgical sample. For patients whose tumor tissue has previously been tested using NGS, presence of specific tumor gene mutations and estimation of total mutation burden will be collected in the CRF.

Samples should be of acceptable quality and quantity for analysis. Samples should be collected via a core needle of 18 gauge or larger with sufficient passes or be collected by an incisional or excisional tumor biopsy. Samples from fine needle aspirates (FNA) are not appropriate for study evaluations. Further guidance on sample preparation, handling and submission can be found in the Study Laboratory Manual.

7.3.4 Circulating and Tissue Tumor DNA

NGS analyses for tumor mutations and tumor mutation burden will be assessed at using baseline circulating tumor cell DNA (ctDNA) blood samples outlined in Table 2 and using tumor tissue from the surgical sample as outlined in Table 1. At each ctDNA time point, blood samples will be collected into two 10 mL Streck brand Cell-Free DNA Blood Collection tubes allowing shipping and stability at ambient temperatures. Tissue samples should be of acceptable quality and quantity for analysis. Full details on sample collection, processing, storage and shipment will be provided in the Study Laboratory Manual.

For patients whose tumor tissue or blood has previously been tested using NGS, presence of specific tumor gene mutations and estimation of total mutation burden will be collected in the CRF.

8 ADVERSE EVENT REPORTING

8.1 Sponsor Medical Monitor Personnel

The contact information for the sponsor's Medical Monitor personnel for this trial is available in the study contact list located in the Site Operations Manual.

8.2 Adverse Events

An AE is any reaction, side effect or other undesirable medical event that occurs during participation in a clinical trial, regardless of treatment group or suspected causal relationship to study treatment. Assessment of AEs will include type, incidence, severity (graded by the National Cancer Institute [NCI] Common Terminology Criteria for Adverse Events [CTCAE, Version 5.0]), timing, seriousness, and relatedness to study treatment. A treatment-emergent adverse event (TEAE) is an AE that occurs after the first dose of any study treatment or any preexisting condition that increases in severity after the first dose of study treatment.

All observed or volunteered AEs will be recorded in source documents and reported in the CRF. The best available medical terminology should be used to describe AEs in source documents and CRFs. Terms describing the diagnosis are preferred over individual signs and symptoms of the diagnosis. If determination of the diagnosis is delayed, record signs and symptoms and add the diagnosis as an additional AE when available; follow all recorded AEs to resolution. The actual date of onset should be recorded in all cases. Ongoing AEs that change in attribution or severity should have the date of change entered as the “end date” and a new AE record should be opened with the changed details. Examples of AEs include but are not limited to:

- Signs or symptoms of co-morbidity, illness, or toxicity of study treatment;
- Signs or symptoms of worsening malignancy under study (disease progression assessed by measurement of malignant lesions should not be reported as an AE).

- Laboratory abnormalities (see Section 8.2.1 for guidance for reporting in CRF);
- Hypersensitivity;
- Drug abuse, dependency, overdose, withdrawal or misuse;
- Signs or symptoms of drug interactions;
- Extravasation;
- Exposure during pregnancy or via breastfeeding;
- Medication error; or
- Occupational exposure.

8.2.1 Laboratory Abnormalities

An abnormal laboratory test results should be reported as an AE in the CRF only if it is associated with one or more of the following:

- Clinical symptoms;
- Requires additional tests (beyond repeats), treatment or intervention;
- Results in change in study treatment dosing;
- Requires discontinuation from study treatment; and/or
- Considered by the Investigator or Sponsor to be an AE.

8.2.1.1 Hy's Law

Hepatic function abnormality defined by an increase in AST and/or ALT to $\geq 3 \times$ ULN concurrent with an increase in total bilirubin to $\geq 2 \times$ ULN but without increase in alkaline phosphatase (i.e., alkaline phosphatase $< 2 \times$ ULN) meets the criteria for Hy's Law and raises the concern for drug-induced liver injury when no other cause of the abnormal laboratory results is identified. Follow-up investigations and inquiries will be initiated promptly by the investigational site to determine whether the findings are reproducible and/or whether there is objective evidence that clearly supports causation by a disease (e.g., cholelithiasis and bile duct obstruction with distended gallbladder) or an agent other than the investigational product.

Cases meeting Hy's Law should be reported as SAEs. Both study drugs should be permanently discontinued for a Hy's Law case and steroids administered.

8.2.2 Severity Assessment

AEs occurring during this study will be graded in accordance with the NCI CTCAE, Version 5.0. Documentation of AE grading in the source documents and CRF should be consistent with provided definitions.

8.2.3 Causality

For each AE, the Investigator should determine and document whether there exists a reasonable possibility that the study treatment caused or contributed to the AE. The Investigator's assessment should be recorded in the source document. The CRF will provide the options for attribution to study treatment as "related" and "not related." If the Investigator's causality assessment is "unknown but not related to investigational product," this should be recorded in the CRF as "not related." If the Investigator does not know whether or not the study treatment is causally-related to the event, reporting for study purposes will be as "related" to study treatment.

Collection of causal relationship for AEs associated with study procedures (e.g., tumor biopsy) is provided for separately in the CRF.

8.3 Serious Adverse Events

8.3.1 Definition of a Serious Adverse Event

An SAE is any event that meets any of the following criteria:

- Results in death;
- Is life-threatening (immediate risk of death);
- Requires inpatient hospitalization or prolongation of existing hospitalization;
- Results in persistent or significant disability/permanent damage (substantial disruption of the ability to conduct normal life functions);
- Results in congenital anomaly/birth defect.
- Other: Important medical events that may not result in death, be life-threatening, or require hospitalization, may be considered an SAE when, based upon appropriate medical judgment, they may jeopardize the patient and may require medical or surgical intervention to prevent one of the outcomes listed in this definition. Examples of such events are:
  - Intensive treatment in an emergency room or at home for allergic bronchospasm;
  - Blood dyscrasias or convulsions that do not result in inpatient hospitalization;

– Development of drug dependency or drug abuse.

Progression of the malignancy under study, including any signs or symptoms of progression that may require hospitalization, should not be reported as an SAE unless the outcome is fatal within the safety reporting period.

Definition of Terms

Life-threatening: An AE is life threatening if the patient was at immediate risk of death from the event as it occurred; i.e., it does not include a reaction that if it had occurred in a more serious form might have caused death. For example, drug-induced hepatitis that resolved without evidence of hepatic failure would not be considered life-threatening even though drug-induced hepatitis can be fatal.

Hospitalization: In general, hospitalization signifies that the patient has been detained (usually involving at least an overnight stay) at the hospital or emergency ward for observation and/or treatment that would not have been appropriate in the physician's office or outpatient setting. When in doubt as to whether 'hospitalization' occurred or was necessary, the AE should be considered serious. Hospitalization for elective surgery or routine clinical procedures that are not the result of AE (e.g., elective surgery for a pre-existing condition that has not worsened) need not be considered AEs or SAEs. If anything untoward is reported during the procedure, that occurrence must be reported as an AE, either 'serious' or 'non-serious' according to the usual criteria.

Disability/permanent damage: An AE is disabling or caused permanent damage if it resulted in a substantial disruption of a person's ability to conduct normal life functions, e.g., a significant, persistent or permanent change, impairment, damage or disruption in body function/structure, physical activities and/or quality of life.

Adverse Event of Special Interest (AESI): AESIs are of scientific and medical interest specific to understanding of the Investigational Product and may require close monitoring and rapid communication by the Investigator to the sponsor. An AESI may be serious or non-serious. The rapid reporting of AESIs allows ongoing surveillance of these events in order to characterize and understand them in association with the use of this investigational product.

Immune-related Adverse Events (irAE): An irAE is defined as an AE that is associated with drug exposure and is consistent with an immune-mediated mechanism of action and where there is no clear alternate etiology. Serologic, immunologic, and histologic (biopsy) data, as appropriate, should be used to support an irAE diagnosis. Appropriate efforts should be made to rule out neoplastic, infectious, metabolic, toxin, or other etiologic causes of the irAE.

8.3.2 Exposure During Pregnancy

Exposure during pregnancy (i.e., exposure in-utero [EIU]) may occur in a female study participant, the female partner of a male study participant or study site personnel working with the investigational product (e.g., occupational exposure) if:

- A female becomes or is found to be pregnant during treatment or within 6 months after discontinuing treatment or having been directly exposed to the investigational product;
- A male is exposed to the investigational product prior to or around the time of conception or during the pregnancy of his partner.

If exposure in-utero occurs, the Investigator must submit an SAE form and an EIU Supplemental Form within 24 hours of awareness of the exposure, regardless of whether an AE or SAE has occurred.

In the event of pregnancy in a female study participant, if the pregnancy is continued, study treatment will be immediately discontinued.

In the event of exposure of the pregnant partner of a male study participant, the study participant should be asked to deliver an EIU Pregnant Partner Release of Information Form to his partner. The Investigator must document on the EIU Form that the patient was given this letter to provide to his partner.

Follow-up to obtain pregnancy outcome information is to be conducted for all EIU reports. In the case of a live birth, the health of the neonate should be assessed at the time of birth and for up to 3 months after birth. Further follow-up of birth outcomes will be handled on a case-by-case basis (e.g., follow-up on preterm infants to identify developmental delays). In the event the pregnancy is terminated, the reason(s) for termination should be reported and, if clinically possible, the structural integrity of the terminated fetus should be assessed by gross visual inspection.

If the outcome of the pregnancy meets the criteria for an SAE (i.e., ectopic pregnancy, spontaneous abortion, intrauterine fetal demise, neonatal death, or congenital anomaly), an SAE report should be submitted to the Sponsor.

8.4 Reporting of Serious Adverse Events and Adverse Events

8.4.1 Reporting Period

The active reporting period for SAEs begins from the time that the patient provides informed consent (i.e., prior to undergoing any study-specific procedure or assessment) and continues until at least 28 days after last administration of study treatment or surgery, whichever occurs last. All SAEs ongoing 28 days after the last dose should be followed until all SAEs have resolved or stabilized to a chronic condition, whichever is later. If a patient begins a subsequent systemic anticancer therapy, the reporting period for new SAEs ends at the time the new treatment is started. Death must be reported if it occurs

during the active reporting period for SAEs regardless of whether a subsequent systemic anticancer therapy is administered. SAEs occurring to a patient after the active reporting period has ended should be reported to the Sponsor if the Investigator becomes aware of them and if the Investigator assesses at least a reasonable possibility of being related to drug. These SAEs should be followed until resolved or stabilized to a chronic condition.

The reporting period for non-serious AEs begins from the day of first dose of study treatment and continues until at least 28 days after last administration of study dug treatment or surgery, whichever occurs last. If a patient begins a subsequent systemic anticancer therapy, the AE reporting period ends at the time the new treatment is started.

8.4.2 Reporting Requirements

All SAEs must be reported within 24 hours of Investigator/site knowledge of the event, irrespective of the extent of available AE information, by faxing the SAE report to the Sponsor's pharmacovigilance representative designated in the Site Operations Manual. The 24-hour timeframe also applies to additional new information (follow-up) on previously forwarded SAE reports and to the initial and follow-up reporting of exposure during pregnancy and exposure via breastfeeding. The need for an expedited report to regulatory authorities will be determined by the Sponsor and necessary reporting will be performed by the Sponsor. The Sponsor will notify study Investigators of all Suspected, Unexpected (as judged against the Investigator Brochure) Serious Adverse Reaction (SUSAR) reports. The Investigator is responsible for reporting all SUSARs to the IRB/EC/REB.

All AE (including SAEs) must be documented in source documents and reported in the CRF. Please note that the CRF and SAE report forms may collect information in somewhat different formats. Where the requested data overlap in different formats, the information should be consistent between the two forms.

After sufficient data for the primary endpoint is collected in this study, the electronic study database may be locked, and the collection of the long-term survival status may be recorded through a different mechanism (rather than using the electronic database).

9 STATISTICS

Detailed methodology for summary and statistical analyses of the data collected in this study will be documented in a Statistical Analysis Plan (SAP), which will be maintained by the Sponsor. The SAP may modify the plans outlined in the protocol; however, any major modifications of the primary endpoint definition and/or its analysis will also be reflected in a protocol amendment.

9.1 Statistical Design

9.1.1 Hypothesis and Sample Size

With currently available treatments, the percentage of patients with a point in time objective response prior to surgery is assumed to be 5% (p0); thus, this rate is considered uninteresting. The target percentage of patients with a point in time objective response prior to surgery using sitravatinib and nivolumab in this study is assumed to be 30% (p1). Controlling for a Type 1 error (α) of 0.05, and using an exact test (two-sided), with 18 clinical activity evaluable patients, we have 80% power to rule out a percentage of patients with a point in time objective response prior to surgery of 5% assuming percentage of patients with a point in time objective response prior to surgery of 30%.

Assuming a non-evaluable rate between 25%-30% from enrolled patients to clinical activity evaluable patients, the study will enroll approximately 25 patients in order to get 18 clinical activity evaluable patients. Additional subjects may be enrolled for a total of 12 subjects with complete tumor tissue collection (collected from initial and mid-study biopsies and surgical sample).

9.1.2 Dose De-escalation Plan

The Modified Toxicity Probability Interval (mTPI) method (Ji-2010) will be used to set rules on a dose de-escalation plan to monitor and limit toxicity of the combination regimen in the neoadjuvant setting. The first assessment will occur after the first 6 patients have completed 3 weeks on therapy, or earlier if 2 or more patients with toxicity are suspected. Subsequent assessments will occur at intervals that include up to 6 additional patients completing the observation period. Assuming a maximum toxicity level of no more than 20% at the tolerated dose, the dose will be decreased if more than 2 patients with toxicities are observed in the first 6 patients (Refer to Appendix 4 for the dose de-escalation table). For the purposes of decision making for dose de-escalation, patients with the following treatment-related adverse events will be used to determine the number of patients having toxicity:

1. Non-hematologic Grade 4 adverse event.
2. Non-hematologic Grade 3 adverse event with the following exceptions:
    a. Grade 3 nausea, vomiting or diarrhea that is manageable with supportive care and persists for ≤ 72 hours;
    b. Grade 3 electrolyte abnormality that is not clinically complicated and resolves spontaneously or with conventional medical treatment within 72 hours;
    c. Grade 3 fatigue that persists for ≤ 8 days;
    d. Grade 3 amylase or lipase elevation that is not associated with symptoms or clinical manifestations of pancreatitis.

3. Surgical delay of greater than 2 weeks and due to toxicity.

In the proposed plan, there is no dose escalation, and surgical delay will be assessed only for those patients who have already undergone surgery.

9.2 Data Handling

Listings of all patient data will be prepared. Data summaries will be presented in tabular and/or graphical format and summarized descriptively, where appropriate. Further details of planned analyses will be described in the SAP.

For all variables, only the observed data from patients will be used in the statistical analyses; there is no plan to estimate missing data. Patients without a valid clinical response assessment will be assigned a best overall response of not evaluable (NE). Data from patients who are lost to follow-up or have missing observations before reaching an endpoint in any of the time-to-event analyses will be treated as censored with specific rules defined in the SAP.

9.3 Analysis Populations

9.3.1 Clinical Activity Evaluable Population

The Clinical Activity Evaluable population will include patients who receive at least one dose of each study treatment drug and who have their on-study disease assessment prior to surgery. Patients who discontinue treatment due to AEs or withdrawal of consent prior to their on-study disease assessment will not be included in the clinical activity evaluable population. This population will be used to present tumor responses.

9.3.2 Safety Population

The Safety population is defined as all patients who received at least 1 dose of either sitravatinib or nivolumab. The Safety population will be used for all safety analyses.

9.3.3 Pharmacokinetic Evaluable Population

The Pharmacokinetic Evaluable population will consist of all patients who receive treatment with sitravatinib and have sufficient concentration-time data to permit calculation of PK parameters for sitravatinib. For patients who are noncompliant with respect to administration of sitravatinib, or for patients with incomplete data, a decision as to their inclusion in the analysis will be made on a case-by-case basis.

9.3.4 Pharmacodynamic Evaluable Population

The Pharmacodynamic Evaluable population will consist of all patients who receive at least 1 dose of either sitravatinib or nivolumab for whom sufficient pharmacodynamic data are available.

9.4 Efficacy Endpoint Definitions and Analyses

9.4.1 Percentage of Patients Achieving a Point in Time Objective Response (either CR or PR) Prior to Surgery

The primary endpoint of this study is percentage of patients with ccRCC achieving a point in time objective response (either CR or PR) prior to surgery with the combination treatment of sitravatinib and nivolumab. Single point in time objective response to treatment at the Preoperative Rebaseline visit will be categorized in accordance with RECIST 1.1 (Appendix 3).

Descriptive statistics (frequency and percentage) for CR, PR, and percentage of patients with ccRCC achieving a point in time objective response (either CR or PR) prior to surgery will be presented. The exact 95% CI of these response rates will be calculated. An exact test for single proportion (two-sided α=5%) will be performed to test H0: percentage of patients with ccRCC achieving a point in time objective response (either CR or PR) prior to surgery ≤ 5% against H1: percentage of patients with ccRCC achieving a point in time objective response (either CR or PR) prior to surgery > 5%. Other details will be described in the SAP.

Additionally, information regarding pathologic complete response (pCR) will be collected in the CRF and will be descriptively summarized. The determination of pCR to detect residual disease in kidney and regional lymph nodes will be based on the pathology examination of the surgical sample. pCR is defined as the absence of residual invasive disease on evaluation of the complete resected renal specimen and all sampled regional lymph nodes following completion of neoadjuvant systemic therapy.

9.4.2 Disease-Free Survival (DFS)

Patient survival may be followed by review of medical records or telephone contact with the patient or treating healthcare professional approximately every 6 months from EOS visit for up to 3 years or more until death, disease recurrence or loss to follow-up. Disease recurrence will be based on imaging assessments performed off study per standard of care after nephrectomy. After sufficient data for the primary endpoint is collected in this study, the electronic study database may be locked, and the collection of the long-term survival status may be recorded through a different mechanism (rather than using the electronic database).

DFS is defined as the time from date of surgery to disease recurrence or death whichever occurs first. For patients without disease recurrence or death (including those lost to follow-up), censoring for the DFS endpoint will be done using the date of the last follow-up or the date of surgery (with duration of 1 day), whichever occurs later. DFS will be analyzed using Kaplan-Meier methods.

9.5 Safety Data Presentations and Summaries

9.5.1 Prior and Concomitant Medications

Collected prior and concomitant medications will be coded using the WHO medical dictionary; patients who received these medications will be listed and summarized.

9.5.2 Adverse Events

AEs will be classified using the medical dictionary for regulatory activities (MedDRA) classification system. Listings will include the verbatim term, preferred term, and system organ class (SOC). The number of patients with treatment emergent AEs and the incidence of TEAEs by SOC and preferred term will be summarized. TEAEs will be summarized by maximum intensity and relationship to study therapy. Separate summaries will be provided for TEAEs, TESAEs, treatment-related AEs, treatment-related SAEs, and other significant AEs (e.g., AEs leading to study discontinuation).

9.5.3 Clinical and Laboratory Assessments

Clinical and laboratory assessments include clinical laboratory tests (hematology, coagulation, and chemistry), vital signs, and 12-lead ECGs.

Clinical laboratory results will be listed by patient and, as appropriate, summarized descriptively, which will include a display of change from baseline. Selected parameters will be presented in shift tables of baseline against worst grade test result. Laboratory values outside of the normal ranges will be identified. Laboratory values that meet Grade 3 or 4 criteria according to NCI CTCAE Version 5.0 will be listed and summarized.

ECG assessments will be evaluated for change of QTc from baseline as an exposure: response analysis. The Investigator's interpretation of QTc will be used in the clinical management of patients. The study analysis will use the Fridericia formula applied programmatically to the ECG data collected in CRFs using the QT interval and either the RR interval or the heart rate if the RR interval is not reported.

Vital signs, ECHO and ECG measurements will be listed for each patient at each visit. Descriptive statistics of observed values and changes from baseline will be summarized.

9.5.4 Patient Demographics, Baseline Characteristics and Disposition

Presentations of patient characteristics will include a summary of the following for all patients enrolling in the study:

- Demographics
- Baseline disease characteristics
- Pre-existing conditions/concurrent illness

- Prior therapies/surgeries

A summary of patient enrollment and disposition will include reasons for study discontinuation.

9.5.5 Analysis of Study Treatment Dosing

Study treatment administration will be described in terms of the total number of days administered, the median (range) of days administered for each agent separately and for the combination, dose intensity, and reasons for the deviations from planned therapy.

9.5.6 Time-to-Surgery

Time-to-surgery will be collected and described by Kaplan-Meier methods. Time-to-surgery is defined as the number of calendar days between D1 and the planned nephrectomy.

9.6 Other Study Endpoints

9.6.1 Pharmacokinetic Analysis

The PK sparse exposure data from this study may be used in the development of population PK and PK/PD models. Pharmacokinetic concentration levels and parameters will be determined, listed, and summarized for the PK evaluable population in the Pharmacokinetic Analysis Plan (PKAP). Only samples with acceptable PK (as defined in the PKAP) will be included in the summary statistics and a listing of individual data points or patients excluded from the analysis will be presented. Plasma concentrations will be listed by patient for the PK Population. Summary statistics of investigational agent concentrations will be reported by Visit Day.

9.6.2 Pharmacodynamic and Exploratory Analysis

No formal statistical analysis of pharmacodynamic endpoints will be performed. Pharmacodynamic data from each assay will be listed. Possible relationships between pharmacodynamic variables, PK parameters, safety, and clinical activity may be examined if appropriate.

9.7 Interim Analysis

No interim statistical analysis is planned during this study.

9.8 Data Monitoring Committee

No Data Monitoring Committee is planned during this study. However, the study team will monitor adverse events in the study on an ongoing basis to assess whether a lower starting dose of sitravatinib is warranted (see section 9.1.2).

10 ETHICS AND RESPONSIBILITIES

10.1 Ethical Conduct of the Study

This study will be conducted in accordance with International Ethical Guidelines for Biomedical Research Involving Human Patients (Council for International Organizations of Medical Sciences 2002), Guidelines for Good Clinical Practice (GCP) [International Conference on Harmonization (ICH) 1996], and concepts that have their origin in the Declaration of Helsinki (World Medical Association 1996, 2008 & 2013). Specifically, this study is based on adequately performed laboratory and animal experimentation; the study will be conducted under a protocol reviewed and approved by an IRB/EC/REB; the study will be conducted by scientifically and medically qualified persons; the benefits of the study are in proportion to the risks; the rights and welfare of the patients will be respected; the physicians conducting the study do not find the hazards to outweigh the potential benefits; and each patient will give his or her written informed consent before any protocol-driven tests or evaluations are performed.

10.2 Obligations of Investigators

The Investigator is responsible for complying with the protocol and all applicable regulations and guidelines governing clinical research. Additionally, he/she is responsible for ensuring that all participating staff members are adequately trained and competent to perform his/her assigned tasks.

All Investigators must provide the sponsor with a current *curriculum vitae*. Only Investigators and designated Sub-Investigators are permitted to sign CRFs and examination findings (e.g., laboratory results or ECGs).

The Investigator or designee is responsible for informing the patient of all available information relevant to his/her safety and obtaining signed, written consent from all participating patients. Additionally, the Investigator is responsible for monitoring patient safety and providing periodic and requested reports to the IRB/EC/REB.

The Investigator is responsible for the accuracy and completeness of all study records including CRFs, source documents, and the Site Trial Master File. The Investigator will allow the study monitor, Sponsor, auditor, regulatory agencies, and IRB/EC/REB full access to the study and source documents.

10.3 Institutional Review Board/Ethics Committee/Research Ethics Board

Prior to the shipment of clinical supplies or initiation of the study, the clinical trial protocol along with the informed consent form (ICF), Investigator's Brochure, and any other written information or instructions for the patient must be submitted to the IRB/EC/REB for written approval. The Investigator will provide the Sponsor with a copy of the IRB/EC/REB's written approval, as well as the membership list or a compliance statement from the IRB/EC/REB. The Investigator is responsible for notifying the IRB/EC of any Sponsor-approved amendments to the protocol or ICF,

SAEs occurring in patients treated at the study site in accordance with local IRB/EC/REB practice, and all expedited safety reports from SAEs occurring at other study sites participating in the drug development program.

10.4 Informed Consent Form

The ICF must contain all elements required by the Food and Drug Administration (FDA) under 21 Code of Federal Regulations (CFR) Part 50 and the ICH GCP guidelines (ICH E6) in addition to any other elements required by applicable national, state, provincial, and local regulations, or institutional policies.

All patients who choose to participate in the study must provide written consent after having had adequate time to consider whether they will participate in the study. The written consent must be obtained prior to any protocol-related procedures that are not part of the patient's normal medical care. The patient must be advised of his/her right to withdraw from the study at any time.

Written documentation of consent must be recorded in the patient's source documents, study records and CRF indicating the date the consent was signed. The patient should receive a signed copy of the consent form according to GCP guidelines.

10.5 Confidentiality

All information generated in this study is considered confidential, is subject to applicable privacy rules and regulations, and must not be disclosed to any person or entity not directly involved with the study unless prior written consent is gained from the Sponsor and otherwise except in accordance with applicable law or regulations. However, authorized regulatory officials, IRB/EC/REB personnel, the Sponsor and its authorized representatives (as and to the extent authorized in the patient's ICF) are allowed access to the records.

Identification of patients in CRFs shall be by study assigned patient numbers only. If required, the patient's full name may be made known to an authorized regulatory agency or other authorized official.

10.6 Reporting of Serious Breaches of the Protocol or ICH GCP

In the event of any prohibition or restriction (i.e., clinical hold) imposed by an applicable Regulatory Authority, or if the Investigator is aware of any new information which might influence the evaluation of the benefits and risks of the investigational product, the Sponsor must be informed immediately. In addition, the Investigator will inform the Sponsor immediately of any serious breaches of this protocol or of ICH GCP of which the Investigator becomes aware.

11 RECORDS MANAGEMENT

11.1 Source Documentation

Source documents include hospital or clinical patient charts, pertinent historical medical records, laboratory test reports, ECG tracings, pathology reports, radiographs, etc. All source documents must be legible. Data reported in CRFs and evidence of patient's informed consent must be documented in source documents.

11.2 Study Files and Records Retention

A CRF must be completed for each patient for whom informed consent for the study is obtained. The CRFs must be maintained by properly trained and delegated site representatives. The Principal Investigator has responsibility for ensuring the authenticity, accuracy, completeness and timeliness of all data collected in the CRF. CRFs must be signed by the Principal Investigator or by an authorized Sub-Investigator to attest that the information included is true.

The study site will maintain a Site Trial Master File in accordance with GCPs.

The Investigator shall retain all records for the longest of the following periods:
(i) 15 years; (ii) the period of time that conforms to ICH GCP guidelines; (iii) the period of time required by applicable law or regulations, or (iv) the period of time specified in the Clinical Research Agreement.

12 QUALITY CONTROL AND QUALITY ASSURANCE

12.1 Monitoring Procedures

Sponsor appointed Site Monitor(s) must be allowed access to all study records, original source documents, and investigational products throughout the duration of the study. These personnel are responsible to assess compliance with the protocol, appropriate health authority regulations, ICH GCP guidelines, and Sponsor requirements.

The study monitor is responsible for complying with the monitoring guidelines established by the Sponsor for the study, assessing the site's needs, and liaising with the assigned Sponsor staff.

If the Investigator withdraws from the study and relinquishes his/her responsibility for the maintenance and retention of records, he/she must notify the Sponsor in writing so arrangements can be made to properly store the study materials.

12.2 Auditing and Inspection Procedures

The Sponsor's Quality Assurance representatives, IRB/EC/REB reviewers, or inspectors from regulatory agencies may perform an audit or inspection at any time during or after completion of the clinical study. All study-related documentation must be made available to the designated auditor. In addition, representatives of applicable regulatory

health authorities may choose to inspect a study. A Sponsor representative will be available to assist in the preparation for such an inspection.

13 CHANGES IN STUDY CONDUCT

13.1 Protocol Amendments

Changes to the study protocol, except those intended to reduce immediate risk to study patients, may be made only by the Sponsor. A protocol change intended to eliminate an apparent immediate hazard to patients may be implemented immediately, provided the IRB/EC/REB is notified within 5 days. Any urgent safety measures taken by the Investigator to protect the study patients against any immediately life-threatening hazard must be reported immediately to the Sponsor.

Any permanent change to the protocol must be handled as a protocol amendment. The change and the justification will be documented in writing by the Sponsor, as an Administrative Letter or amended protocol. Protocol amendments will be provided with a separate document describing each change and rationale. The written Administrative Letter or amendment must be submitted to the IRB/EC/REB and the Investigator must await approval before implementing the changes. The Sponsor will be responsible for submitting protocol amendments to the appropriate regulatory authorities for approval.

If in the judgment of the IRB/EC/REB, the Investigator, and/or the Sponsor, the amendment to the protocol substantially changes the study design and/or increases the potential risk to the patient and/or has an impact on the patient's involvement as a study participant, the currently approved written informed consent form will require similar modification. In such cases, informed consents (revised as appropriate to address protocol amendments) will be obtained for patients enrolled in the study before continued participation.

13.2 Protocol Deviations

Prospective permission to deviate from the eligibility criteria for this protocol will not be provided by the Sponsor. Study specified assessments should not be omitted and the study treatment regimen should not deviate from protocol specifications. Minor, occasional adjustments in the clinic visit schedule may be necessary for logistical reasons (e.g., due to weather conditions) but must not become routine or systematically alter the study schedule. The IRB/EC/REB should be informed of any deviations that may affect a patient’s treatment or informed consent, especially those increasing potential risks, which must receive prior written approval by the IRB/EC/REB.

14 END OF TRIAL

14.1 End of Trial in a European Union Member State

End of Trial in a Member State of the European Union is defined as the time at which it is deemed that sufficient patients have been recruited and completed the study as stated in

the regulatory application [i.e., Clinical Trial Application (CTA)] and ethics application in the Member State.

14.2 End of Trial in all other Participating Countries

End of Trial in all other participating countries is defined as the time at which all patients enrolled in the study have completed the last study visit and data from those visits have been reviewed by the Investigator or designee.

14.3 Premature Termination

Premature termination of this study may occur at any time because of a regulatory authority decision, change in opinion of the IRB/EC/REB, drug safety concerns, or at the discretion of the Sponsor. In addition, the Sponsor retains the right to discontinue development of sitravatinib at any time. If termination becomes necessary, the Sponsor will inform the appropriate regulatory authorities of the termination and the reason. The Principal Investigator will inform the IRB/EC/REB of the same. In terminating the study, the Sponsor and the Principal Investigator will assure that adequate consideration is given to the protection of the patients' interests.

15 STUDY REPORT AND PUBLICATION POLICY

The Sponsor is responsible for preparing and providing the appropriate regulatory authorities with clinical study reports according to the applicable regulatory requirements.

The publication of study results will be governed by the applicable Clinical Research Agreement between the Sponsor and the Study Site and Investigator (as applicable).

16 REFERENCES

American Cancer Society (2017, Aug 1) Survival rates for kidney cancer by stage. Retrieved from https://www.cancer.org/cancer/kidney-cancer/detection-diagnosis-staging/survival-rates.html.

American Cancer Society, Cancer Facts & Figures 2018. American Cancer Society: Atlanta, Ga.

Apolo AB, Infante JR, Balmanoukian A, et al. Avelumab, an anti-programmed death-ligand 1 antibody, in patients with refractory metastatic urothelial carcinoma: Results from a multicenter, phase 1b study. J Clin Oncol. 2017;35:2117-2124.

Balar AV, Galsky MD, Rosenberg JE, et al. Atezolizumab as first-line therapy in cisplatin-ineligible patients with locally advanced and metastatic urothelial carcinoma: a single-arm, multicenter, phase 2 trial. Lancet. 2017;389:67-76. (a)

Balar AV, Castellano D, O'Donnell PH. First-line pembrolizumab in cisplatin-ineligible patients with locally advanced and unresectable or metastatic urothelial cancer (KEYNOTE-052): a multicenter, single-arm, phase 2 study. Lancet Oncol. 2017;18(11):1483-1492. (b)

Bellmunt J, de Wit R, Vaughn DJ, et al. Pembrolizumab as second-line therapy for advanced urothelial carcinoma. N Engl J Med. 2017 Mar 16;376(11):1015-1026.

Benkhoucha M, Santiago-Raber ML, Schneiter G, et al. Hepatocyte growth factor inhibits CNS autoimmunity by inducing tolerogenic dendritic cells and CD25+Foxp3+ regulatory T cells. Proc Natl Acad Sci USA. 2010;107(14):6424-6429.

Blume-Jensen P, Hunter T. Oncogenic kinase signalling. Nature. 2001;411:355-65.

Camenisch TD, Koller BH, Earp HS, Matsushima GK. A novel receptor tyrosine kinase, Mer, inhibits TNF-alpha production and lipopolysaccharide-induced endotoxic shock. J Immunol. 1999;162(6):3498-3503.

Chen PM, Liu KJ, Hsu PJ, et al. Induction of immunomodulatory monocytes by human mesenchymal stem cell-derived hepatocyte growth factor through ERK1/2. J Leukoc Biol. 2014;96(2):295-303.

Cho YH, Kim MS, Chung HS, et al. Novel immunotherapy in metastatic renal cell carcinoma. Investig Clin Urol. 2017 Jul;58(4):220-227.

Choueiri M, Tannir N, Jonasch E. Adjuvant and neoadjuvant therapy in renal cell carcinoma. Curr Clin Pharmacol. 2011 Aug;6(3):144-50.

Cohen RB, Oudard S. Antiangiogenic therapy for advanced renal cell carcinoma: management of treatment-related toxicities. Invest New Drugs. 2012 Oct;30(5):2066-79.

Eisenhauer EA, Therasse P, Bogaerts J, et al. New response evaluation criteria in solid tumours: revised RECIST guideline (version 1.1). Eur J Cancer. 2009;45(2):228-247.

Errarte P, Guarch R, Pulido R, et al. The expression of fibroblast activation protein in clear cell renal cell carcinomas is associated with synchronous lymph node metastases. PLoS One. 2016 Dec 29;11(12):e0169105.

Escudier B, Porta C, Schmidinger M, et al. Renal cell carcinoma: ESMO clinical practice guidelines for diagnosis, treatment and follow-up. Ann Oncol. 2014 Sep;25 Suppl 3:iii49-56.

Farsaci B, Higgins JP, Hodge JW. Consequence of dose scheduling of sunitinib on host immune response elements and vaccine combination therapy. Int J Cancer. 2012;130(8):1948-1959.

Finke JH, Rini B, Ireland J, et al. Sunitinib reverses type-1 immune suppression and decreases T-regulatory cells in renal cell carcinoma patients. Clin Cancer Res. 2008;14(20):6674-6682.

Fischer S, Gillessen S, Rothermundt C. Sequence of treatment in locally advanced and metastatic renal cell carcinoma. Transl Androl Urol. 2015 Jun;4(3):310-25.

Garfield K, LaGrange CA. Cancer, renal cell. StatPearls [Internet]. Treasure Island (FL): StatPearls Publishing; 2018 Jan 6.

Garton AJ, Seibel S, Lopresti-Morrow L, et al. Anti-KIT monoclonal antibody treatment enhances the antitumor activity of immune checkpoint inhibitors by reversing tumor-induced immunosuppression. Mol Cancer Ther. 2017 Apr;16(4):671-680.

Guislain A, Gadiot J, Kaiser A, et al. Sunitinib pretreatment improves tumor-infiltrating lymphocyte expansion by reduction in intratumoral content of myeloid-derived suppressor cells in human renal cell carcinoma. Cancer Immunol Immunother. 2015 Oct;64(10):1241-50.

Gustafson MP, Lin Y, Bleeker JS, et al. Intratumoral CD14+ cells and circulating CD14+HLA-DRlo/neg monocytes correlate with decreased survival in patients with clear cell renal cell carcinoma. Clin Cancer Res. 2015 Sep 15;21(18):4224-33.

Harshman LC, Puligandla M, Haas NB, et al. A phase III randomized study comparing perioperative nivolumab vs. observation in patients with localized renal cell carcinoma undergoing nephrectomy (PROSPER RCC). J Clin Oncol. 2017;35(S15): TPS4596.

Incorvaia L, Bronte G, Bazan V, et al. Beyond evidence-based data: scientific rationale and tumor behavior to drive sequential and personalized therapeutic strategies for the treatment of metastatic renal cell carcinoma. Oncotarget. 2016 Apr 19;7(16):21259-71.

Ji Y, Liu P, Li Y, et al. A modified toxicity probability interval method for dose-finding trials. Clin Trials. 2010 Dec;7(6):653-63.

Kao J, Ko EC, Eisenstein S, et al. Targeting immune suppressing myeloid-derived suppressor cells in oncology. Crit Rev Oncol Hematol. 2011 Jan;77(1):12-9.

Karam JA, Devine CE, Urbauer DL, et al. Phase 2 trial of neoadjuvant axitinib in patients with locally advanced nonmetastatic clear cell renal cell carcinoma. Eur Urol. 2014 Nov;66(5):874-80.

Ko JS, Zea AH, Rini BI, et al. Sunitinib mediates reversal of myeloid-derived suppressor cell accumulation in renal cell carcinoma patients. Clin Cancer Res. 2009;15(6):2148-2157.

Kujawski M, Zhang C, Herrmann A, et al. Targeting STAT3 in adoptively transferred T cells promotes their in vivo expansion and antitumor effects. Cancer Res. 2010;70(23):9599-9610.

Lee JJ, Liu DD. A predictive probability design for phase II cancer clinical trials. Clin Trials. 2008;5:93–106.

Lemke G, Rothlin CV. Immunobiology of the TAM receptors. Nat Rev Immunol. 2008;8(5):327-336.

Liu J, Blake SJ, Yong MC, et al. Improved efficacy of neoadjuvant compared to adjuvant immunotherapy to eradicate metastatic disease. Cancer Discov. 2016 Dec;6(12):1382-1399.

Liu XD, Hoang A, Zhou L, et al. Resistance to antiangiogenic therapy is associated with an immunosuppressive tumor microenvironment in metastatic renal cell carcinoma. Cancer Immunol Res. 2015 Sep;3(9):1017-29.

Massard C, Gordon MS, Sharma S, et al. Safety and efficacy of durvalumab (MEDI4736), an anti-programmed cell death ligand-1 immune checkpoint inhibitor, in patients with advanced urothelial bladder cancer. J Clin Oncol 2016; 34:3119-3125.

Mellman I, Coukos G, Dranoff G. Cancer immunotherapy comes of age. Nature. 2011;480(7378):480-489.

Motzer RJ, Escudier B, McDermott DF, et al. Nivolumab versus everolimus in advanced renal-cell carcinoma. N Engl J Med. 2015 Nov 5;373(19):1803-13.

Motzer RJ, Haas NB, Donskov F, et al. Randomized phase III trial of adjuvant pazopanib versus placebo after nephrectomy in patients with locally advanced renal cell carcinoma (RCC) (PROTECT). J Clin Oncol. 2017;35(15_suppl):4507.

Muglia VF, Prando A. Renal cell carcinoma: histological classification and correlation with imaging findings. Radiol Bras. 2015 May-Jun;48(3):166-74.

Najjar YG, Rayman P, Jia X, et al. Myeloid-derived suppressor cell subset accumulation in renal cell carcinoma parenchyma is associated with intratumoral expression of IL1β, IL8, CXCL5, and Mip-1α. Clin Cancer Res. 2017 May 1;23(9):2346-2355.

National Cancer Institute (U.S.). Common Terminology Criteria for Adverse Events: (CTCAE). U.S. Department of Health and Human Services, Bethesda, MD; 2017. https://ctep.cancer.gov/protocoldevelopment/electronic_applications/docs/CTCAE_v5_Quick_Reference_5x7.pdf

Okunishi K, Dohi M, Nakagome K, et al. A novel role of hepatocyte growth factor as an immune regulator through suppressing dendritic cell function. J Immunol. 2005;175(7):4745-4753.

OPDIVO® (nivolumab) injection, for intravenous use [package insert]. Bristol-Myers Squibb Company, Princeton, NJ; 2018. https://packageinserts.bms.com/pi/pi_opdivo.pdf

Ozao-Choy J, Ma G, Kao J, et al. The novel role of tyrosine kinase inhibitor in the reversal of immune suppression and modulation of tumor microenvironment for immune-based cancer therapies. Cancer Res. 2009;69(6):2514-2522.

Pan PY, Wang GX, Yin B, et al. Reversion of immune tolerance in advanced malignancy: modulation of myeloid-derived suppressor cell development by blockade of stem-cell factor function. Blood. 2008 Jan 1;111(1):219-28.

Paolino M, Choidas A, Wallner S, et al. The E3 ligase Cbl-b and TAM receptors regulate cancer metastasis via natural killer cells. Nature. 2014;507(7493):508-512.

Patel MR, Ellerton J, Infante JR, et al. Avelumab in metastatic urothelial carcinoma after platinum failure (JAVELIN Solid Tumor): pooled results from two expansion cohorts of an open-label phase 1 trial. Lancet Oncol. 2018;19(1)51-64.

Ravaud A, Motzer RJ, Pandha HS, et al. Adjuvant sunitinib in high-risk renal-cell carcinoma after nephrectomy. N Engl J Med. 2016 Dec 8;375(23):2246-2254.

Rini BI, Small EJ. Biology and clinical development of vascular endothelial growth factor-targeted therapy in renal cell carcinoma. J Clin Oncol. 2005 Feb 10;23(5):1028-43.

Rini BI, McDermott DF, Hammers H, et al. Society for Immunotherapy of Cancer consensus statement on immunotherapy for the treatment of renal cell carcinoma. J Immunother Cancer. 2016 Nov 15;4:81.

Rini BI, Campbell SC, Escudier B. Renal cell carcinoma. Lancet. 2009 Mar 28;373(9669):1119-32.

Rosenberg JE, Hoffman-Censits J, Powles R, et al. Atezolizumab in patients with locally advanced and metastatic urothelial carcinoma who have progressed following treatment with platinum-based chemotherapy: a single-arm, multicentre, phase 2 trial. Lancet. 2016;387:1909-1920.

Sciarra A, Cattarino S, Salciccia S, et al. The emerging role of targeted therapy in renal cell carcinoma (RCC): is it time for a neoadjuvant or an adjuvant approach? Crit Rev Oncol Hematol. 2012 Feb;81(2):151-62.

Sharma P, Callahan MK, Bono P, et al. Nivolumab monotherapy in recurrent metastatic urothelial carcinoma (CheckMate 032): a multicentre, open-label, two-stage, multi-arm, phase 1/2 trial. Lancet. 2016;17:1590-1598.

Sharma P, Retz M, Siefker-Radtke A, et al. Nivolumab in metastatic urothelial carcinoma after platinum therapy (CheckMate 275): a multicentre, single-arm, phase 2 trial. Lancet Oncol. 2017;18:312-322.

Siegel RL, Miller KD, Jemal A. Cancer statistics, 2018. CA Cancer J Clin. 2018 Jan;68(1):7-30.

Silberstein JL, Millard F, Mehrazin R, et al. Feasibility and efficacy of neoadjuvant sunitinib before nephron-sparing surgery. BJU Int. 2010 Nov;106(9):1270-6.

Singhal E, Sen P. Hepatocyte growth factor-induced c-Src-phosphatidylinositol 3-kinase-AKT-mammalian target of rapamycin pathway inhibits dendritic cell activation by blocking IκB kinase activity. Int J Biochem Cell Biol. 2011;43(8):1134-1146.

Tannir NM, Schwab G, Grünwald V. Cabozantinib: an active novel multikinase inhibitor in renal cell carcinoma. Curr Oncol Rep. 2017 Feb;19(2):14.

Terme M, Pernot S, Marcheteau E, et al. VEGFA-VEGFR pathway blockade inhibits tumor-induced regulatory T-cell proliferation in colorectal cancer. Cancer Res. 2013;73(2):539-549.

Topalian SL, Drake CG, Pardoll DM. Immune checkpoint blockade: a common denominator approach to cancer therapy. Cancer Cell. 2015;27(4):450-461.

Vanneman M, Dranoff G. Combining immunotherapy and targeted therapies in cancer treatment. Nat Rev Cancer. 2012;12(4):237-251.

Yen BL, Yen ML, Hsu PJ, et al. Multipotent human mesenchymal stromal cells mediate expansion of myeloid-derived suppressor cells via hepatocyte growth factor/c-D201met and STAT3. Stem Cell Reports. 2013;1(2):139-151.

APPENDIX 1. ECOG PERFORMANCE STATUS

ECOG PERFORMANCE STATUS*	
**Grade**	**ECOG**
0	Fully active, able to carry on all pre-disease performance without restriction
1	Restricted in physically strenuous activity but ambulatory and able to carry out work of a light or sedentary nature, e.g., light house work, office work
2	Ambulatory and capable of all self-care but unable to carry out any work activities. Up and about more than 50% of waking hours
3	Capable of only limited self-care, confined to bed or chair more than 50% of waking hours
4	Completely disabled. Cannot carry on any self-care. Totally confined to bed or chair
5	Dead

APPENDIX 2. MEDICATIONS TO BE AVOIDED OR USED WITH CAUTION DURING STUDY TREATMENT

Bold font indicates medications that might be relatively commonly used.

Italic font indicates medications for indications that are exclusionary for the current study or would likely result in discontinuation from study treatment with sitravatinib for management of a concurrent illness.

MEDICATIONS THAT SHOULD BE AVOIDED

Drugs with a Known Risk of Torsades de Pointes

Amiodarone, anagrelide, *arsenic trioxide*, astemizole (off US market), **azithromycin**, bepridil (off US market), chloroquine, **chlorpromazine**, cilostazol, **ciprofloxacin**, cisapride (off US market), **citalopram**, **clarithromycin**, cocaine, disopyramide, dofetilide, domperidone (not on US market), donepezil, dronedarone, **droperidol**, **erythromycin**, **escitalopram**, flecainide, fluconazole, gatifloxacin (off US market), grepafloxacin (not on US market), halofantrine (not on US market), haloperidol, ibogaine (not on US market), ibutilide, levofloxacin, levomepromazine / methotrimeprazine (not on US market), levomethadyl (off US market), levosulpiride (not on US market), mesoridazine (off US market), **methadone**, moxifloxacin, **ondansetron**, *oxaliplatin*, *pentamidine*, pimozide, probucol (off US market), procainamide, propofol, quinidine, roxithromycin (not on US market), sevoflurane, sotalol, sparfloxacin (off US market), sulpiride (not on US market), sultopride (non on US market), terfenadine (off US market), terlipressin (not on US market), terodiline (not on US market), thioridazine, *vandetanib*.

CAUTION WHEN TAKING THE FOLLOWING MEDICATIONS

Sensitive Substrates and Substrates with Narrow Therapeutic Index for P-gp and BCRP transporters

Enzyme	
P-gp	Aliskiren, ambrisentan, colchicine, dabigatran etexilate, **digoxin**, *everolimus*, **fexofenadine**, *imatinib*, *lapatinib*, *maraviroc*, *nilotinib*, posaconazole, ranolazine, saxagliptin, sirolimus, **sitagliptin**, talinolol, tolvaptan, *topotecan.*
BCRP	Methotrexate, *mitoxantrone*, *imatinib*, *irinotecan*, *lapatinib*, **rosuvastatin**, sulfasalazine, *topotecan.*

Sensitive Substrates and Substrates with Narrow Therapeutic Index for the indicated CYP Enzymes

Enzyme	
CYP2B6	Bupropion.
CYP2C8	Repaglinide.
CYP2D6	Atomoxetine, desipramine, **dextromethorphan**, *eliglustat,* nebivolol, **nortriptyline**, perphenazine, tolterodine, venlafaxine.
CYP3A	Alfentanil, avanafil, budesonide, buspirone, conivaptan, darifenacin, *darunavir*, *dasatinib*, dronedarone, ebastine, eletriptan, eplerenone, *everolimus*, **felodipine**, ibrutinib, *indinavir*, lomitapide, **lovastatin**, lurasidone, *maraviroc*, **midazolam**, naloxegol, nisoldipine, quetiapine, *saquinavir*, sildenafil, **simvastatin**, sirolimus, tacrolimus, ticagrelor, *tipranavir*, tolvaptan, triazolam, vardenafil.

Drugs with Conditional Risk of Torsades de Pointes

**Amantadine**, amisulpride (not on US market), amitriptyline, amphotericin B, *atazanavir*, bendroflumethiazide / bendrofluazide (not on US market), chloral hydrate, **diphenhydramine**, doxepin, **esomeprazole**, **famotidine**, **fluoxetine**, fluvoxamine**,** **furosemide / frusemide**, galantamine, garenoxacin (not on US market), **hydrochlorothiazide**, hydroxychloroquine, hydroxyzine, indapamide, itraconazole, ivabradine, ketoconazole, **lansoprazole**, **loperamide**, **metoclopramide**, metolazone, metronidazole, *nelfinavir*, olanzapine, **omeprazole**, **pantoprazole**, **paroxetine**, piperacillin/tazobactam, posaconazole, propafenone, quetiapine, quinine sulfate, ranolazine, **sertraline**, solifenacin, *telaprevir*, torsemide / torasemide, trazodone, voriconazole, ziprasidone.

APPENDIX 3. ABBREVIATED PRESENTATION OF RECIST VERSION 1.1 GUIDELINES

RECIST (Response Evaluation Criteria in Solid Tumors) 1.1 guidelines will be used in the assessment of the point in time response for the on-study assessment. Confirmation of response will not be used since there is only one on-study assessment.

Categorizing Lesions at Baseline

Measurable Lesions

- Accurately measured in at least one dimension.
- When assessed by CT or MRI, longest diameter at least 10 mm or greater (slice thickness 5-8 mm), measured in the axial plane. If the slice thickness is greater than 5 mm (including any inter-slice gap), the longest diameter must be at least twice the slice thickness.
- Malignant lymph nodes with a short axis (defined as the largest measurement perpendicular to the longest diameter of the lesion) 15 mm or greater when assessed by CT or MRI.

The shortest axis is used as the diameter for malignant lymph nodes, longest axis for all other lesions.

Non-Measurable Disease

- Lesions too small to be considered measurable (including nodes with short axis between 10 and 14.9 mm) or truly non-measurable disease such as pleural or pericardial effusions, ascites, inflammatory breast disease, leptomeningeal disease, lymphangitic involvement of skin or lung, and abdominal masses identified by physical exam that are not measurable by reproducible imaging techniques.
- Bone disease is non-measurable with the exception of soft tissue components that can be evaluated by CT or MRI and meet the definition of measurability at baseline.
- Previously irradiated lesions (or those subjected to other local treatment) are non-measurable unless it they have progressed since completion of treatment.

Normal Lesions

- Non-malignant simple cysts should not be recorded either as target or non-target disease. Cystic lesions thought to represent cystic metastases can be measurable lesions, if they meet the specific definition above.

- Lymph nodes with short axis < 10 mm are considered normal and should not be followed as disease.

Tumor Assessments

All sites of disease must be assessed at baseline. Baseline assessments should be done as close as possible prior to study start. All required scans must be done within the window of time specified in the Schedule of Assessments prior to treatment. If the baseline assessment is inadequate, subsequent statuses generally should be indeterminate.

The determination of whether lesions are measurable is performed only at baseline. "Measurable" at baseline means eligible for selection as target lesions, and thus for quantitative assessment throughout the trial. Once selected as a target lesion, a lesion remains target throughout the trial.

Target Lesions

All measurable lesions up to a maximum of 2 lesions per organ, 5 lesions in total, representative of all involved organs, should be identified as target lesions at baseline. Target lesions should be selected on the basis of size (longest lesions) and suitability for accurate repeated measurements. Record the longest diameter for each lesion, except in the case of pathological lymph nodes for which the short axis should be recorded. The sum of the diameters (longest for non-nodal lesions, short axis for nodal lesions) for all target lesions at baseline will be the basis for comparison to look for partial response at later assessments.

- If 2 target lesions coalesce the longest diameter measurement of the coalesced mass is used. If a large target lesion splits, the sum of the parts is used.

- Measurements for target lesions that become small should continue to be recorded. If a target lesion becomes too small to measure, 0 mm should be recorded if the lesion is considered to have disappeared; otherwise a default value of 5 mm should be recorded.

- When nodal lesions decrease to < 10 mm (normal), the actual measurement should still be recorded.

Non-Target Lesions

All non-measurable disease is non-target. All measurable lesions not identified as target lesions are also included as non-target disease. Measurements are not required but rather qualitative evaluations of status will be recorded. Multiple non-target lesions in one organ may be recorded as a single item on the CRF (e.g., 'multiple liver metastases').

Objective Response Status at Each Evaluation

Disease sites must be assessed using the same technique as baseline, including consistent administration of contrast. If not, subsequent objective statuses may be indeterminate.

Target Disease

- Complete Response (CR): Complete disappearance of all target lesions with the exception of nodal disease. All target nodes must decrease to normal size (short axis < 10 mm). All target lesions must be assessed.

- Partial Response (PR): Greater than or equal to 30% decrease under baseline of the sum of diameters of all target measurable lesions. The short diameter is used in the sum for target nodes, while the longest diameter is used in the sum for all other target lesions. All target lesions must be assessed.

- Stable Disease (SD): Does not qualify for CR, PR or Progression. All target lesions must be assessed. Stable can follow PR only in the rare case that the sum increases by less than 20% from the nadir, but enough that a previously documented 30% decrease no longer holds.

- Progressive Disease (PD): 20% increase in the sum of diameters of target measurable lesions above the smallest sum observed (over baseline if no decrease in the sum is observed during therapy) with a minimum absolute increase of 5 mm.

- Indeterminate: Progression has not been documented, and

  - one or more target lesions have not been assessed,

  - or assessment methods used were inconsistent with those used at baseline and impaired assessment,

  - or one or more target lesions cannot be measured accurately (e.g., poorly visible unless due to being too small to measure),

  - or one or more target lesions were excised or irradiated and have not reappeared or increased.

Non-Target Disease

- CR: Disappearance of all non-target lesions and normalization of tumor marker levels. All lymph nodes must be 'normal' in size (< 10 mm short axis).

- Non-CR/Non-PD: Persistence of any non-target lesions and/or tumor marker level above the normal limits.

- PD: Unequivocal progression of pre-existing lesions. Generally, the overall tumor burden must increase sufficiently to merit discontinuation of therapy. In the presence SD or PR in target disease, progression due to unequivocal increase in non-target disease should be rare.

- Indeterminate: Progression has not been determined and one or more non-target sites were not assessed or assessment methods were inconsistent with those used at baseline.

New Lesions

The appearance of any new unequivocal malignant lesion indicates PD. If a new lesion is equivocal, for example due to its small size, continued assessment will clarify the etiology. If repeat assessments confirm the lesion, then progression should be recorded on the date of the initial assessment. A lesion identified in an area not previously scanned will be considered a new lesion.

Supplemental Investigations

If CR determination depends on a residual lesion that decreased in size but did not disappear completely, it is recommended the residual lesion be investigated with biopsy or fine needle aspirate. If no disease is identified, objective status is CR.

Point in Time Objective Response Guide

Target Lesions	Non-Target Lesions	New Lesion	Point in Time Response
CR	CR	No	CR
CR	Non-CR/Non-PD	No	PR
PR	Non-PD	No	PR
SD	Non-PD	No	SD
PD	Any	Yes or No	PD
Any	PD	Yes or No	PD
Any	Any	Yes	PD

Subjective Progression

Patients requiring discontinuation of treatment due to worsening health status attributable to advancement of the malignancy under study but without objective evidence of disease progression should not be reported as PD on tumor assessment CRFs. This should be indicated on the End of Study CRF as off treatment due to Global Deterioration of Health Status.

APPENDIX 4. DOSE DE-ESCALATION DECISION TABLE USING THE mTPI METHOD

The table below will be used for decision making with regards to potential sitravatinib starting dose de-escalation in subsequent patients. The assessment is based on the number of patients with toxicity at a given dose as defined in Section 9.1 and assumes a maximum toxicity level of no more than 20% at the tolerated dose. The letters in different colors are computed based on the decision rules under the mTPI method (Ji-2010) and represent different dose-finding actions which include: S = Stay at the current dose; D = De-escalate to the next lower dose; and U = The current dose in unacceptably toxic.

Number of toxicities	Number of patients treated at current dose																								
	1	2	3	4	5	6	7	8	9	10	11	12	13	14	15	16	17	18	19	20	21	22	23	24	25
0	S	S	S	S	S	S	S	S	S	S	S	S	S	S	S	S	S	S	S	S	S	S	S	S	S
1	DU	D	S	S	S	S	S	S	S	S	S	S	S	S	S	S	S	S	S	S	S	S	S	S	S
2		DU	DU	D	S	S	S	S	S	S	S	S	S	S	S	S	S	S	S	S	S	S	S	S	S
3			DU	DU	DU	DU	D	S	S	S	S	S	S	S	S	S	S	S	S	S	S	S	S	S	S
4				DU	DU	DU	DU	DU	DU	D	S	S	S	S	S	S	S	S	S	S	S	S	S	S	S
5					DU	DU	DU	DU	DU	DU	DU	DU	DU	S	S	S	S	S	S	S	S	S	S	S	S
6						DU	DU	DU	DU	DU	DU	DU	DU	DU	DU	DU	S	S	S	S	S	S	S	S	S
7							DU	DU	DU	DU	DU	DU	DU	DU	DU	DU	DU	DU	DU	DU	S	S	S	S	S
8								DU	DU	DU	DU	DU	DU	DU	DU	DU	DU	DU	DU	DU	DU	DU	DU	DU	S
9									DU	DU	DU	DU	DU	DU	DU	DU	DU	DU	DU	DU	DU	DU	DU	DU	DU
10										DU	DU	DU	DU	DU	DU	DU	DU	DU	DU	DU	DU	DU	DU	DU	DU
11											DU	DU	DU	DU	DU	DU	DU	DU	DU	DU	DU	DU	DU	DU	DU
12												DU	DU	DU	DU	DU	DU	DU	DU	DU	DU	DU	DU	DU	DU
13													DU	DU	DU	DU	DU	DU	DU	DU	DU	DU	DU	DU	DU
14														DU	DU	DU	DU	DU	DU	DU	DU	DU	DU	DU	DU
15															DU	DU	DU	DU	DU	DU	DU	DU	DU	DU	DU
16																DU	DU	DU	DU	DU	DU	DU	DU	DU	DU
17																	DU	DU	DU	DU	DU	DU	DU	DU	DU
18																		DU	DU	DU	DU	DU	DU	DU	DU
19																			DU	DU	DU	DU	DU	DU	DU
20																				DU	DU	DU	DU	DU	DU
21																					DU	DU	DU	DU	DU
22																						DU	DU	DU	DU
23																							DU	DU	DU
24																								DU	DU
25																									DU

S = Stay at the current dose
D = De-escalate to the next lower dose
U = The current dose is unacceptably toxic
Tolorated Dose = ≤ 20% toxicities
Sample Size = 25
Epsilon1 = 0.05
Epsilon2 = 0.05
