# Supplementary material for: Phase II trial of neoadjuvant sitravatinib plus nivolumab in patients undergoing nephrectomy for locally advanced clear cell renal cell carcinoma
Source: Nat Commun. 2023 May 10;14:2684. doi: 10.1038/s41467-023-38342-7 (PMC10172300; doi:10.1038/s41467-023-38342-7)
Supplement: Supplementary file 2 — Reporting Summary [file 41467_2023_38342_MOESM2_ESM.pdf]

nature portfolio

Corresponding author(s): Jose A Karam, Pavlos Msaouel

Last updated by author(s): Jun 24, 2022

Reporting Summary

Nature Portfolio wishes to improve the reproducibility of the work that we publish. This form provides structure for consistency and transparency in reporting. For further information on Nature Portfolio policies, see our Editorial Policies and the Editorial Policy Checklist.

Statistics

For all statistical analyses, confirm that the following items are present in the figure legend, table legend, main text, or Methods section.

n/a	Confirmed	
☐	☒	The exact sample size (*n*) for each experimental group/condition, given as a discrete number and unit of measurement
☐	☒	A statement on whether measurements were taken from distinct samples or whether the same sample was measured repeatedly
☐	☒	The statistical test(s) used AND whether they are one- or two-sided<br>*Only common tests should be described solely by name; describe more complex techniques in the Methods section.*
☒	☐	A description of all covariates tested
☒	☐	A description of any assumptions or corrections, such as tests of normality and adjustment for multiple comparisons
☐	☒	A full description of the statistical parameters including central tendency (e.g. means) or other basic estimates (e.g. regression coefficient) AND variation (e.g. standard deviation) or associated estimates of uncertainty (e.g. confidence intervals)
☐	☒	For null hypothesis testing, the test statistic (e.g. *F*, *t*, *r*) with confidence intervals, effect sizes, degrees of freedom and *P* value noted<br>*Give P values as exact values whenever suitable.*
☒	☐	For Bayesian analysis, information on the choice of priors and Markov chain Monte Carlo settings
☒	☐	For hierarchical and complex designs, identification of the appropriate level for tests and full reporting of outcomes
☒	☐	Estimates of effect sizes (e.g. Cohen's *d*, Pearson's *r*), indicating how they were calculated

Our web collection on statistics for biologists contains articles on many of the points above.

Software and code

Policy information about availability of computer code

Data collection

Medidata RAVE version 2017.2.2

Immunohistochemistry (IHC) of PD-L1 data: Available FFPE tumor tissue samples were collected from patients pre- and post-neoadjuvant therapies for single chromogenic IHC staining of tumor PD-L1 using a a Leica Bond Max autostainer system (Leica Biosystems). Automated standard Leica protocol was utilized: as detailed in the methods of the manuscript. The following reagents were used: antigen retrieval was performed with Bond Solution no. 2 (Leica Biosystems, equivalent to ethylenediaminetetraacetic acid, pH 9.0), primary antibody (clone 28-8, catalog no. ab205921, dilution 1:100; Abcam), Bond Polymer Refine Detection kit (Leica Biosystems) with 3,3′-diaminobenzidine as the chromogen. Data were collected using Microsoft Excel v.2016.

Multiparameter flow cytometry data: Fresh uninvolved normal kidney and tumor tissues collected at surgery were disaggregated using the BD Medimachine System (BD Biosciences) to make a single cell suspension for flow cytometry staining. Detailed methods and antibodies are described in the manuscript and in the antibody section below. The following reagents were used: 5% goat serum (catalog no. G9023, Sigma), 1× DPBS with 1% bovine serum albumin (catalog no. A8577, Sigma), eBioscience Foxp3/Transcription Factor Staining Buffer Set (catalog no. 00-5523-00, Thermo Fisher Scientific), BD Fix/Perm buffer solution from the Fixation/Permeabilization kit (cat. 554714, BD Biosciences), BD Perm buffer I solution. Data were acquired with the Fortessa X20 (BD Bioscience) using BD FACSDiva software v8.0.1. and Microsoft Excel (v. 2016).

Multiplex immunofluorescence (mIF) staining data: Using an automated staining system (BOND-RX; Leica Microsystems), 4-µm-thick FFPE tumor sections were stained for two panels containing antibodies against the antibodies in panel 1 and panel 2 detailed in the manuscript methods and in the antibody section below. All the markers were stained in sequence using their respective fluorophore contained in the Opal 7 kit (catalog no. NEL797001KT, Akoya Biosciences/PerkinElmer). The stained slides were scanned using the multispectral microscope,

Vectra v.3.0.3 imaging system (Akoya Biosciences/ PerkinElmer), under fluorescence conditions in low magnification at ×10. After the slides were scanned in low magnification, a pathologist selected around five regions of interest (ROIs; each ROI: 0.3345mm2) per sample to cover around 1.65 mm2 of tumor tissue using the phenochart v.1.0.9 viewer (Akoya Biosciences/PerkinElmer). The data were collected using Microsoft Excel v.2016.

Data analysis

SAS® Version 9.4; Phenochart Software image viewer Version 1.0.12; InForm 2.4.8 image analysis software; R studio 3.5.3 (Phenopter 0.2.2 packet, Akoya Biosciences); HTG parser and HTG EdgeSeq Reveal software; NeoLYTX v2.0; FlowJo Software Version 10.7.1
Monoceros software packages: R (Version 3.6.1); Limma (Version 3.40.9); MSigDB (version 7.0); GSEA (version 2.2.4).

Immunohistochemistry analysis: PD-L1 stained slides were scored by standard microscopy following the recommendations of the International Association for the Study of Lung Cancer guidelines (PMID: 29800747). Two pathologists evaluated PD-L1 expression in the membrane of viable malignant cells and the results were reported as percentage of malignant cells with any positive membrane staining (Tumor Proportion Score, TPS). The results were plotted using GraphPad Prism v.9.00.

Flow Cytometry analysis: Data were analyzed using FlowJo Software v.10.7.1 (Tree Star, Inc.). Dead cells were stained using LIVE/DEAD Fixable Yellow Dead Cell Stain dye (catalog no. L-34968, Life Technologies) and excluded from the analysis. Experiments and gating related to the presented results were conducted once. Analyzed data were plotted using GraphPad prism v. 9.00.

Multiplex immunofluorescence analysis: ROIs were selected and analyzed by a pathologist using InForm v.2.8.2 image analysis software (Akoya Biosciences). In panel 1, the colocalization of protein surface markers was used to identify malignant cells expressing (AE1/AE3+), malignant cells expressing PD-L1 (AE1/ AE3+PD-L1+), T cell population expressing (CD3+), cytotoxic T cells (CD3+CD8+), antigen-experienced T cells (CD3+PD-1+), cytotoxic antigen-experienced T cells (CD3+CD8+PD-1+), T cells PD-L1+ (CD3+PD-L1+), cytotoxic T cells PD-L1+ (CD3+CD8+PD-L1+), cytotoxic T cells antigen-experienced expressing PD-L1+ (CD3+CD8+PD-1+PD-L1+), macrophages (CD68+) and macrophages expressing PD-L1 (CD68+PD-L1+). In panel 2 the positive expression of CD3 protein surface was used to identify T cells (CD3+), and the colocalization of more than one protein surface marker was used to identify cytotoxic T cells (CD3+CD8+), cytotoxic activated T cells (CD3+CD8+Granzyme B+), memory T cells (CD3+CD45RO+), effector/memory cytotoxic T cells (CD3+CD8+CD45RO+), regulatory T cells [(CD3+FoxP3+)-(CD3+CD8+FOXP3+), and memory/regulatory T cells (CD3+CD45RO+FoxP3+). Cell densities of each cell population were quantified, and the final data were expressed as the number of cells populations per mm2 in two compartments: tumor nests and tumor stroma. Malignant cells and macrophages expressing PD-L1 were also expressed as percentages. All the data were consolidated using the R studio v.3.5.3 (Phenopter v.0.2.2 packet, Akoya Biosciences/PerkinElmer) and SAS v.7.1 Enterprise. Experiments and scorings related to the presented micrographs were conducted once. The data were plotted using GraphPad Prism v.9.00.

The methods section of the manuscript described all the tools that have been used to perform the analysis. All these tools are open source and are currently made available by their authors from their corresponding repositories.
The custom pipeline developed by Monoceros Biosystems is a wrapper for the aforementioned tools, containing proprietary code to automate the execution of the tools in a streamlined fashion and automatically generate formatted output files. These pipelines have been designed to integrate and make use of Monoceros Biosystems infrastructure and may not be generalizable, hence the code has not been made publicly available.

For manuscripts utilizing custom algorithms or software that are central to the research but not yet described in published literature, software must be made available to editors and reviewers. We strongly encourage code deposition in a community repository (e.g. GitHub). See the Nature Portfolio guidelines for submitting code & software for further information.

Data

Policy information about availability of data

All manuscripts must include a data availability statement. This statement should provide the following information, where applicable:

- Accession codes, unique identifiers, or web links for publicly available datasets
- A description of any restrictions on data availability
- For clinical datasets or third party data, please ensure that the statement adheres to our policy

The trial protocol is available at https://clinicaltrials.gov/ProvidedDocs/21/NCT03680521/Prot_000.pdf and as a Supplementary File with this submission.
Mirati will honour legitimate requests for clinical trial data from qualified researchers, upon request, as necessary for conducting methodologically sound research. Mirati will provide access to data and clinical study reports (CSRs) for clinical trials for which results are posted on the clinicaltrials.gov registry for products or indications that have been approved by regulators in the US and EU. In general, data will be made available for request approximately 12 months after clinical trial completion. Relevant components of the protocol and statistical analysis plan for the 516-002 study will also be made available upon request. No additional databases or datasets were used in study. For the HTG EdgeSeq analysis, data are available at https://www.ncbi.nlm.nih.gov/geo/query/acc.cgi?acc=GSE212525 (GEO accession number GSE212525).

Human research participants

Policy information about studies involving human research participants and Sex and Gender in Research.

Reporting on sex and gender

Finding apply to both sexes. Sex was not considered in study design; both males and females were eligible for study. Sex was determined based on self-reporting.
For the safety population (N=20)
Sex, n (%)
Male, 16 (80)
Female, 4 (20)

See manuscript for details.

Population characteristics	Eligible patients for Study 516-002 (NCT3680521) were aged ≥18 years with previously untreated locally advanced ccRCC without evidence of metastatic disease. All patients underwent an initial diagnostic biopsy of their renal lesion to confirm clear cell histology. Eligible patients had clinical stage cT2-T3b, N0, M0 tumours, with retroperitoneal lymph nodes ≤1 cm in size (considered clinical N0) and were candidates for partial or radical nephrectomy. Additional key inclusion criteria were measurable disease according to Response Evaluation Criteria in Solid Tumors version 1.1 and an Eastern Cooperative Oncology Group performance status score of 0 or 1. Key exclusion criteria included inability to undergo a baseline tumour biopsy; a clinical status indicating the need for immediate (within 6 weeks) surgery, regardless of whether neoadjuvant therapy was to be administered; autoimmune disease; or any current/prior use of an immunosuppressant (>10 mg daily prednisone equivalent).
Recruitment	Patients were recruited on Study 516-002 (NCT3680521) by urologists and medical oncologists who treat ccRCC at MD Anderson. The total number of patients enrolled was 25. All patients enrolled in the study underwent baseline biopsy. Of these, five discontinued the study (three had ineligible histology data, one was excluded for using concomitant treatment, which was not permitted by the study protocol, and one patient was found to be non-compliant with the study procedures and requirements).<br><br>All patients provided written informed consent to participate based on the principles of the Declaration of Helsinki.<br><br>All patients were recruited at MD Anderson by urologists and medical oncologists. Since the study was conducted at a single site, there is a bias to recruiting patients who either live near MD Anderson or who are willing to travel long distances to receive care at the nationally-recognized cancer center. In addition, ccRCC occurs at incidence rates of approximately two-fold greater in men compared with women, is found at significantly higher rates in Caucasian than other ethnicities, and increases with advancing age, peaking in the sixth to eighth decade of life. This epidemiological factors likely skewed the demographic profile of the patients, and any additional differences in demographic characteristics from what has been observed at a general population level could be attributed to sampling randomness (Table 1).
Ethics oversight	Study 516-002 (NCT3680521) was approved by the Institutional Review Board (IRB) of MD Anderson (MDACC protocol 2018-0296). Patient samples for correlative studies were approved by the same IRB and MDACC protocol.

Note that full information on the approval of the study protocol must also be provided in the manuscript.

Field-specific reporting

Please select the one below that is the best fit for your research. If you are not sure, read the appropriate sections before making your selection.

☒ Life sciences ☐ Behavioural & social sciences ☐ Ecological, evolutionary & environmental sciences

For a reference copy of the document with all sections, see nature.com/documents/nr-reporting-summary-flat.pdf

Life sciences study design

All studies must disclose on these points even when the disclosure is negative.

Sample size	At the time the study was initiated (2018), with then currently available treatments, the percentage of patients with a point in time objective response prior to surgery was assumed to be 5% (p0); thus, this rate was considered uninteresting. The target percentage of patients with a point in time objective response prior to surgery using sitravatinib and nivolumab in this study was assumed to be 30% (p1). Controlling for a Type 1 error (α) of 0.05, and using an exact test (two-sided), with 18 clinical activity evaluable patients, there was 80% power to rule out a percentage of patients with a point in time objective response prior to surgery of 5% assuming percentage of patients with a point in time objective response prior to surgery of 30%. Assuming a non-evaluable rate between 25%-30% enrolled patients to clinical activity evaluable patients, the study will enroll approximately 25 patients in order to get 18 clinical activity evaluable patients. The number of patients actually enrolled was 25, of which 17 were clinical activity evaluable.
Data exclusions	All 25 patients enrolled in the study underwent baseline biopsy. Of these, five discontinued the study (three had ineligible histology data, one was excluded for using concomitant treatment, which was not permitted by the study protocol, and one patient was found to be non-compliant with the study procedures and requirements). At mid-study, the 20 patients comprising the safety analysis population, all of whom had received sitravatinib treatment, were re-biopsied. All 17 of the patients in the efficacy-evaluable population underwent surgery, including one patient with bilateral disease who had two resections. One additional patient in the safety population who underwent surgery was found to have marked metastatic disease, making them ineligible for efficacy analysis. Of the other two patients who were excluded from the efficacy analyses, one with metastatic disease did not undergo resection, and one who did not receive treatment with nivolumab did eventually have surgery, but was not evaluable for clinical response due to the lack of a restaging scan prior to their operation. Correlative data was included based on tissue/blood sample availability and passing quality control metrics.<br><br>Correlative analyses: All samples available and considered appropriate based on QC for correlative studies at time of analyses were included.<br><br>Flow cytometry analysis: Available samples were excluded from analysis if they did not pass the respective QC for a given assay as detailed in Methods and Figure Legends of the manuscript.
Replication	Replication was not applicable to this study as this was a clinical study with unique patient samples. All techniques and reagents used for the

correlative analyses of this study had been previously optimized and validated.

As this was a clinical study, no efforts to verify reproducibility were attempted. See sample size section for rationale on target patient enrollment. For correlative studies, efforts were made to identify trends across patients in the study.

In general, please note that technical replicates were not performed due to limited sample volume. Biological replicates were not performed in order for blood draws and tumor biopsies to be kept to a minimum on study participants.
Figures 1-2: no reproducibility represented
Figure 3: The FDA guidance on Bioanalytical Validation (which includes sample analysis) requires verification of assay precision and accuracy by evaluation of Incurred Sample Reanalysis (ISR). The guidance details repeat of at least 10% of the first 1000 samples and 5% of remaining samples for this evaluation. Incurred sample reanalysis was performed for 22% of all patient samples in this study. This included reanalysis of samples from 13 subjects and included high and low concentrations for each subject (when possible), for a total of 22 samples reassayed for this determination. The % difference between the reported value and the ISR value was within ±20% for 22 out of 22 (100%) of samples evaluated. ISR performance for MGCD516 in study 516-002 exceeded the 67% pass rate required by the guidance to demonstrate acceptable assay reproducibility.
Figure 4: Replicates were not performed, as this is an analysis of the available molecular data from the study. A second study would be needed to attempt reproducibility, which is beyond the scope of this work.
Figure 5: PD-L1 figure: No replicates were performed. One tumor sample per patient per timepoint (i.e. baseline, mid-study and surgery) were evaluated and slides were analyzed for PD-L1 expression by a pathologist
Figures 6-7: Replicates were not performed.

Randomization

Allocation was not random as this was a single arm study. Covariates are used to adjust any imbalance of baseline prognostic factors that could potentially impact the treatment difference between experimental arm and control arm. Since the 516-002 study is a single arm study without any control arm it is not applicable to apply covariate adjustment in the analysis.

Blinding

Blinding is not relevant to our study as this was a single arm study. In addition, the primary endpoint was objective response rate (ORR), which objectively measures effect on tumor attributable to drug and is less susceptible to bias compared to other clinical endpoints; given the exploratory nature of this pilot Phase 2 study, the authors did not perform a centralized review of radiological tumor scans to ascertain lack of assessment bias.
All in vitro studies were planned and performed to ensure that each experiment contains all groups and appropriate controls. Investigators were not blinded to group allocation during data collection and/or analysis.

Reporting for specific materials, systems and methods

We require information from authors about some types of materials, experimental systems and methods used in many studies. Here, indicate whether each material, system or method listed is relevant to your study. If you are not sure if a list item applies to your research, read the appropriate section before selecting a response.

Materials & experimental systems

n/a	Involved in the study
☐	☒ Antibodies
☒	☐ Eukaryotic cell lines
☒	☐ Palaeontology and archaeology
☒	☐ Animals and other organisms
☐	☒ Clinical data
☒	☐ Dual use research of concern

Methods

n/a	Involved in the study
☒	☐ ChIP-seq
☐	☒ Flow cytometry
☒	☐ MRI-based neuroimaging

Antibodies

Antibodies used

Immunohistochemistry (IHC) studies for PD-L1 staining in malignant cells: PD-L1 anti-human antibody clone 28-8, catalog no. ab205921, dilution 1:100; Abcam, Cambridge, MA, USA.

Flow Cytometry studies: fluorochrome-conjugated monoclonal antibodies against CD45 (BUV395, clone HI30, catalog no. 563792, BD Bioscience, 5μl/sample), CD3 (PerCP-Cy5.5, Clone SK7, catalog no. 340949, 10μl/sample, BD Biosciences), CD8 (AF 700, Clone RPA-T8, catalog no. 557945, BD Biosciences, 5μl/sample), CD4 (BUV496, Clone SK3, catalog no. 612936, BD Biosciences, 5μl/sample), PD1 (BV650, clone EH12, catalog no. 564104, BD Biosciences, 3μl/sample), TIM3 (BV605, clone F38-2E2, catalog no. 345018, Biolegend, 4μl/sample), OX40/CD134 (BV711, clone ACT35, catalog no. 563664, BD Biosciences, 5μl/sample), CTLA4 (BV786, clone BNI3, catalog no. 563931, BD Biosciences, 3μl/sample), TIGIT (FITC, clone MBSA43, catalog no. 11-9500-42, Life Technologies, 5μl/sample), LAG3/CD223 (PE, clone 3DS223H, catalog no. 12-2239-42, eBioscience, 5μl/sample), CD56 (PE-CF594, clone B159, catalog no. 562289, BD Biosciences, 5μl/sample), ICOS/CD278 (PE-Cy7, clone ISA-3, catalog no. 25-9948-42, Life Technologies, 3μl/sample), CD25 (APC-eFluor 780, clone BC96, catalog no. 47-0259-42, Life Technologies, 5μl/sample), FOXP3 (eFluor450, clone PCH101, catalog no. 48-4776-42, Life Technologies, 5μl/sample), Ki67 (APC, clone 20Raj1, catalog no. 17-5699-42, Life Technologies, 5μl/sample) anti-human antibodies. Dead cells were stained using LIVE/DEAD™ Fixable Yellow Dead Cell Stain dye (catalog no. L-34968, Life Technologies, 1μl/sample).

Multiplex Immunofluorescence studies: antibodies against (Panel 1): cytokeratin (clone AE1/AE3, catalog no. M351501-2, dilution 1:300, Dako, Santa Clara, CA), CD3 (catalog no. IS503, dilution 1:100, Dako), CD8 (clone C8/144B, catalog no. MS-457-S, dilution

1:300, Thermo Fisher Scientific), CD68 (clone PG-M1, catalog no. M0875, dilution 1:450, Dako), PD-1 (clone EPR4877-2, catalog no. ab137132, dilution 1:250, Abcam), and PD-L1 (clone E1L3N, catalog no. 13684S, dilution 1:3,000, Cell Signaling Technology); and antibodies against (Panel 2): panel 2: cytokeratin (clone AE1/AE3, catalog no. M351501-2, dilution 1:300, Dako), CD3 (catalog no. IS503, dilution 1:100, Dako), CD8 (clone C8/144B, catalog no. MS-457-S, dilution 1:300, Thermo Fisher Scientific), CD45RO (clone UCHL1, catalog no. PA0146, Cell Signaling Technology), Granzyme B (clone 11F1, catalog no. PA0291, Cell Signaling Technology) and FOXP3 (clone D2W8E, catalog no. 98377S, Cell Signaling Technology). All the markers were stained in sequence using their respective fluorophore containing in the Opal 7 kit (catalog no. NEL797001KT; Akoya Biosciences/PerkinElmer).

Cyanine dye-labelled antibodies: Arginase (Cy5, Clone EPR6672(B), catalog no. ab211961, Abcam, 5ug/ml), CD3 (Cy3, Clone F7.2.38, catalog no. M7254, Dako, 10ug/ml), CD4 (Cy3, Clone EPR6855, catalog no. ab181724, Abcam, 5ug/ml), CD8 (Cy3, Clone C8/144B, catalog no. M7103, Dako, 10ug/ml), CD11b (Cy3, Clone 238439, catalog no. MAB16992, R&D Systems, 10ug/ml), CD14 (Cy3, Clone EPR3652, catalog no. ab209971, Abcam, 5ug/ml), CD15 (Cy3, Clone Carb-3, catalog no. M3631, Dako, 1.5ug/ml), CD16 (Cy5, Clone DJ130c, catalog no. MA1-84008S4, Thermo, 5ug/ml), CD33 (Cy5, Clone 44M12D3, catalog no. NBP2-22377, Novus, 7.5ug/ml), CD56 (Cy5, Clone MRQ-42, catalog no. 156R-OEM0714, Cell Marque, 2ug/ml), CD68 (Cy5, Clone KP-1, catalog no. MS-397-PABX, BioLegend, 0.5ug/ml), CD163 (Cy5, Clone EDHu-1, catalog no. MCA1853, BioRad, 1.25ug/ml), CTLA4 (Cy3, Clone F-8, catalog no. sc-376016, Santa Cruz, 2ug/ml), FOXP3 (Cy5, Clone 206D, catalog no. 320114, BioLegend, 5ug/ml), HLA-DR (Cy5, Clone WR18, catalog no. MA1-80678, Novus, 5ug/ml), Ki67 (Cy3, Clone SP6, catalog no. ab197547, Abcam, 10ug/ml), PD-1 (Cy5, Clone EPR4877(2), catalog no. ab186928, Abcam, 5ug/ml), PD-L1 (Cy5, Clone SP142, catalog no. ab236238, Abcam, 5ug/ml), PanCK (Cy3, Clone PCK26, catalog no. C5992, Sigma, 1.5ug/ml), PanCK (Cy3, Clone AE1, catalog no. cust02300, eBio, 2.5ug/ml).

Validation

Immunohistochemistry (IHC) antibody for PD-L1 staining in malignant cells was previously validated as reported in https://www.ncbi.nlm.nih.gov/pubmed/28719380.

Flow cytometry staining, antibodies were titrated on PBMCs and expanded tumor-infiltrating lymphocytes including unstained controls. The majority of these markers have been previously described in Bentebibel et al., Cancer Discovery 2019 (PMID:30988166) and Cascone et al., Nature Medicine 2021 (PMID: 33603241).

CD45 (BUV395, Clone HI30, Cat. No. 563792) - antibody internally validated by using tumor cells as a negative control and normal donor PBMCs as a positive control. Vendor validation and technical information can be found at; https://www.bdbiosciences.com/content/bdb/paths/generate-tds-document.us.563792.pdf

CD3 (PerCP-Cy5.5, Clone SK7, Cat. No. 340949, BD Biosciences) - antibody internally titrated and validated using normal donor PBMCs and B cells as a negative control. Vendor validation and technical information can be found at; https://www.bdbiosciences.com/content/bdb/paths/generate-tds-document.us.340949.pdf

CD8 (AF 700, Clone RPA-T8, Cat. No. 557945, BD Biosciences) - antibody internally titrated and validated using expanded tumor infiltrating lymphocytes and normal donor PBMCs. B cells were used as a negative control from normal donor PBMCs; Vendor validation and technical information can be found at; https://www.bdbiosciences.com/content/bdb/paths/generate-tds-document.us.557945.pdf

CD4 (BUV496, Clone SK3, Cat. No. 612936, BD Biosciences) - antibody internally titrated and validated using expanded tumor-infiltrating lymphocytes and normal donor PBMCs. B cells were used as a negative control from normal donor PBMCs; Vendor validation and technical information can be found at; https://www.bdbiosciences.com/content/bdb/paths/generate-tds-document.us.612936.pdf

PD1 (BV650, Clone EH12 Cat. No. 564104, BD Biosciences) - antibody internally titrated and validated with respect to differential staining patterns on CD8 T cells from expanded tumor-infiltrating lymphocytes as a positive control and normal donor PBMCs as a negative control; Vendor validation and technical information can be found at; https://www.bdbiosciences.com/en-us/products/reagents/flow-cytometry-reagents/research-reagents/single-color-antibodies-ruo/bv650-mouse-anti-human-cd279-pd-1.564104

TIM3 (BV605, Clone F38-2E2, Cat. No. 345018, Biolegend) - antibody internally titrated and validated with respect to differential staining patterns on CD8 T cells from expanded tumor-infiltrating lymphocytes as a positive control and normal donor PBMCs as a negative control; Vendor validation and technical information can be found at; https://www.biolegend.com/en-us/products/brilliant-violet-605-anti-human-cd366-tim-3-antibody-8606

OX40 (CD134) (BV711, Clone ACT35, Cat. No. 563664, BD Biosciences) - antibody internally validated by gating on T cells from normal donors as a negative control and stimulated CD4+ tumor-infiltrating lymphocytes as a positive control; Vendor validation and technical information can be found at; https://www.bdbiosciences.com/en-us/products/reagents/flow-cytometry-reagents/research-reagents/single-color-antibodies-ruo/bv711-mouse-anti-human-cd134.563664

CTLA4 (BV786, Clone BNI3, Cat. No. 563931, BD Biosciences) - antibody internally validated with respect to differential staining pattern on activated T cells as compared to unactivated T cells from expanded tumor-infiltrating lymphocytes and normal donor PBMCs; Vendor validation and technical information can be found at; https://www.bdbiosciences.com/content/bdb/paths/generate-tds-document.us.563931.pdf

TIGIT (FITC, Clone MBSA43, Cat. No. 11-9500-42, Life Technologies) - antibody internally validated by differential staining on unstimulated T cells from normal donor PBMCs as compared to expression on expanded CD8+ tumor-infiltrating lymphocytes; Vendor validation and technical information can be found at; https://www.thermofisher.com/antibody/product/TIGIT-Antibody-clone-MBSA43-Monoclonal/11-9500-42

LAG3 (PE, Clone 3DS223H, Cat. No. 12-2239-42, Life Technologies) - antibody internally validated with respect to differential staining pattern on CD8 T cells and CD4 T cells from expanded tumor-infiltrating lymphocytes as a positive control as compared to from normal donor PBMCs as a negative control; Vendor validation and technical information can be found at; https://www.thermofisher.com/antibody/product/CD223-LAG-3-Antibody-clone-3DS223H-Monoclonal/12-2239-42

CD56 (PE-CF594, Clone B159, Cat. No. 562289, BD Biosciences) - antibody internally validated by gating on CD3 negative cells from normal donor PBMCs; Vendor validation and technical information can be found at; https://www.bdbiosciences.com/en-us/products/reagents/flow-cytometry-reagents/research-reagents/single-color-antibodies-ruo/pe-cf594-mouse-anti-human-cd56-ncam-1.562289

ICOS (CD278) (PE-Cy7, Clone ISA-3, Cat. No. 25-9948-42, Life Technologies) - antibody internally validated with respect to differential staining pattern on activated T cells as compared to unactivated T cells from expanded tumor-infiltrating lymphocytes and normal donor PBMCs; Vendor validation and technical information can be found at; https://www.thermofisher.com/antibody/product/CD278-ICOS-Antibody-clone-ISA-3-Monoclonal/25-9948-42

CD25 (APC-eFluor780, Clone BC96, Cat. No. 47-0259-42, Life Technologies) - antibody internally validated by assessing differential expression on activated and non-activated T cells; Vendor validation and technical information can be found at; https://www.thermofisher.com/antibody/product/CD25-Antibody-clone-BC96-Monoclonal/47-0259-42

FOXP3 (eFluor450, Clone PCH101, Cat. No. 48-4776-42, Life Technologies) - antibody internally validated by gating on CD45+CD3+CD4+ T cells from normal donor PBMCs; Vendor validation and technical information can be found at; https://www.thermofisher.com/antibody/product/FOXP3-Antibody-clone-PCH101-Monoclonal/48-4776-42
Ki67 (APC, Clone 20Raj1, Cat. No. 17-5699-42, Life Technologies) - antibody internally validated by gating on T cells from normal donor PBMCs as a negative control and expanded tumor-infiltrating lymphocytes as a positive control; Vendor validation and technical information can be found at; https://www.thermofisher.com/antibody/product/Ki-67-Antibody-clone-20Raj1-Monoclonal/17-5699-42

Multiplex immunofluorescence antibodies were previously validated as reported in Parra ER, Ferrufino-Schmidt MC, Tamegnon A, Zhang J, Solis L, Jiang M, Ibarguen H, Haymaker C, Lee JJ, Bernatchez C, Wistuba II. Immuno-profiling and cellular spatial analysis using five immune oncology multiplex immunofluorescence panels for paraffin tumor tissue. Sci Rep. 2021 Apr 19;11(1):8511. doi: 10.1038/s41598-021-88156-0. PMID: 33875760; PMCID: PMC8055659. https://www.ncbi.nlm.nih.gov/pmc/articles/PMC8055659/

Clinical data

Policy information about clinical studies
All manuscripts should comply with the ICMJEguidelines for publication of clinical research and a completedCONSORT checklist must be included with all submissions.

Clinical trial registration	NCT3680521
Study protocol	https://clinicaltrials.gov/ProvidedDocs/21/NCT03680521/Prot_00.pdf
Data collection	September 2018 to February 2020, 25 patients were enrolled at the University of Texas MD Anderson Cancer Center, houston, TX
Outcomes	Primary Endpoint: Percentage of patients achieving a point in time objective response (either CR or PR) prior to surgery.<br>Secondary Endpoints:<br>Safety characterized by type, incidence, severity, timing, seriousness and relationship to study treatment of adverse events (AEs) and laboratory abnormalities.<br>- Descriptive characterization of immune cell populations in the tumor and/or peripheral blood at baseline.<br>- Temporal changes in PD-L1 expression, selected cytokins and immune cell populations in the tumor and/or peripheral blood (including myeloid-derived suppressor cells [MDSCs], regulatory T-cells [Tregs], CD4+ [helper] and CD8+ [cytotoxic] T-cells, and the ratio of Type1:Type2 tumor associated macrophages).<br>- Blood plasma concentration of sitravatinib.<br>- Time-to-surgery.<br>- Disease free-survival (DFS).

Flow Cytometry

Plots

Confirm that:

- [x] The axis labels state the marker and fluorochrome used (e.g. CD4-FITC).
- [x] The axis scales are clearly visible. Include numbers along axes only for bottom left plot of group (a 'group' is an analysis of identical markers).
- [x] All plots are contour plots with outliers or pseudocolor plots.
- [x] A numerical value for number of cells or percentage (with statistics) is provided.

Methodology

Sample preparation	Fresh tumor tissue, and in some cases normal kidney samples, were disaggregated using a medimachine and subsequent filtering to generate a single cell suspension for staining. PBMCs were thawed, washed and resuspended for staining. Surface staining was performed in FACS Wash Buffer (lX DPBS with 1% Bovine Serum Albumin) for 30 min on ice using fluorochrome-conjugated monoclonal antibodies from BD Biosciences, Biolegend, and eBioscience. Cells were fixed in 1% paraformaldehyde solution for 20min at room temperature following surface staining. For panels containing transcription factors, cells were fixed and permeabilized using the BD Transcription factor kit according to the manufacturer's instructions. A complete list of the antibodies, catalog numbers, company and clones used are available. Dead cells were stained using AQUA live/dead dye (Invitrogen)and excluded from the analysis.
Instrument	BD Fortessa X20
Software	BD FACSDiva software v8.0.1. was used for data acquisition. FlowJo v. 10.7.1 was used for all flow cytometry analysis.
Cell population abundance	No cells were sorted in this study.
Gating strategy	Cells were initially gated using FSC-A v SSC-A followed by singlet gates using SSC-Av SSC-H. Single cells were then gated for exclusion of dead cells. A QC metric of 100 events was required in the immediate parental gate for any subgating.

- [x] Tick this box to confirm that a figure exemplifying the gating strategy is provided in the Supplementary Information.
